# Supplementary material for: Systemic immune challenge exacerbates neurodegeneration in a model of neurological lysosomal disease
Source: EMBO Mol Med. 2024 Jun 18;16(7):7. doi: 10.1038/s44321-024-00092-4 (PMC11251277; doi:10.1038/s44321-024-00092-4)
Supplement: Supplementary file 1 — Table EV1 [file 44321_2024_92_MOESM1_ESM.docx]

**Table EV1:** **Gene expression assays used to assess the expression of pro-inflammatory genes in mouse tissue samples**

| Gene | Assay ID |
| --- | --- |
| *Gapdh* | Mm99999915 g1 |
| *Il1β* | Mm00434228_m1 |
| *Ccl2* | Mm00441242_m1 |
| *Tlr3* | Mm01207404_m1 |
| *Nlrp3* | Mm00840904_m1 |
| *Tnfα* | Mm00442358_m1 |
| *Pycard* | Mm00445747_g1 |
| *Ccl3* | Mm00441259_g1 |
| *Il1ra* | Mm00446186_m1 |
