# Supplementary material for: Systemic immune challenge exacerbates neurodegeneration in a model of neurological lysosomal disease
Source: EMBO Mol Med. 2024 Jun 18;16(7):7. doi: 10.1038/s44321-024-00092-4 (PMC11251277; doi:10.1038/s44321-024-00092-4)
Supplement: Supplementary file 2 — Table EV2 [file 44321_2024_92_MOESM2_ESM.docx]

**Table EV2: Antibodies used for flow cytometry analysis of spleens**

| Antigen | Conjugate | Dilution |
| --- | --- | --- |
| CD209b | FITC | 1:100 |
| Tim4 | PerCP/eF710 | 1:100 |
| CD169 | A647 | 1:100 |
| Ly6C | AF700 | 1:200 |
| Lineage | APC/eFluor780 |  |
| CD64 | BV421 | 1:200 |
| XCR1 | BV510 | 1:400 |
| CD11c | BV605 | 1:600 |
| CD86 | BV650 | 1:200 |
| CD11b | BV711 | 1:1000 |
| CD45 | BV785 | 1:200 |
| F4/80 | PE | 1:100 |
| MHCII | PE/Cy5 | 1:200 |
| CD68 | PE/Cy7 | 1:100 |
| NK1.1 | APC/eFluor780 | 1:200 |
| CD4 | BV421 | 1:200 |
| CD8 | CD8 | 1:200 |
