# Supplementary figures and images for: Systemic immune challenge exacerbates neurodegeneration in a model of neurological lysosomal disease
Source: EMBO Mol Med. 2024 Jun 18;16(7):7. doi: 10.1038/s44321-024-00092-4 (PMC11251277; doi:10.1038/s44321-024-00092-4)

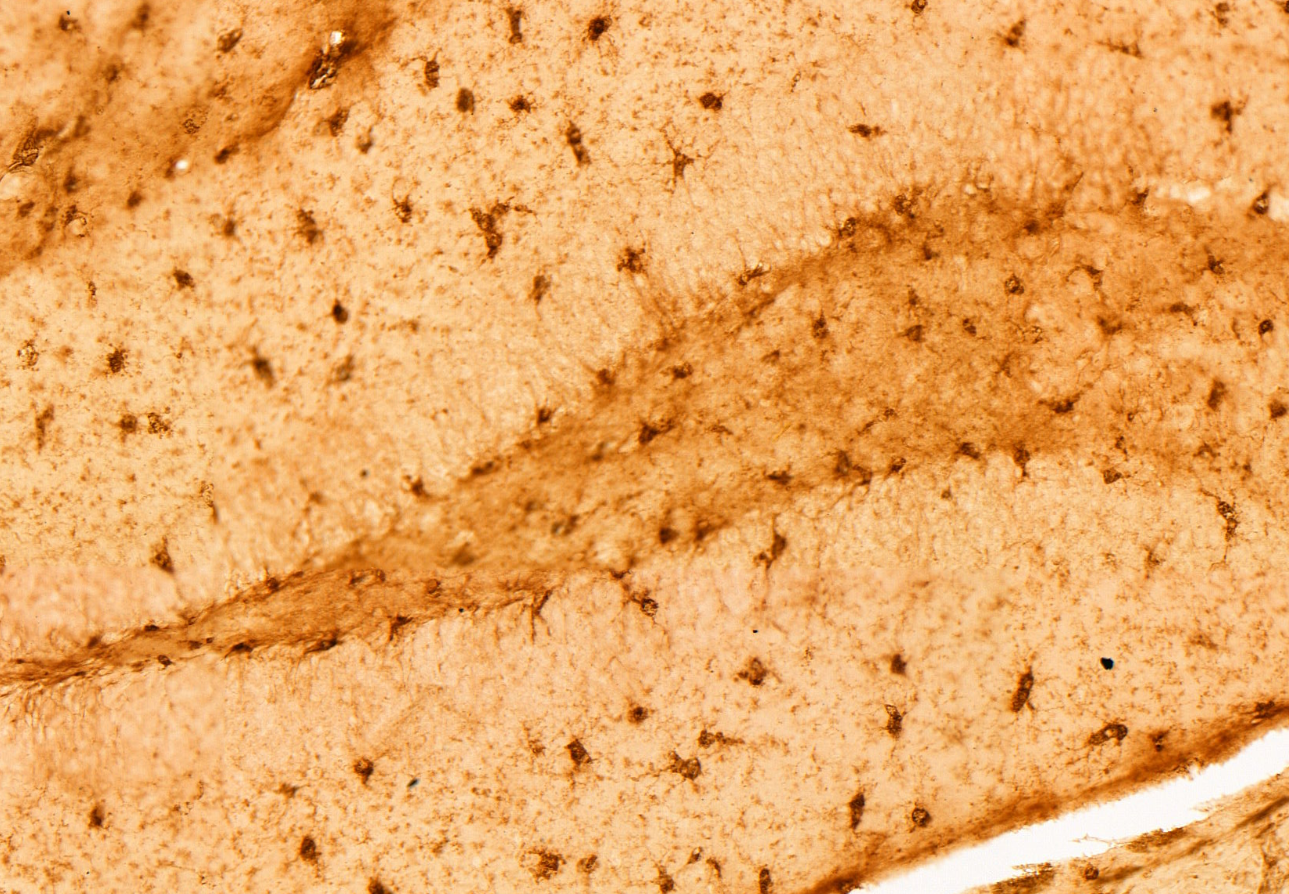

Supplement: Supplementary file 5 — Source data Fig. 2 [file 44321_2024_92_MOESM5_ESM.zip › Figure 2/2A/11.tif]

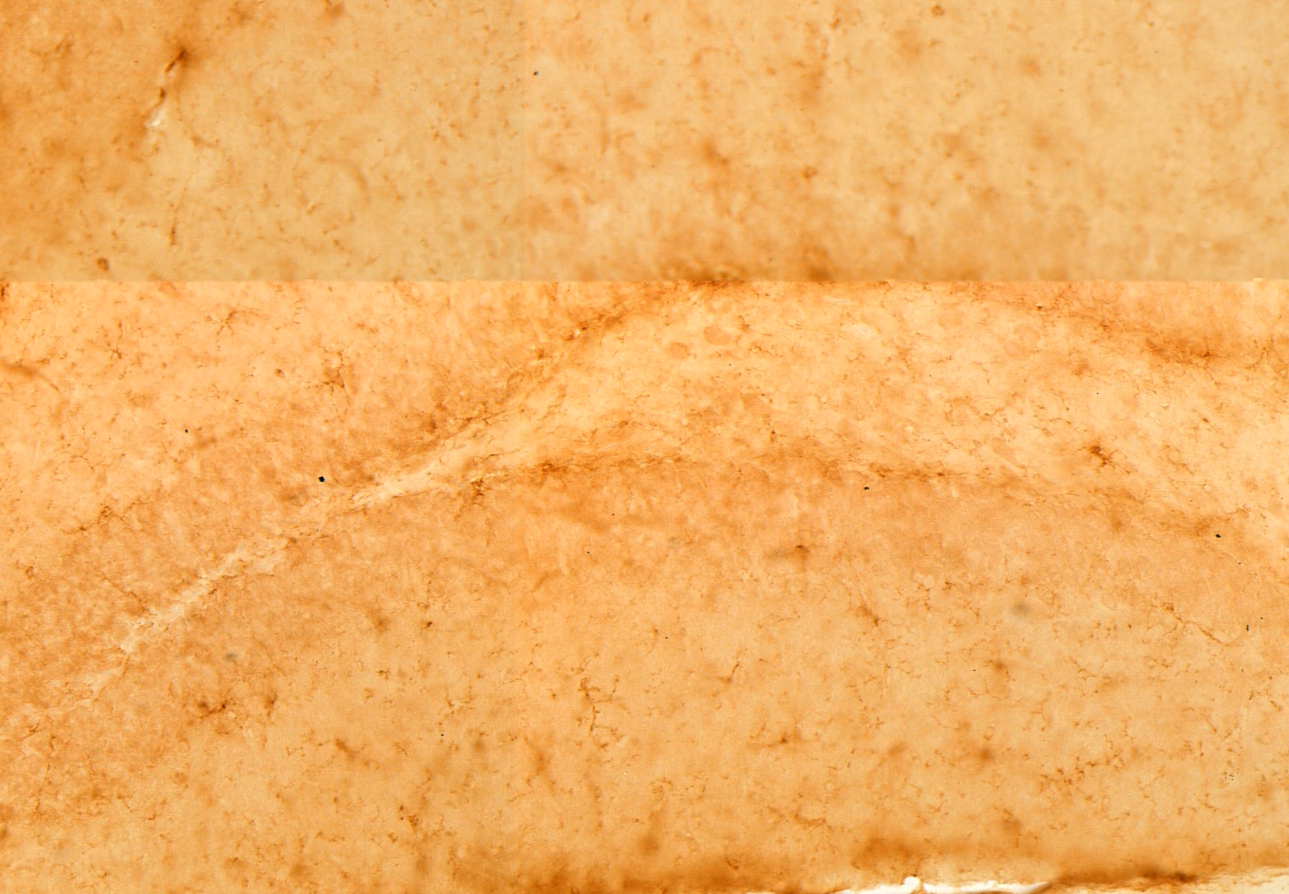

Supplement: Supplementary file 5 — Source data Fig. 2 [file 44321_2024_92_MOESM5_ESM.zip › Figure 2/2A/13.tif]

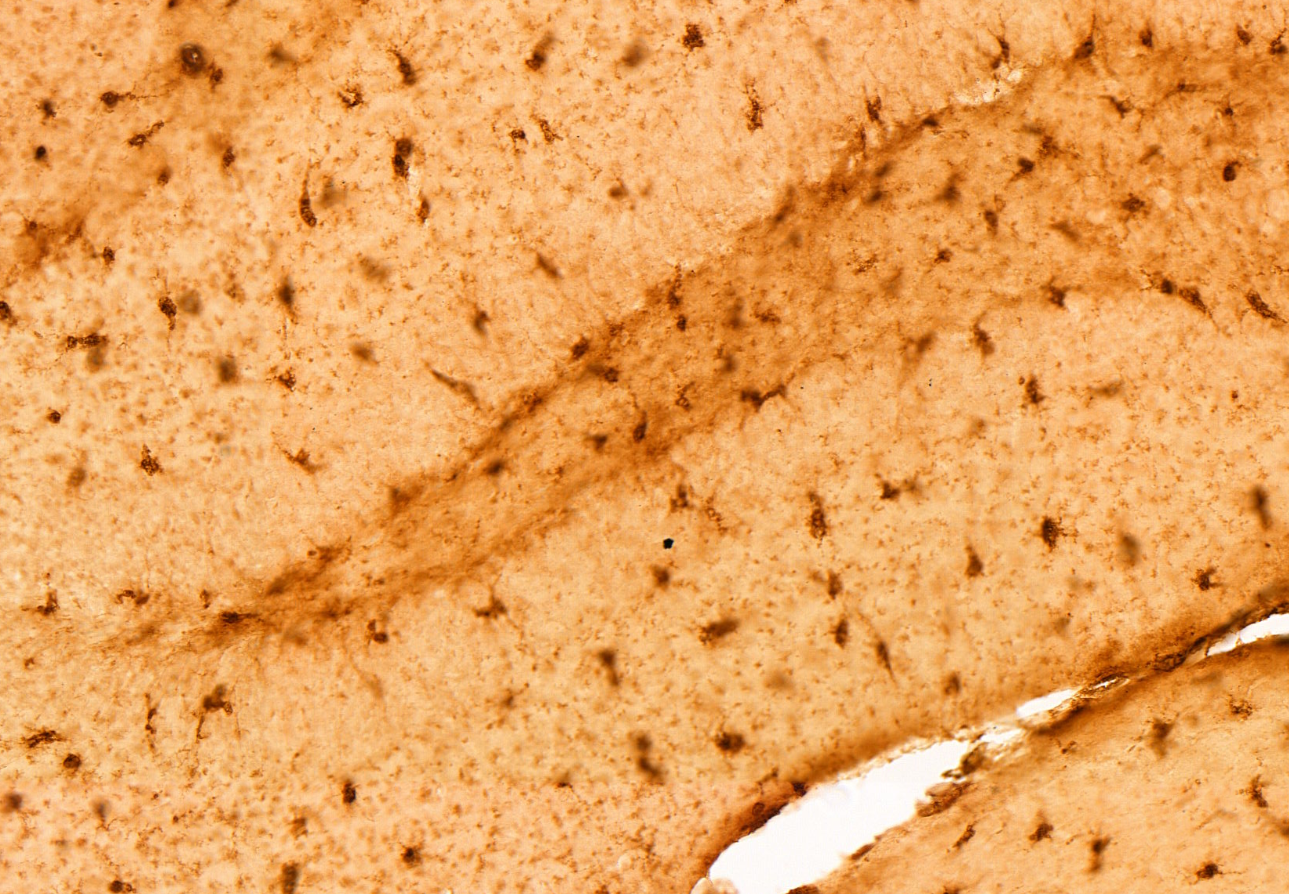

Supplement: Supplementary file 5 — Source data Fig. 2 [file 44321_2024_92_MOESM5_ESM.zip › Figure 2/2A/18.tif]

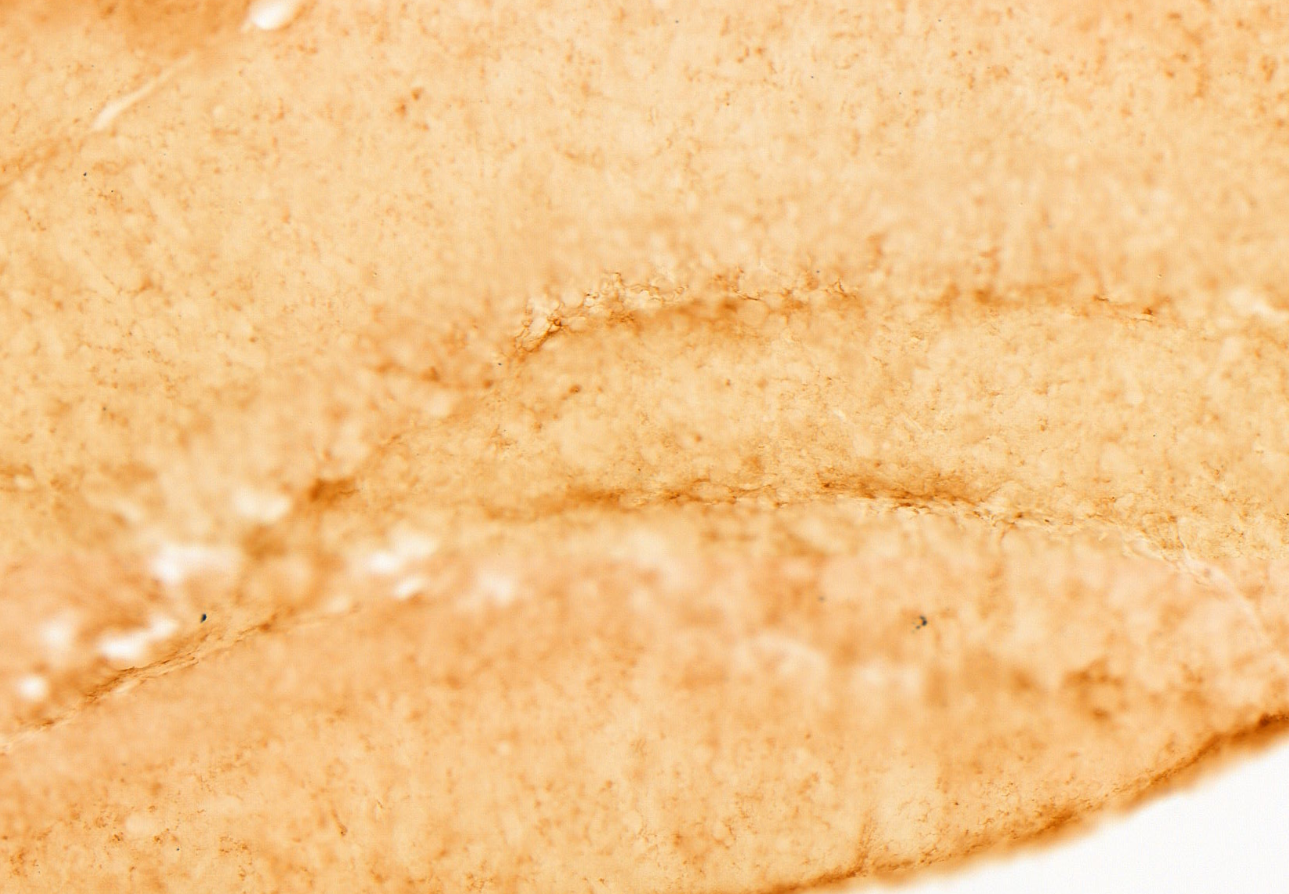

Supplement: Supplementary file 5 — Source data Fig. 2 [file 44321_2024_92_MOESM5_ESM.zip › Figure 2/2A/21.tif]

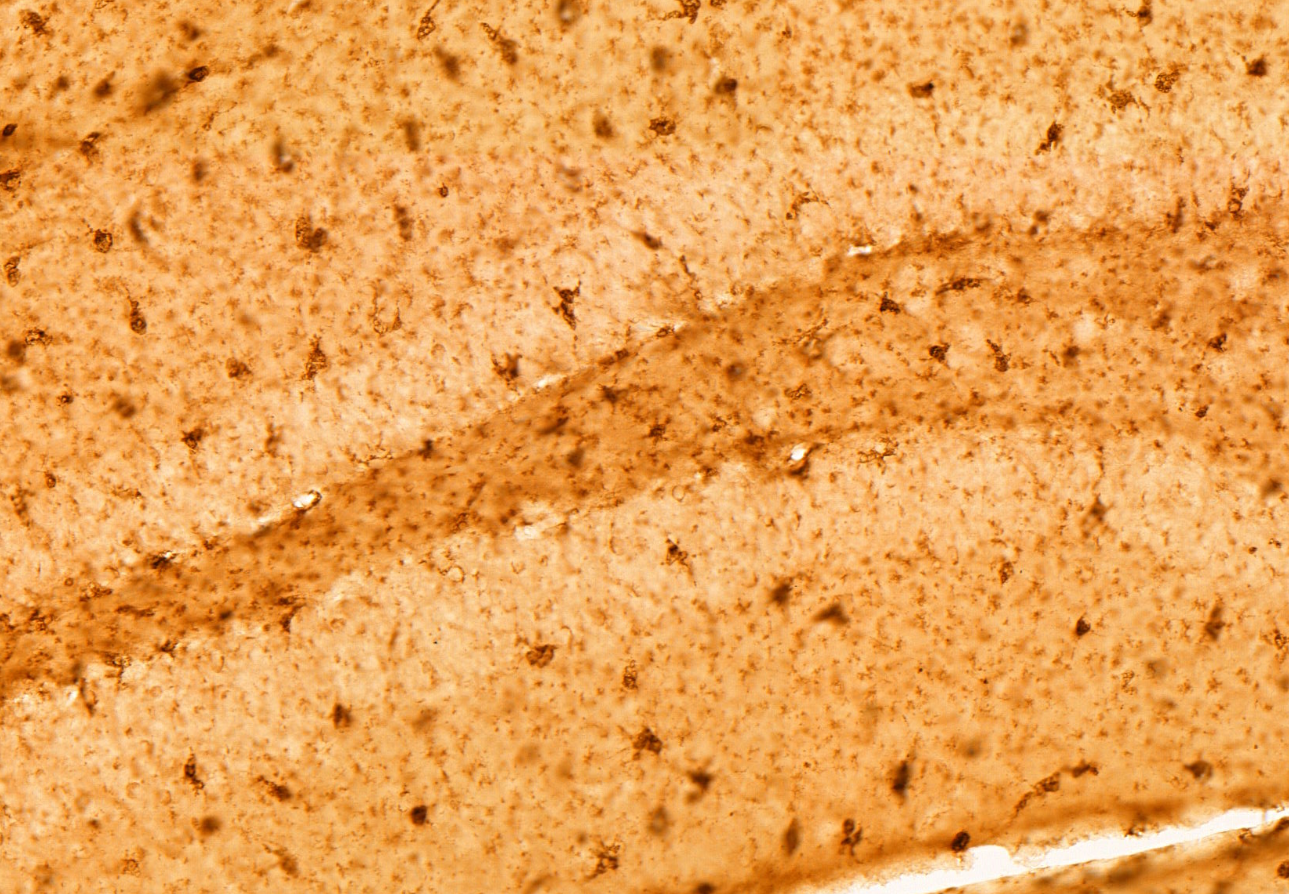

Supplement: Supplementary file 5 — Source data Fig. 2 [file 44321_2024_92_MOESM5_ESM.zip › Figure 2/2A/22.tif]

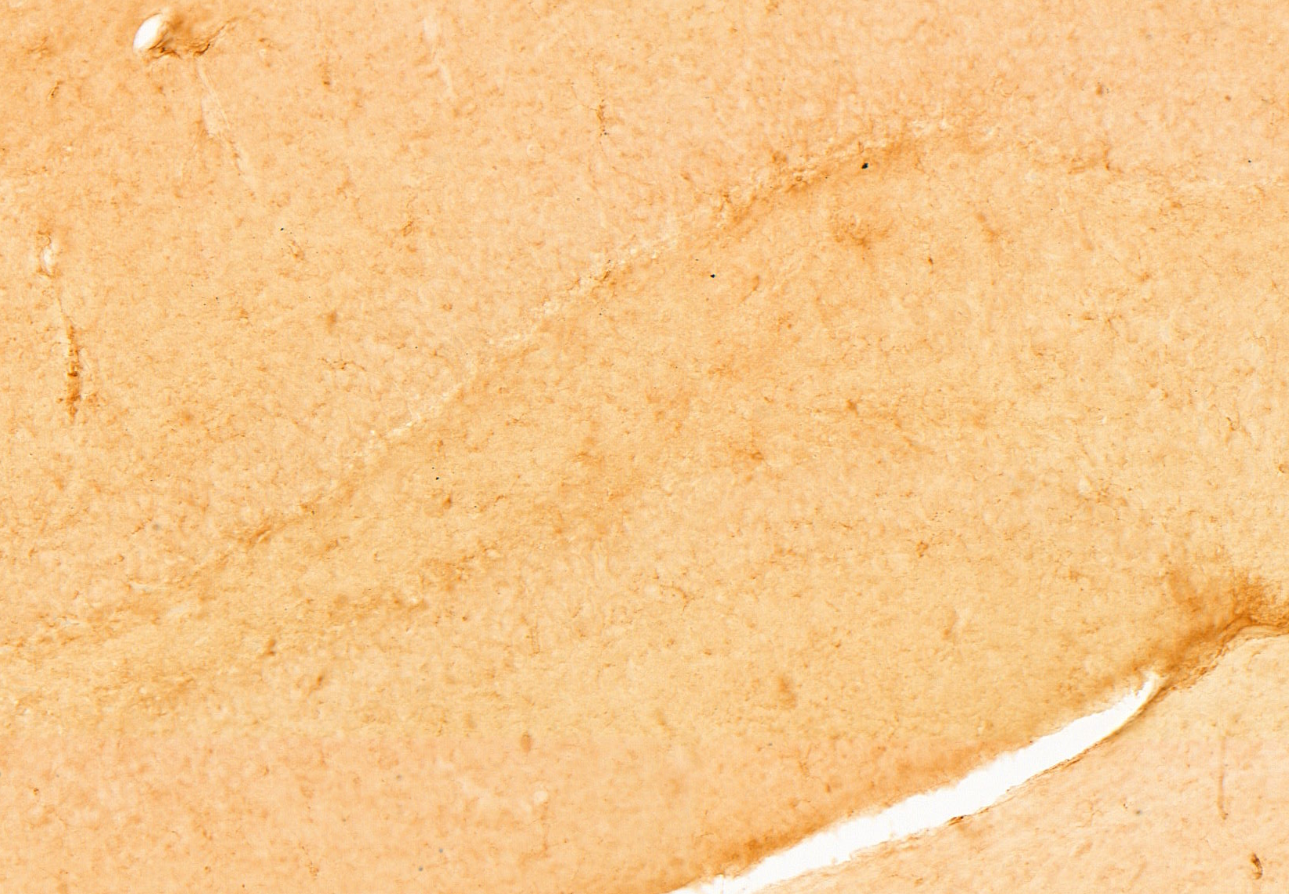

Supplement: Supplementary file 5 — Source data Fig. 2 [file 44321_2024_92_MOESM5_ESM.zip › Figure 2/2A/3.tif]

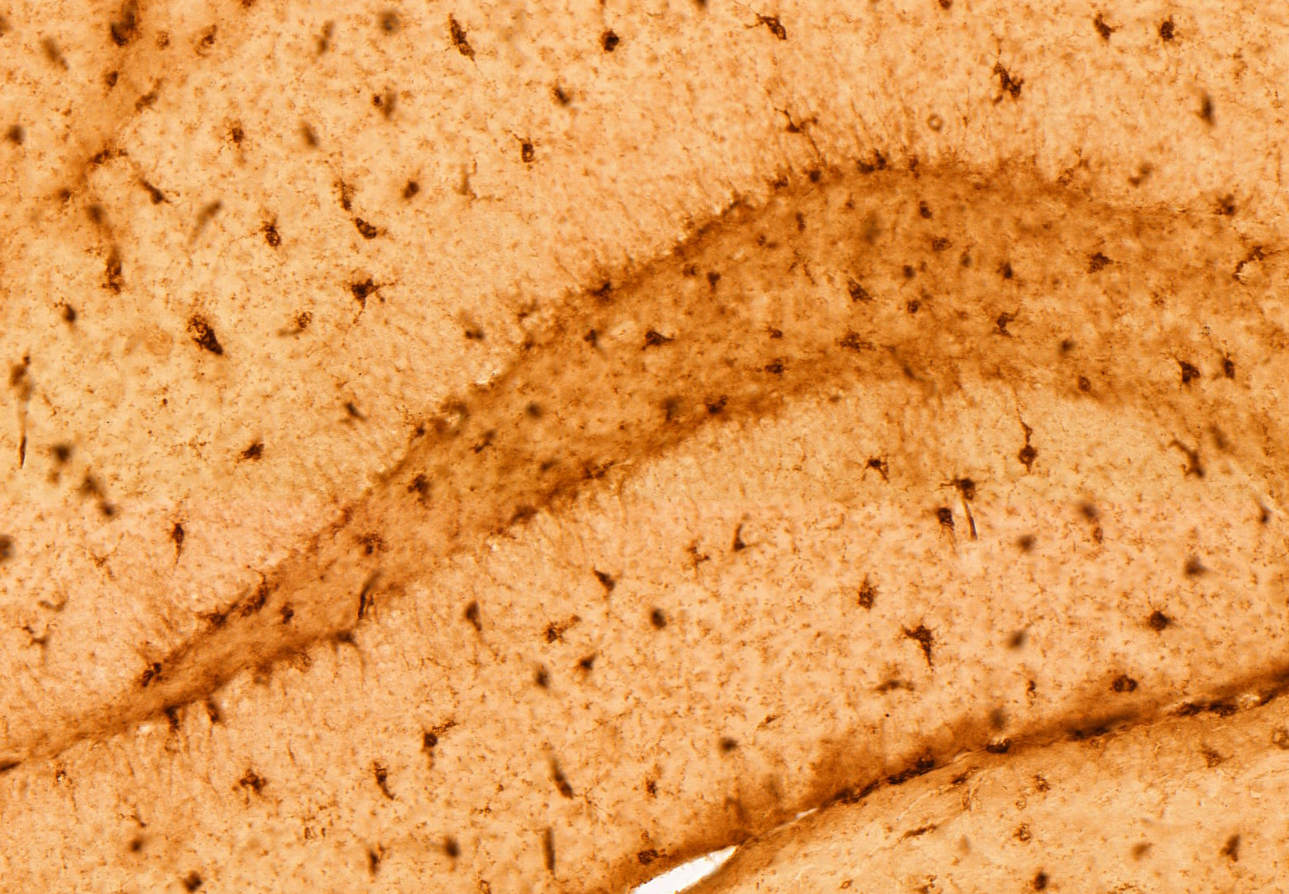

Supplement: Supplementary file 5 — Source data Fig. 2 [file 44321_2024_92_MOESM5_ESM.zip › Figure 2/2A/6.tif]

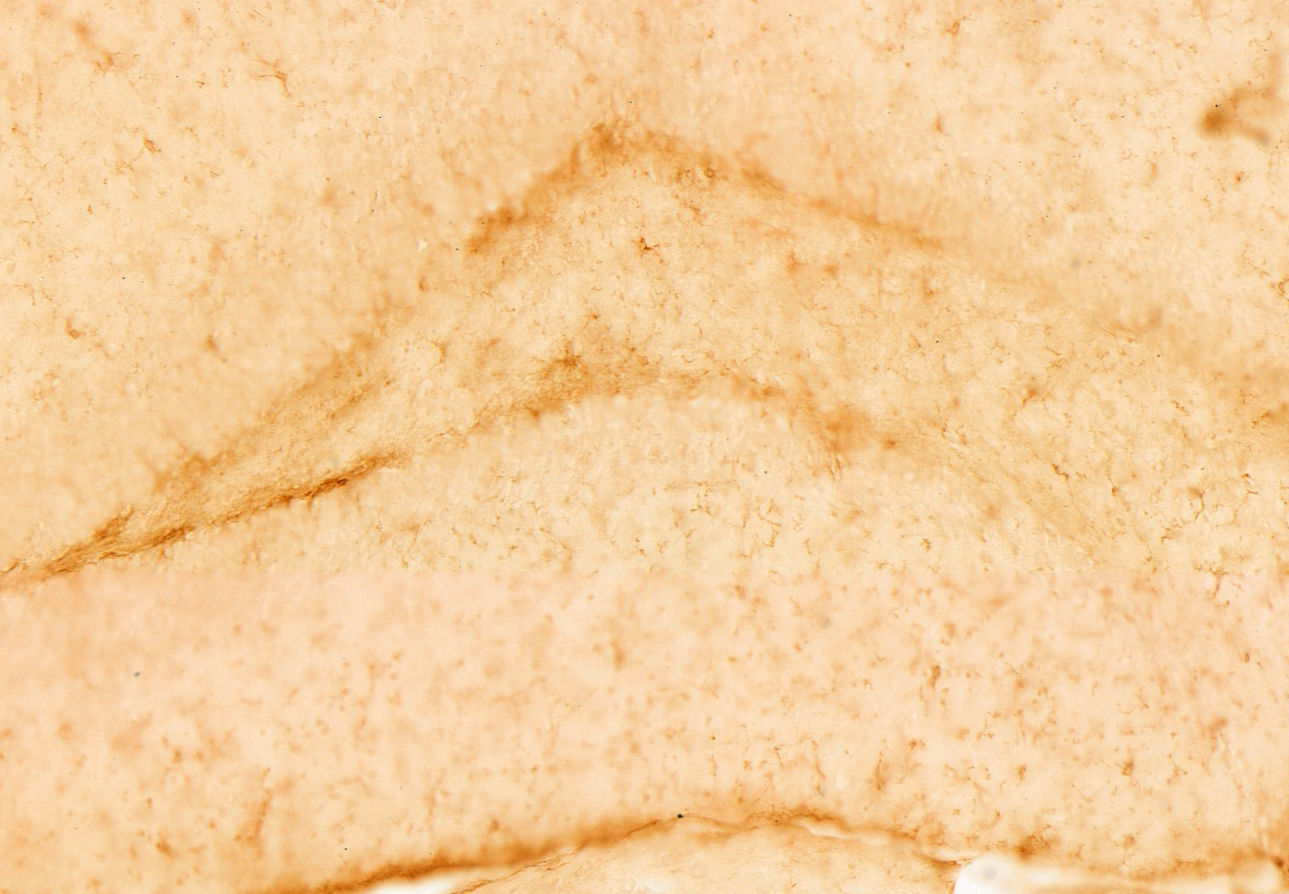

Supplement: Supplementary file 5 — Source data Fig. 2 [file 44321_2024_92_MOESM5_ESM.zip › Figure 2/2A/7.tif]

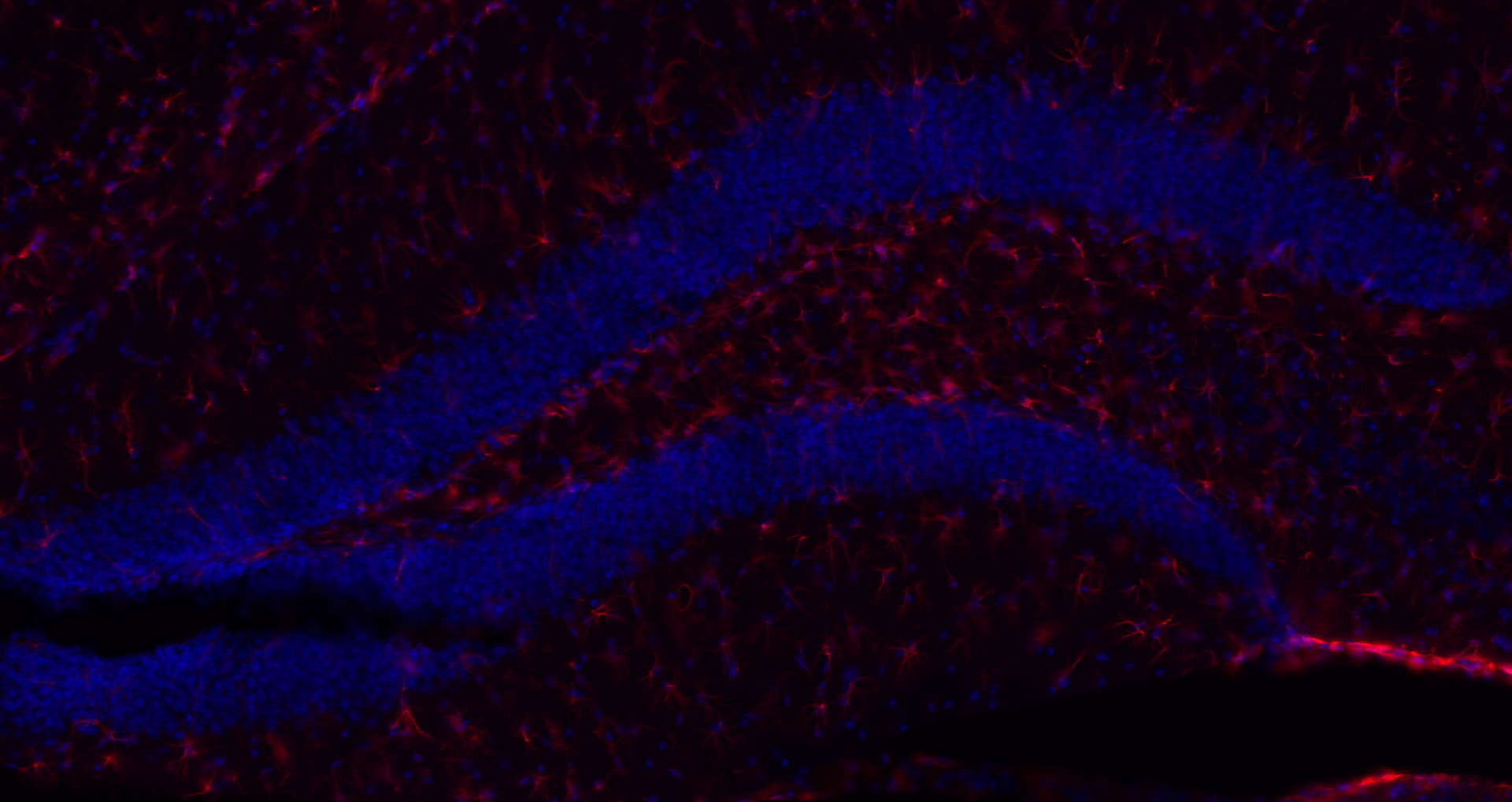

Supplement: Supplementary file 5 — Source data Fig. 2 [file 44321_2024_92_MOESM5_ESM.zip › Figure 2/2B/11.jpg]

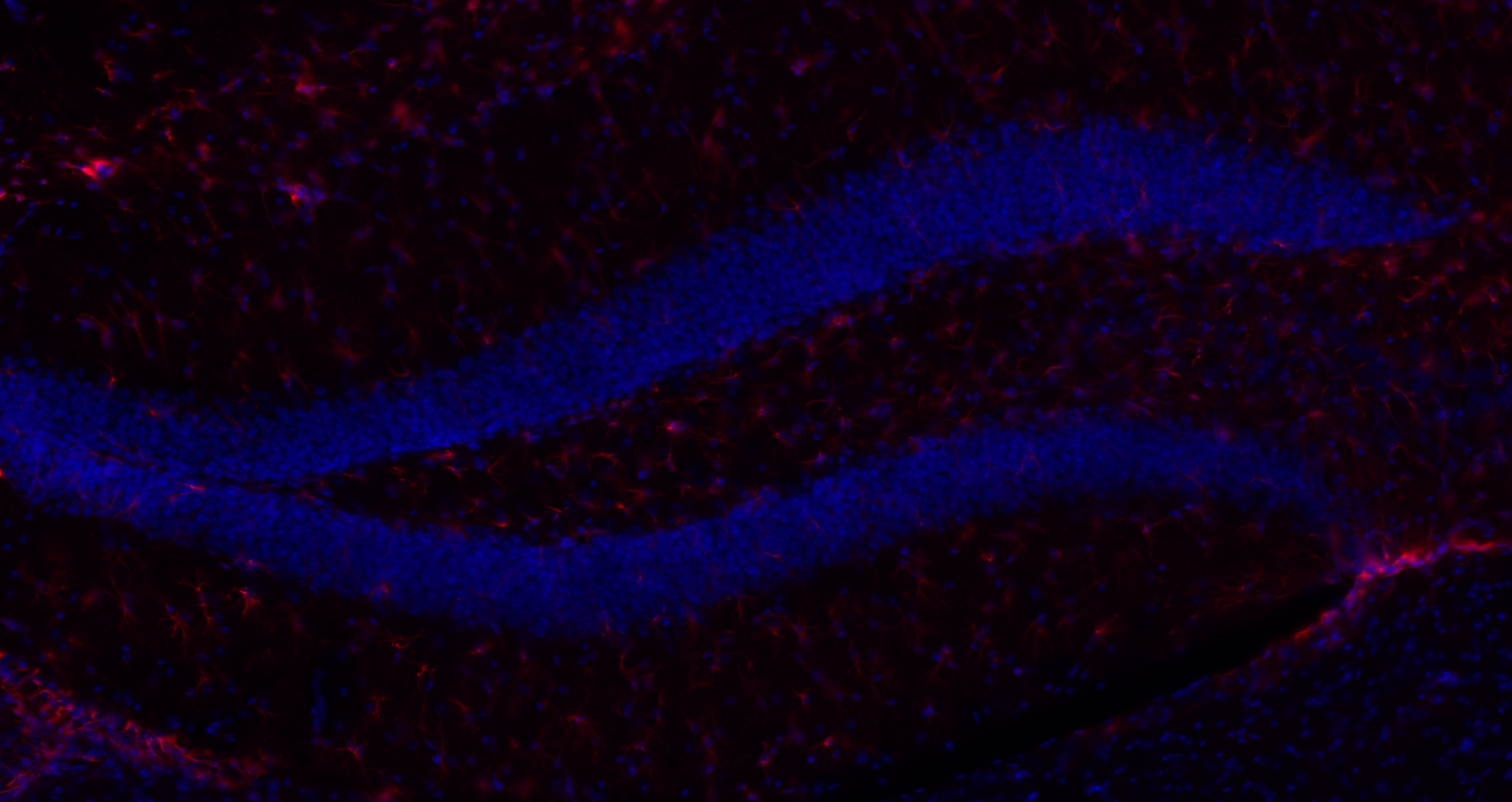

Supplement: Supplementary file 5 — Source data Fig. 2 [file 44321_2024_92_MOESM5_ESM.zip › Figure 2/2B/13.jpg]

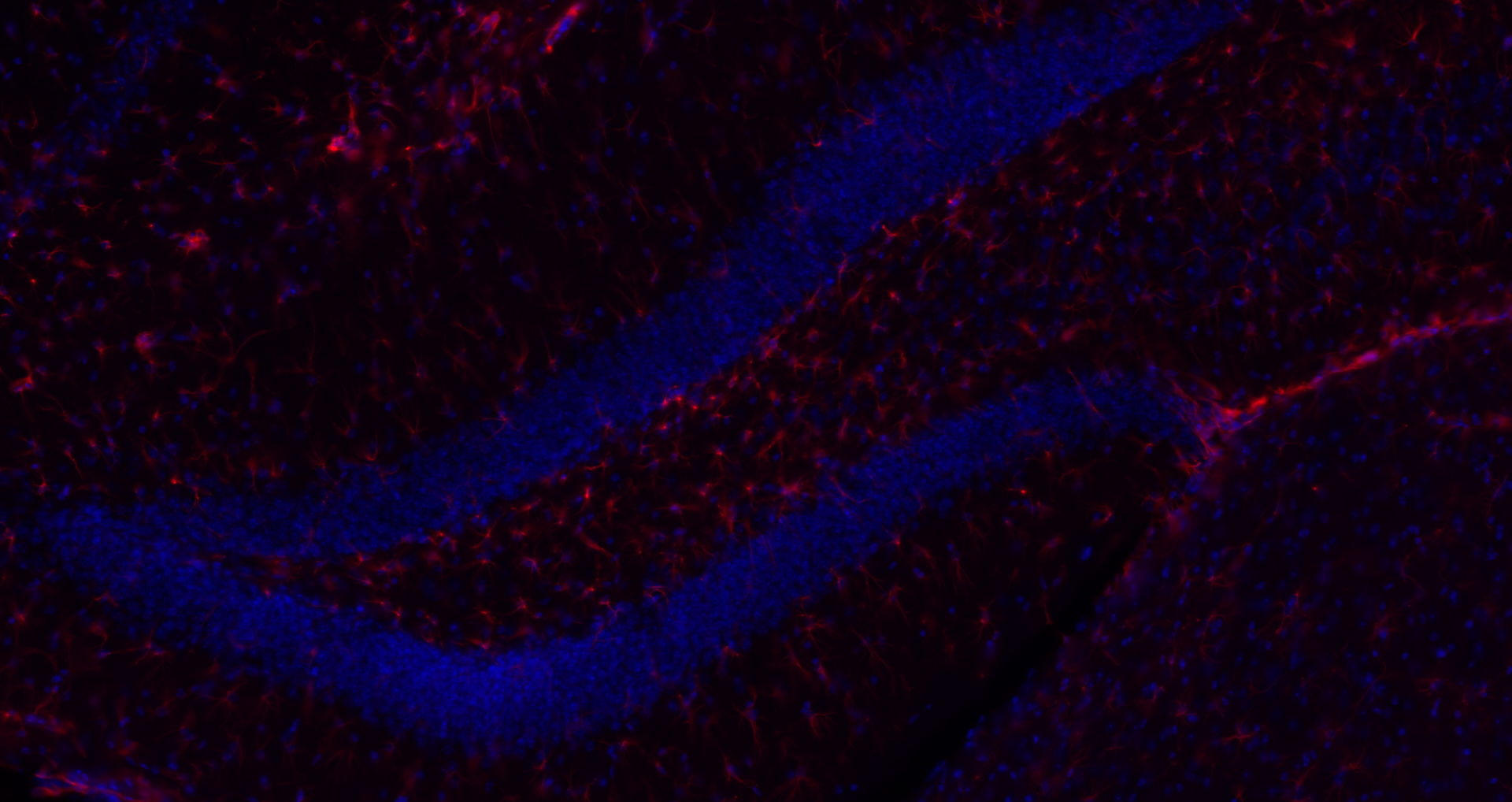

Supplement: Supplementary file 5 — Source data Fig. 2 [file 44321_2024_92_MOESM5_ESM.zip › Figure 2/2B/17.jpg]

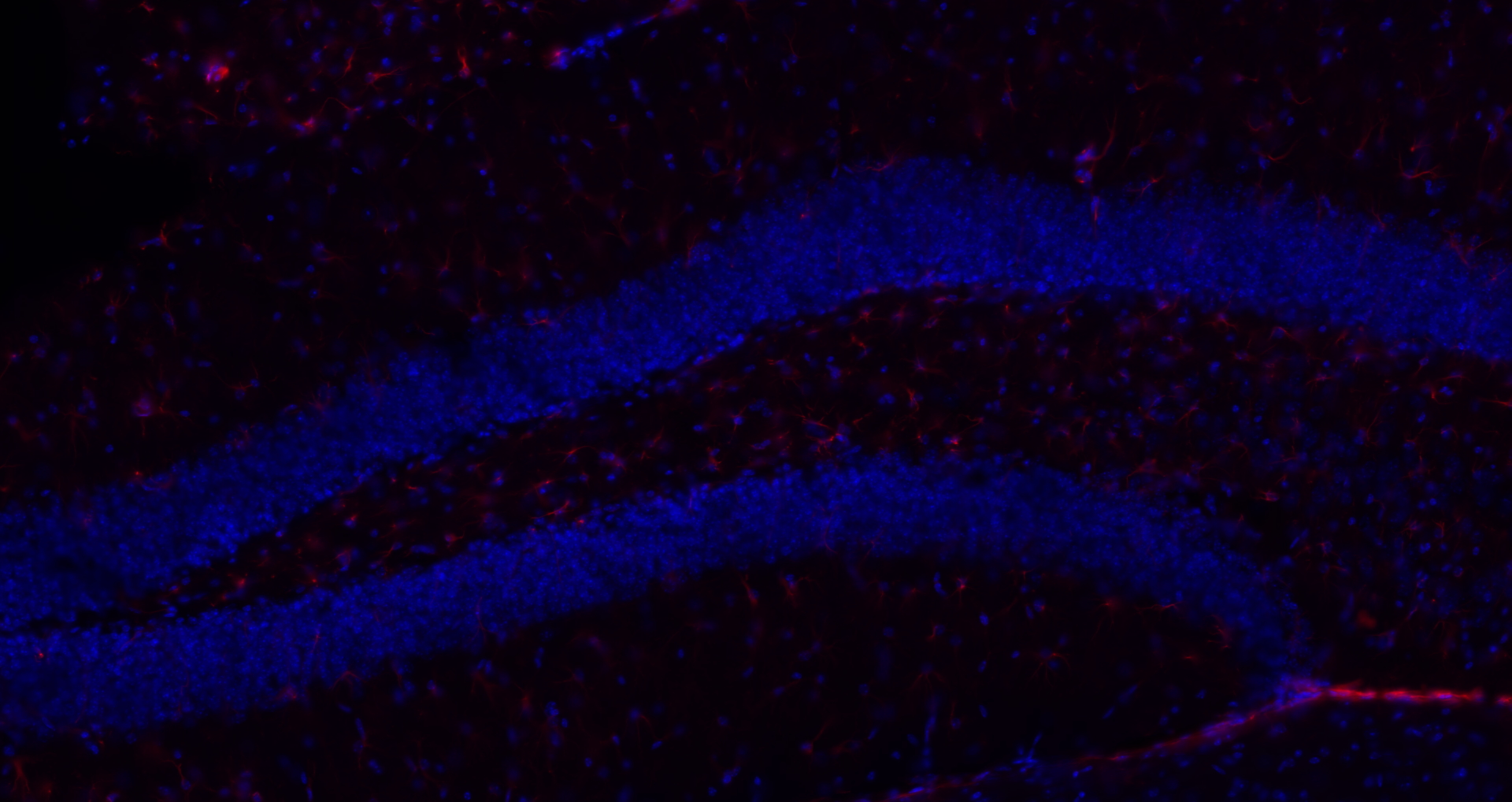

Supplement: Supplementary file 5 — Source data Fig. 2 [file 44321_2024_92_MOESM5_ESM.zip › Figure 2/2B/20.jpg]

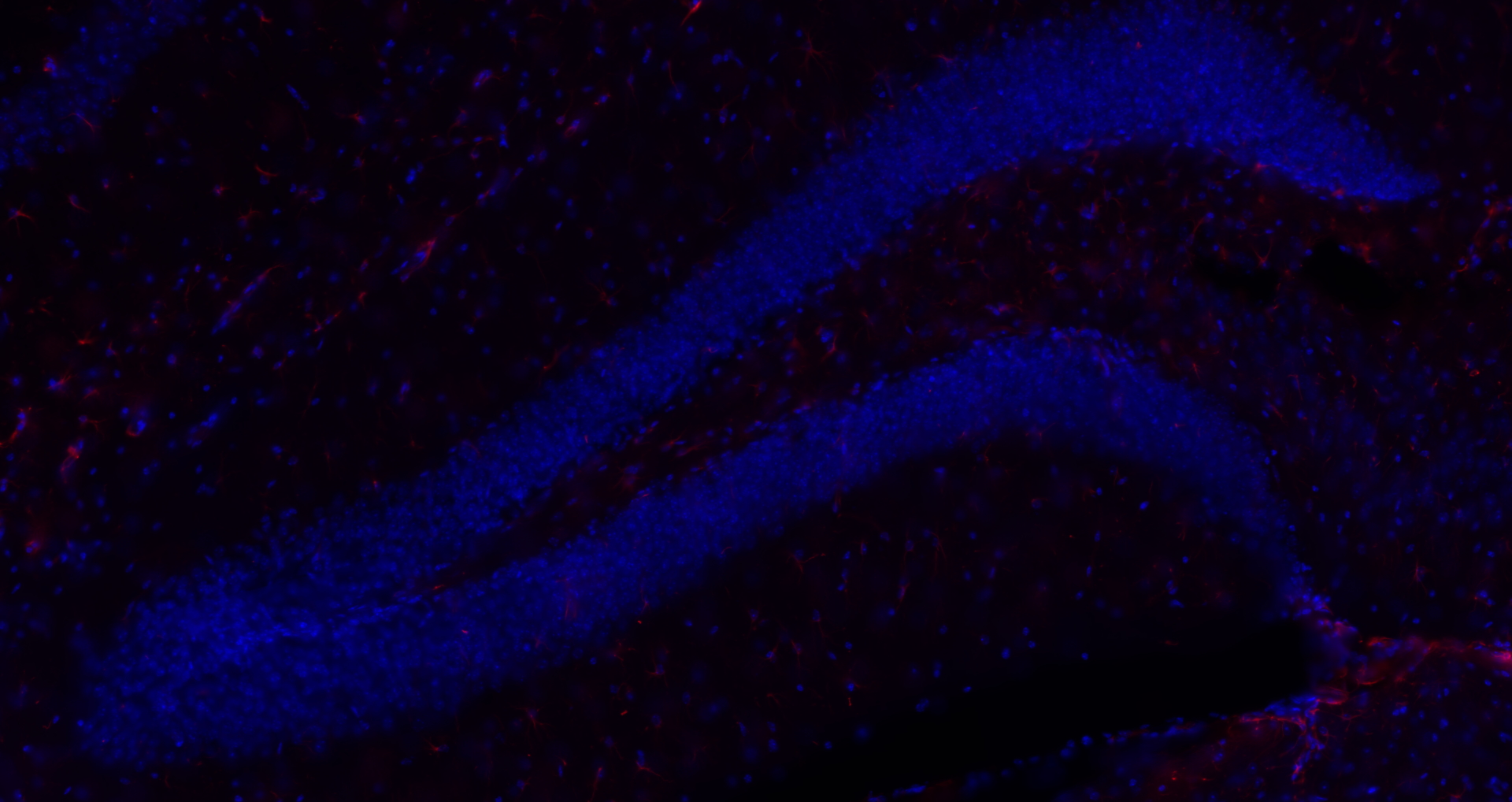

Supplement: Supplementary file 5 — Source data Fig. 2 [file 44321_2024_92_MOESM5_ESM.zip › Figure 2/2B/24.jpg]

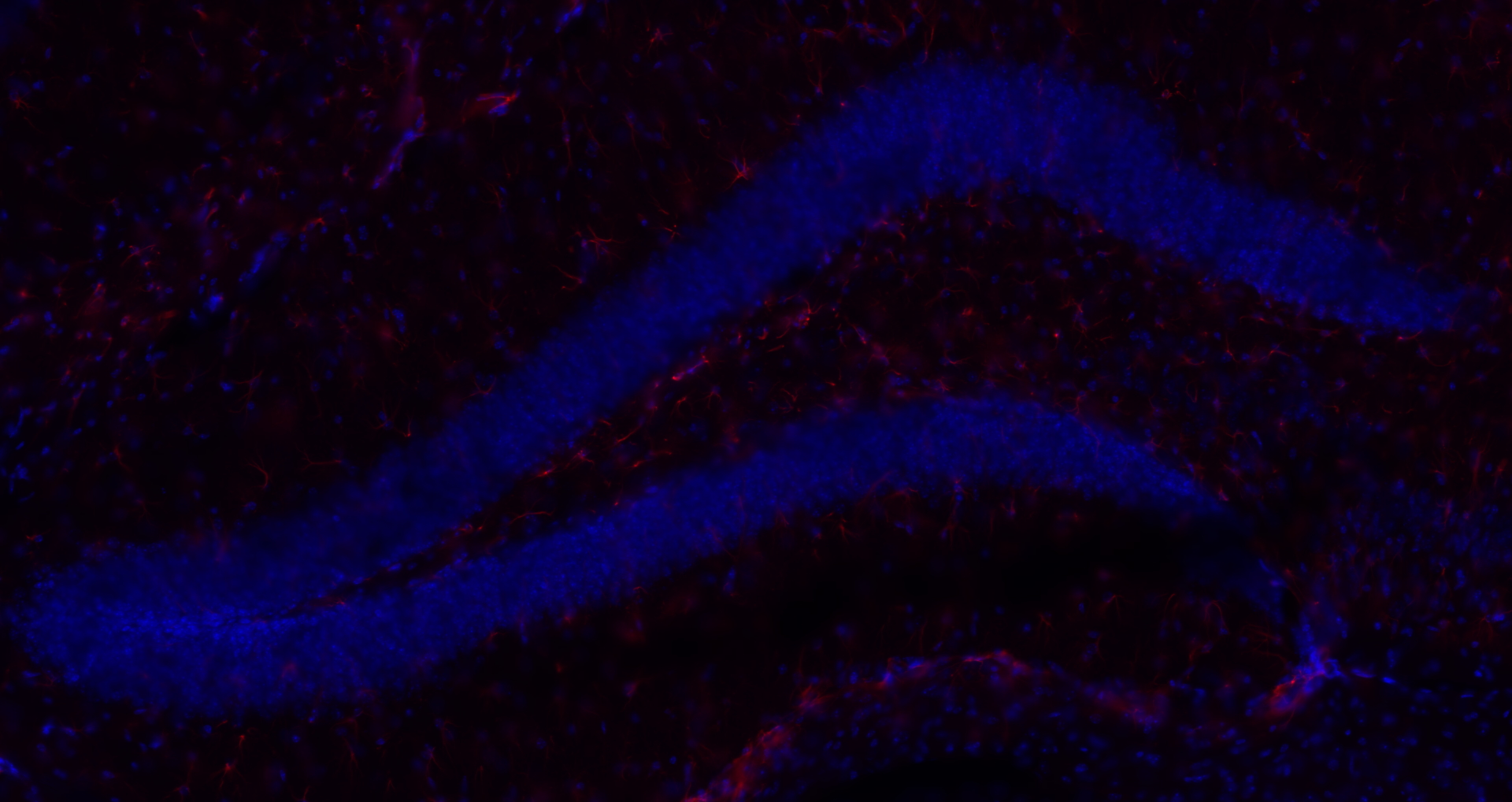

Supplement: Supplementary file 5 — Source data Fig. 2 [file 44321_2024_92_MOESM5_ESM.zip › Figure 2/2B/3.jpg]

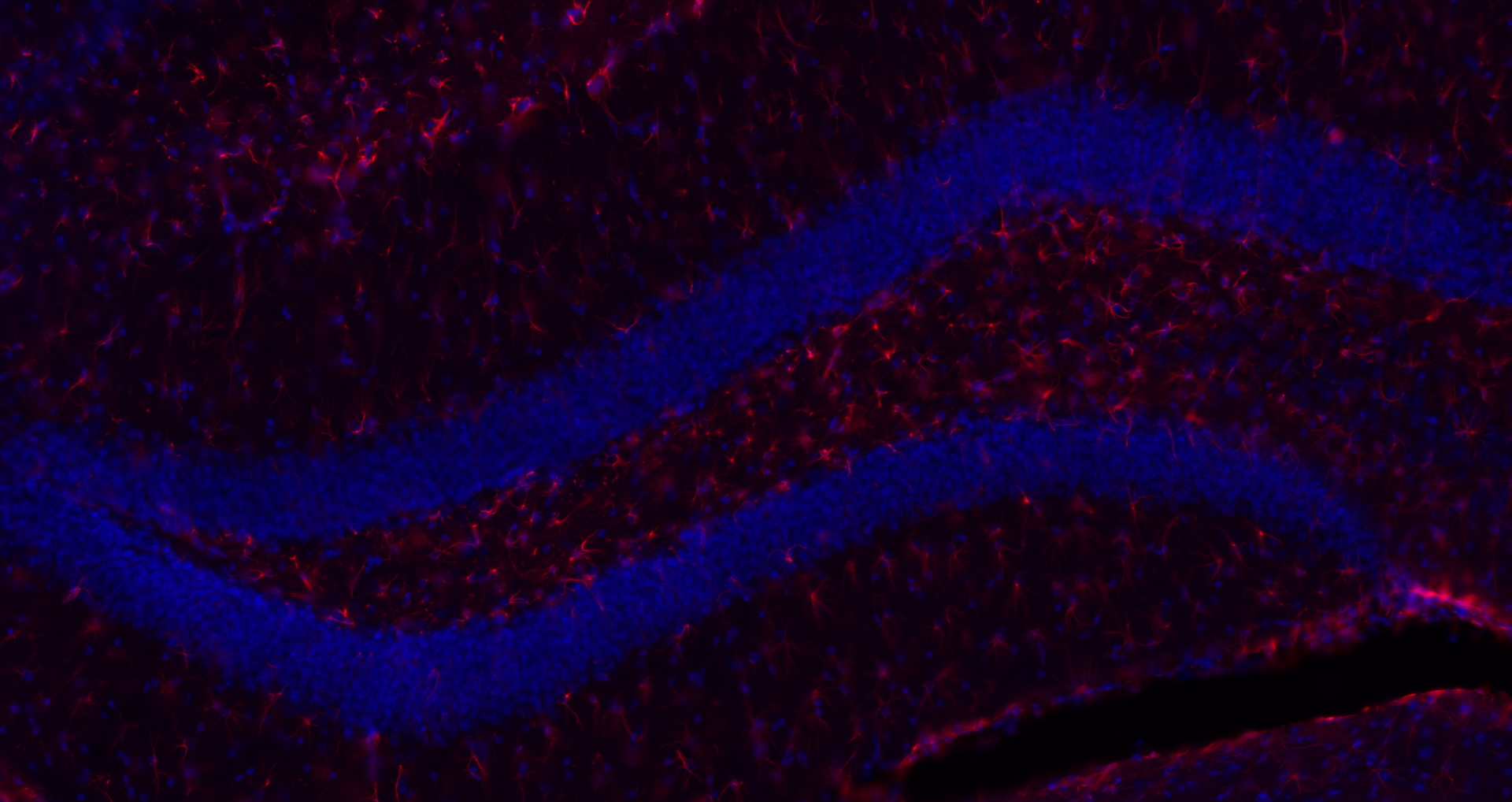

Supplement: Supplementary file 5 — Source data Fig. 2 [file 44321_2024_92_MOESM5_ESM.zip › Figure 2/2B/5.jpg]

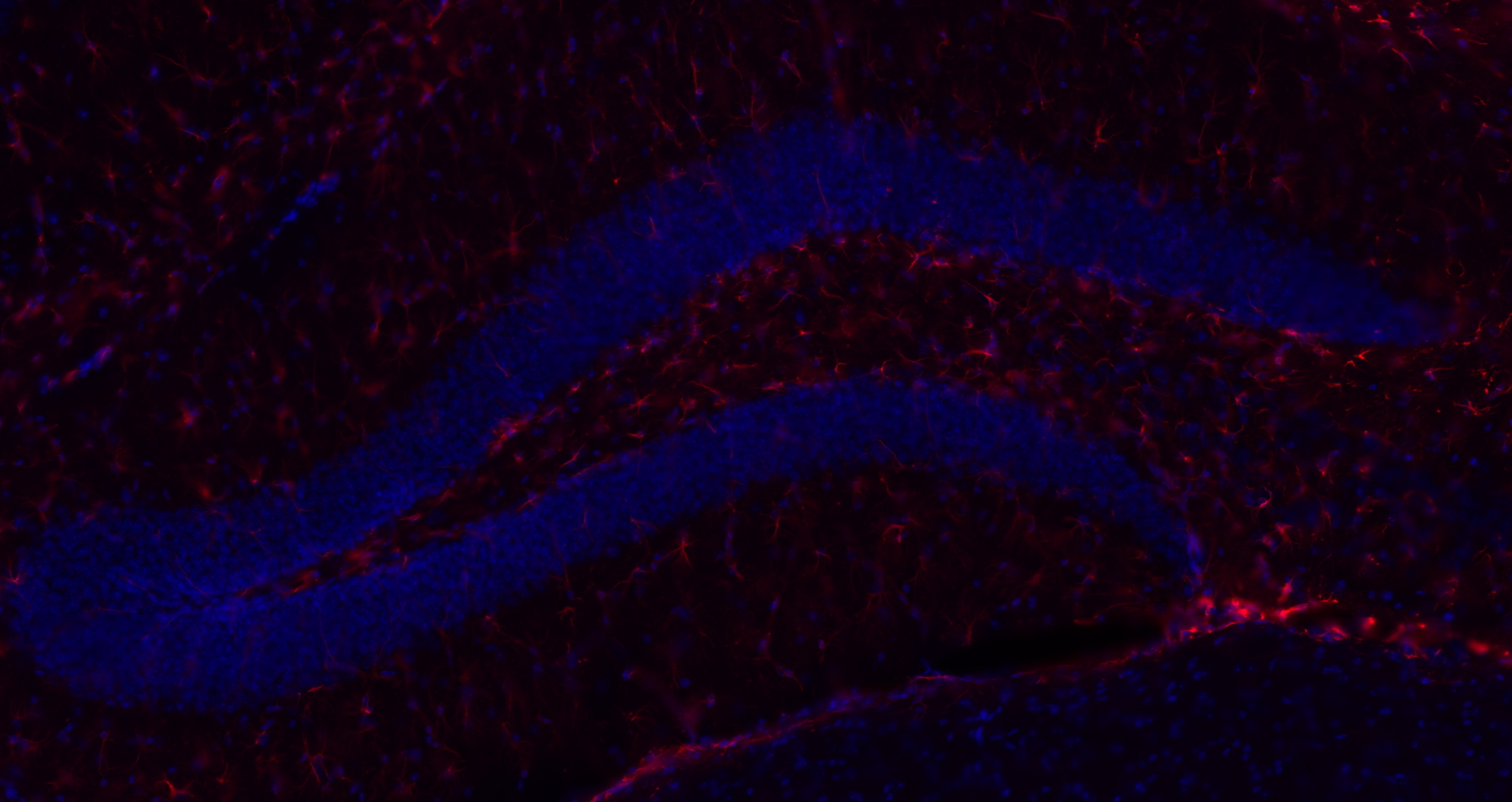

Supplement: Supplementary file 5 — Source data Fig. 2 [file 44321_2024_92_MOESM5_ESM.zip › Figure 2/2B/8.jpg]

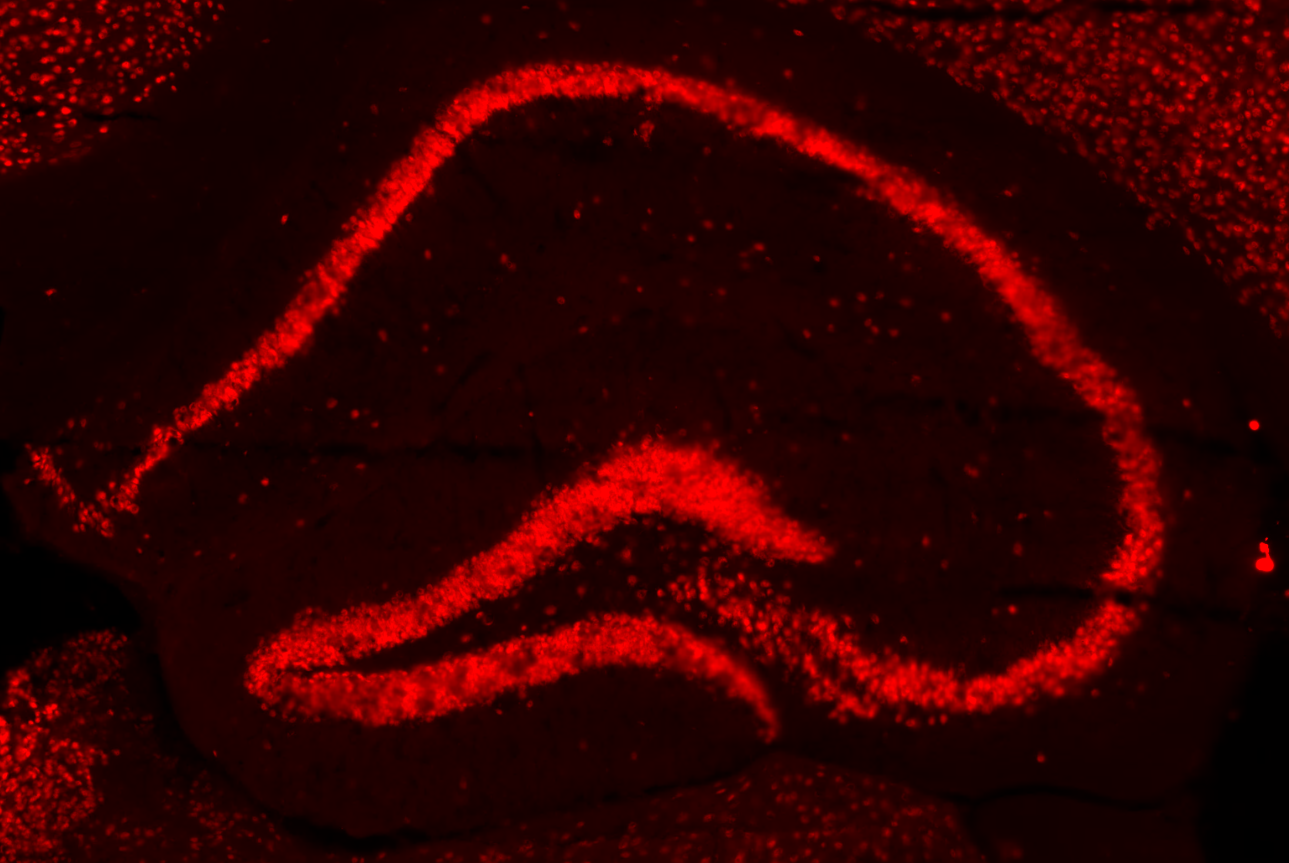

Supplement: Supplementary file 5 — Source data Fig. 2 [file 44321_2024_92_MOESM5_ESM.zip › Figure 2/2C/MPSIIIA PolyIC 3 NeuN.tif]

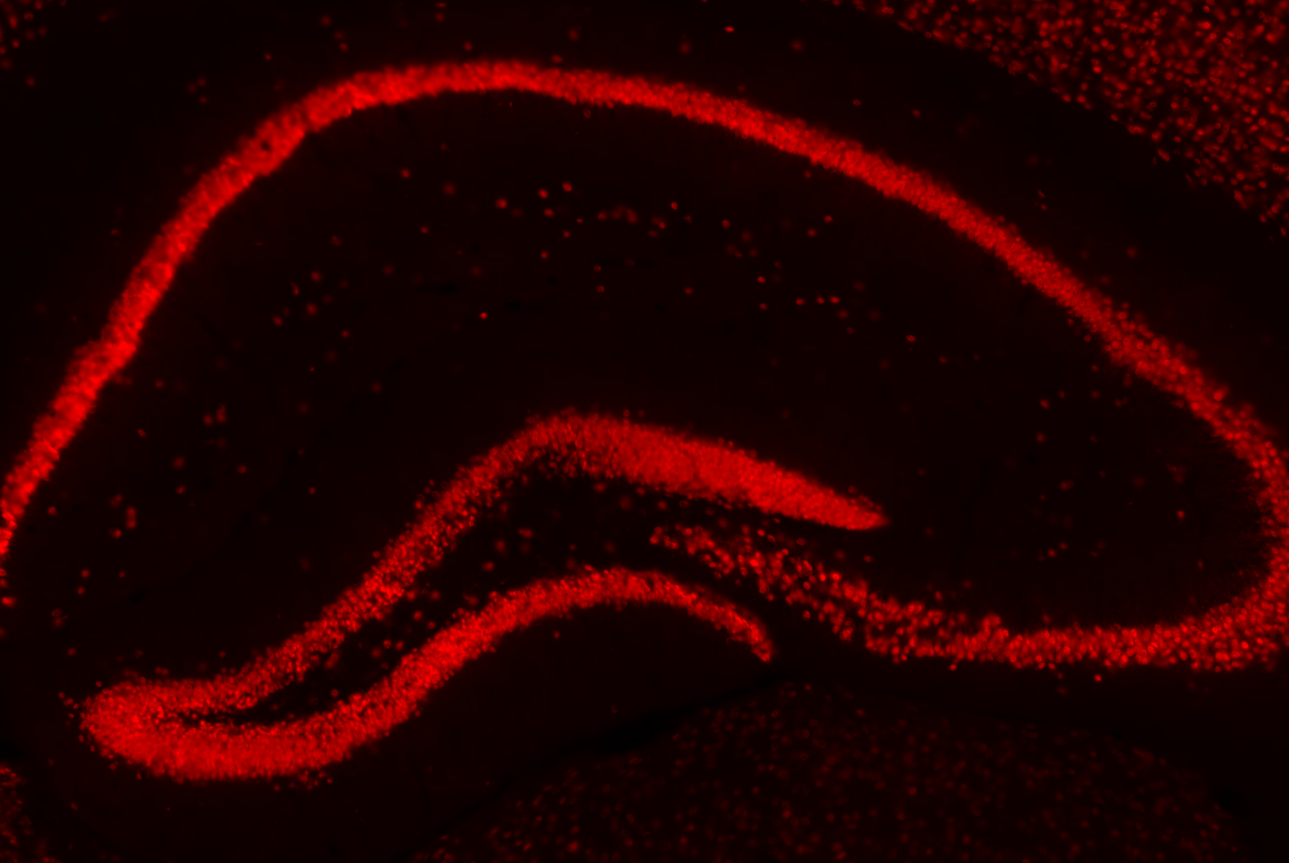

Supplement: Supplementary file 5 — Source data Fig. 2 [file 44321_2024_92_MOESM5_ESM.zip › Figure 2/2C/MPSIIIA saline 3 NeuN.tif]

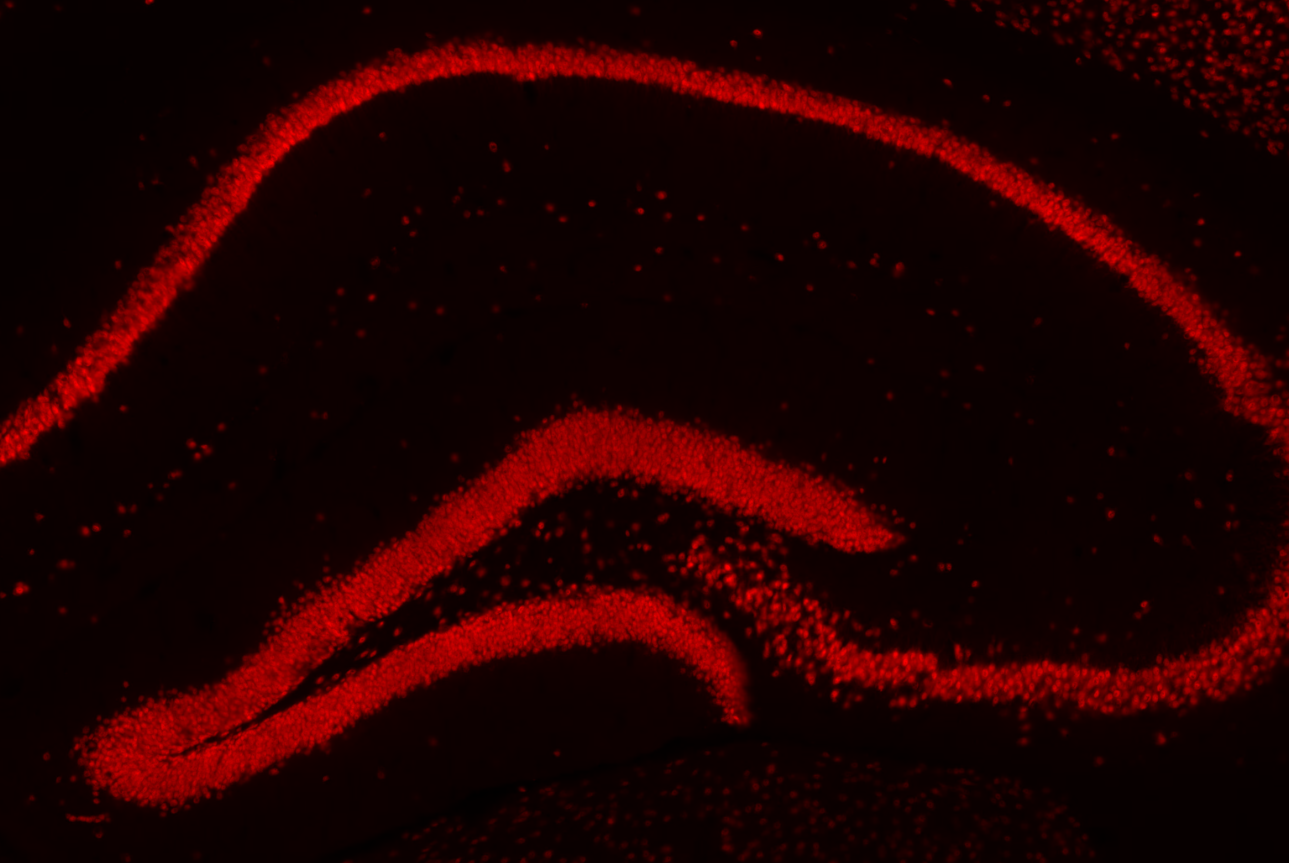

Supplement: Supplementary file 5 — Source data Fig. 2 [file 44321_2024_92_MOESM5_ESM.zip › Figure 2/2C/WT PolyIC 4 NeuN.tif]

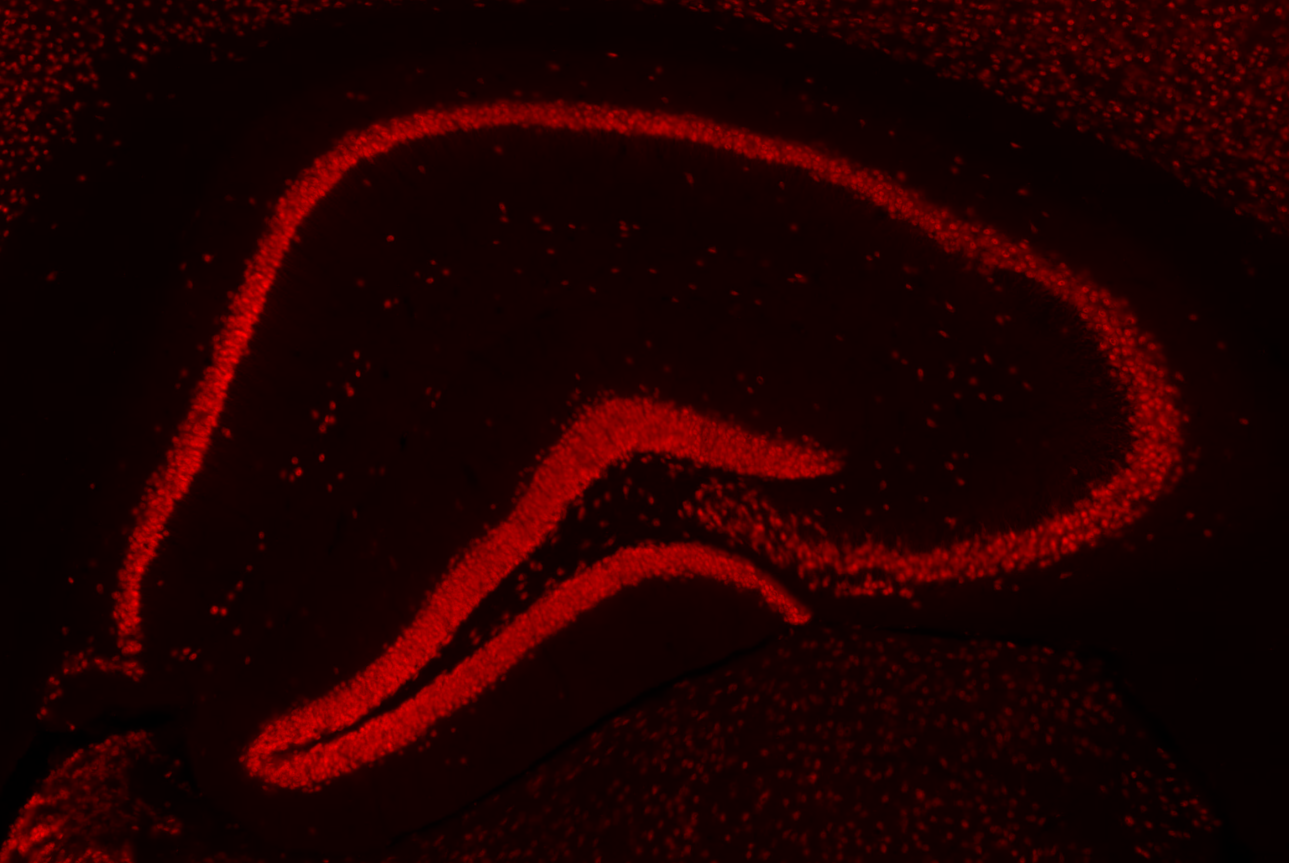

Supplement: Supplementary file 5 — Source data Fig. 2 [file 44321_2024_92_MOESM5_ESM.zip › Figure 2/2C/WT saline 2 NeuN.tif]

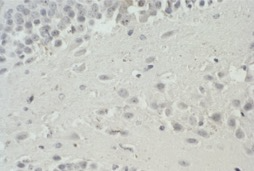

Supplement: Supplementary file 6 — Source data Fig. 3 [file 44321_2024_92_MOESM6_ESM.zip › Figure 3/3F/1.tiff]

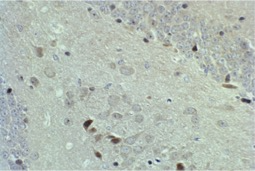

Supplement: Supplementary file 6 — Source data Fig. 3 [file 44321_2024_92_MOESM6_ESM.zip › Figure 3/3F/16.tiff]

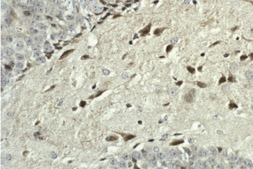

Supplement: Supplementary file 6 — Source data Fig. 3 [file 44321_2024_92_MOESM6_ESM.zip › Figure 3/3F/24.tiff]

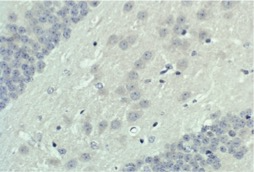

Supplement: Supplementary file 6 — Source data Fig. 3 [file 44321_2024_92_MOESM6_ESM.zip › Figure 3/3F/8.tiff]

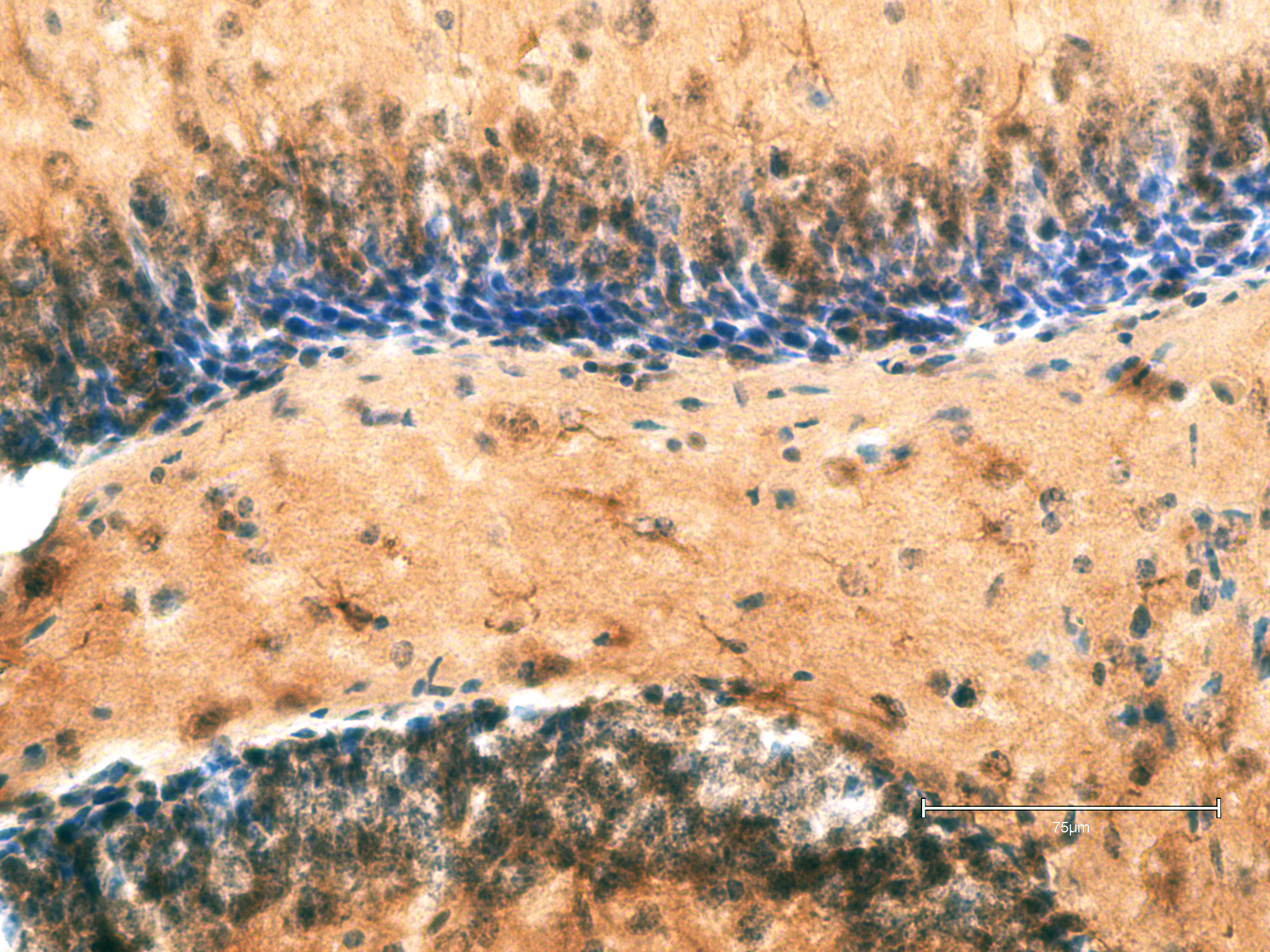

Supplement: Supplementary file 6 — Source data Fig. 3 [file 44321_2024_92_MOESM6_ESM.zip › Figure 3/3M/474 2nd 40x_0037 WT SALINE red-40 B+20, con+20.tif]

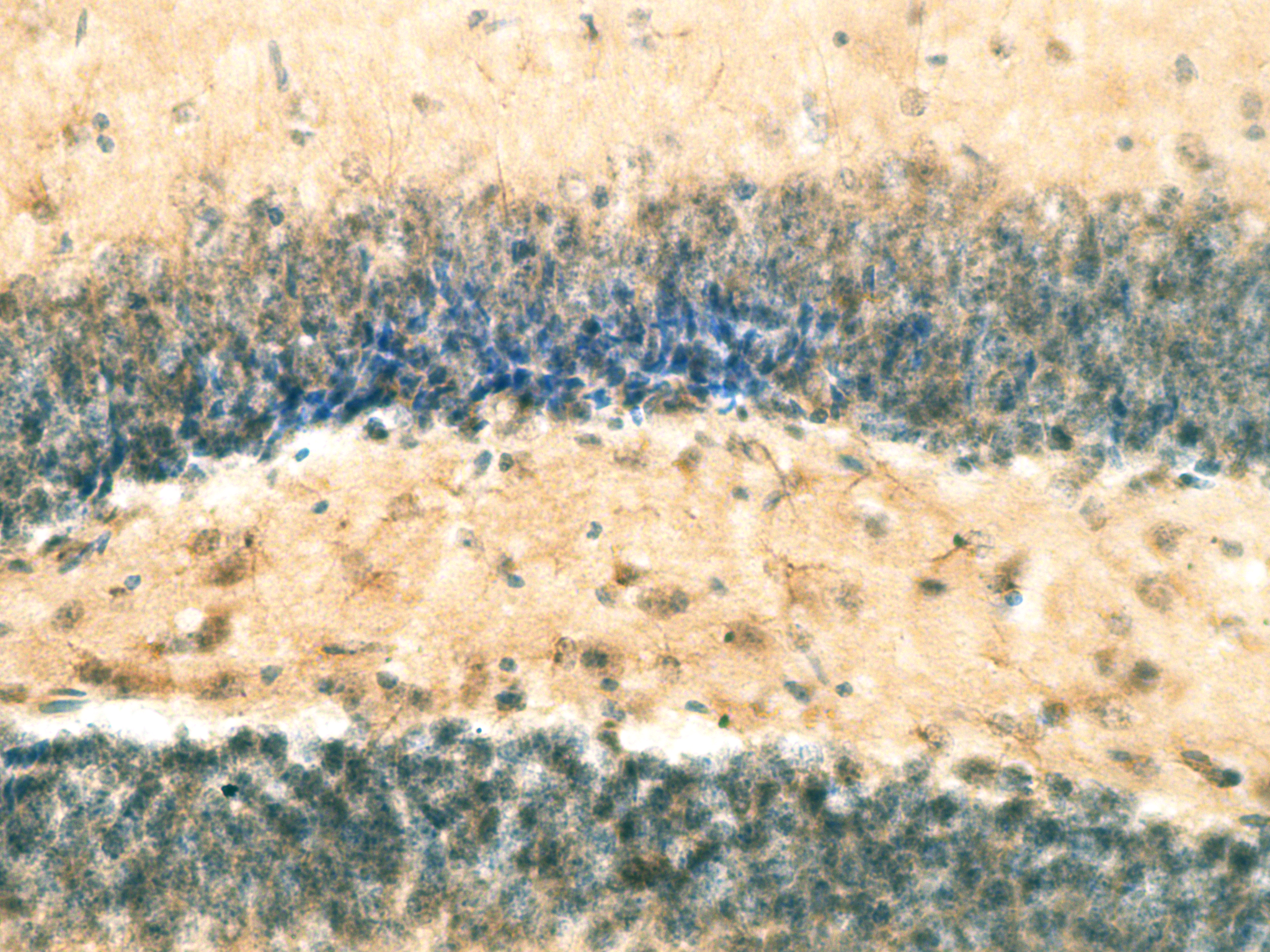

Supplement: Supplementary file 6 — Source data Fig. 3 [file 44321_2024_92_MOESM6_ESM.zip › Figure 3/3M/476 2nd 40x_0032_RGB Trans MPSIIIA Saline REd-40 B+20Con+20.tif]

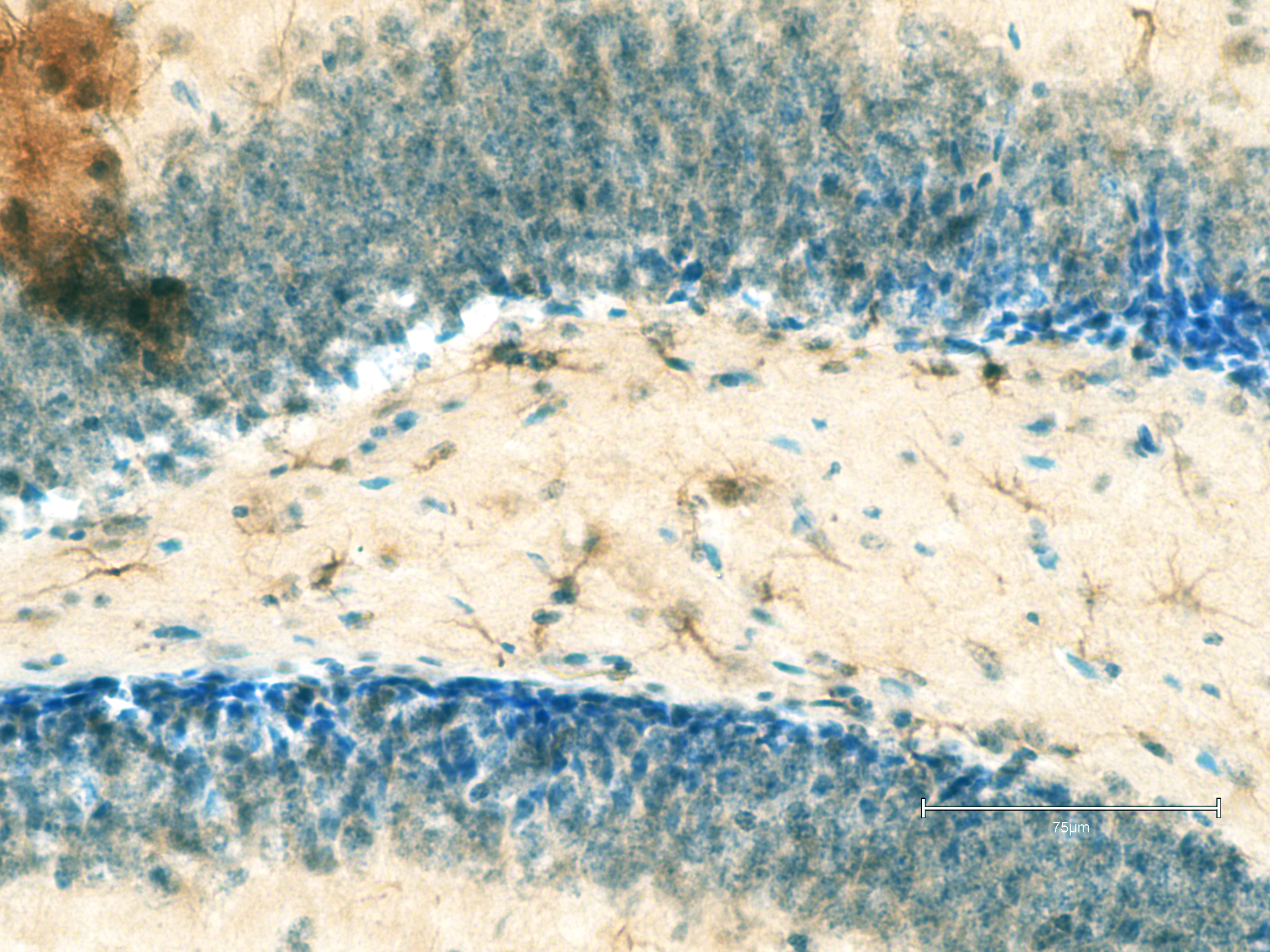

Supplement: Supplementary file 6 — Source data Fig. 3 [file 44321_2024_92_MOESM6_ESM.zip › Figure 3/3M/478 2nd 40x_0028 MPSIIIA PIC red-40,B+20,Con+20.tif]

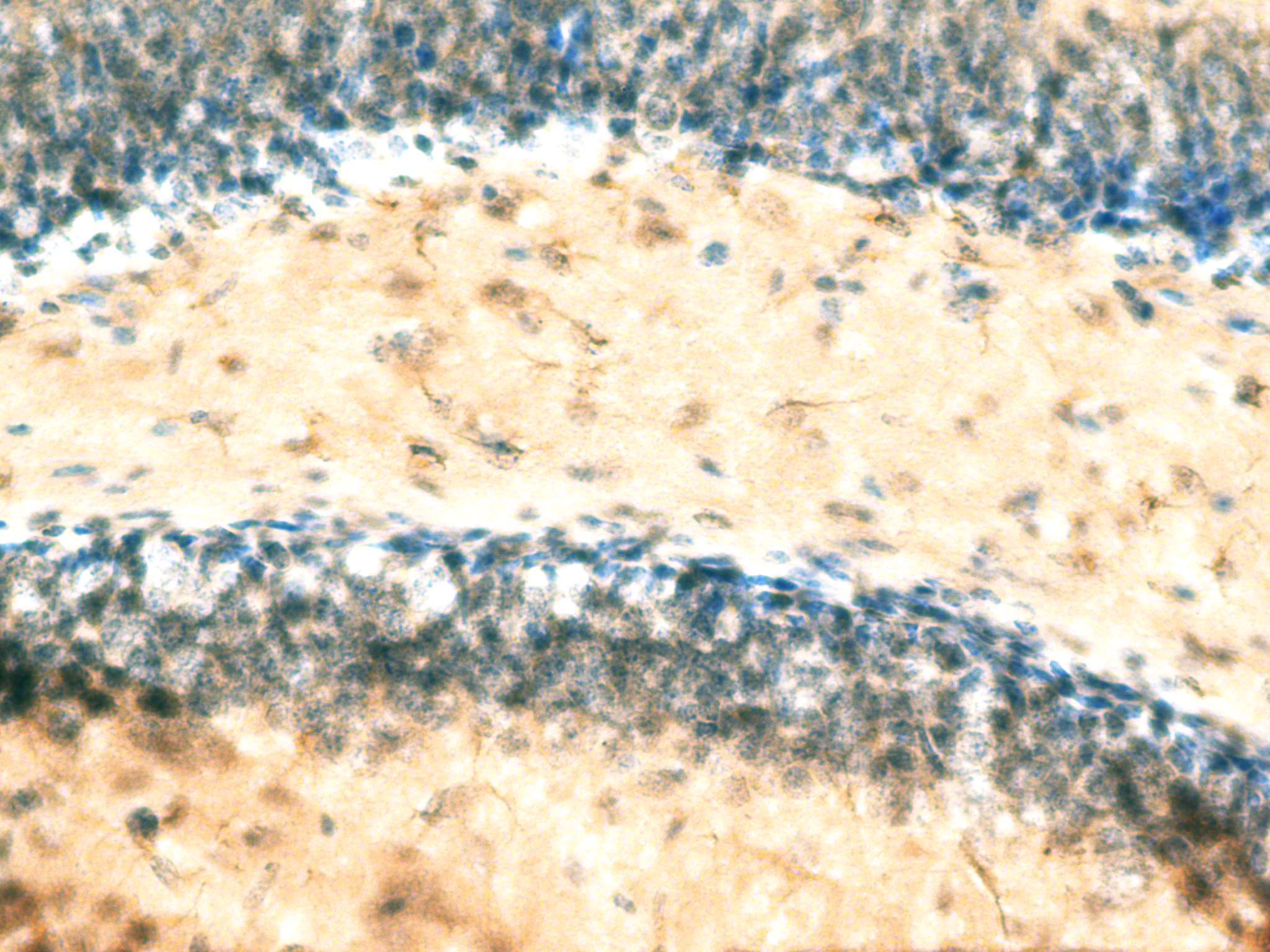

Supplement: Supplementary file 6 — Source data Fig. 3 [file 44321_2024_92_MOESM6_ESM.zip › Figure 3/3M/895 2nd 40x_0035_RGB Trans WT PIC red-40, B+20,Con+20.tif]

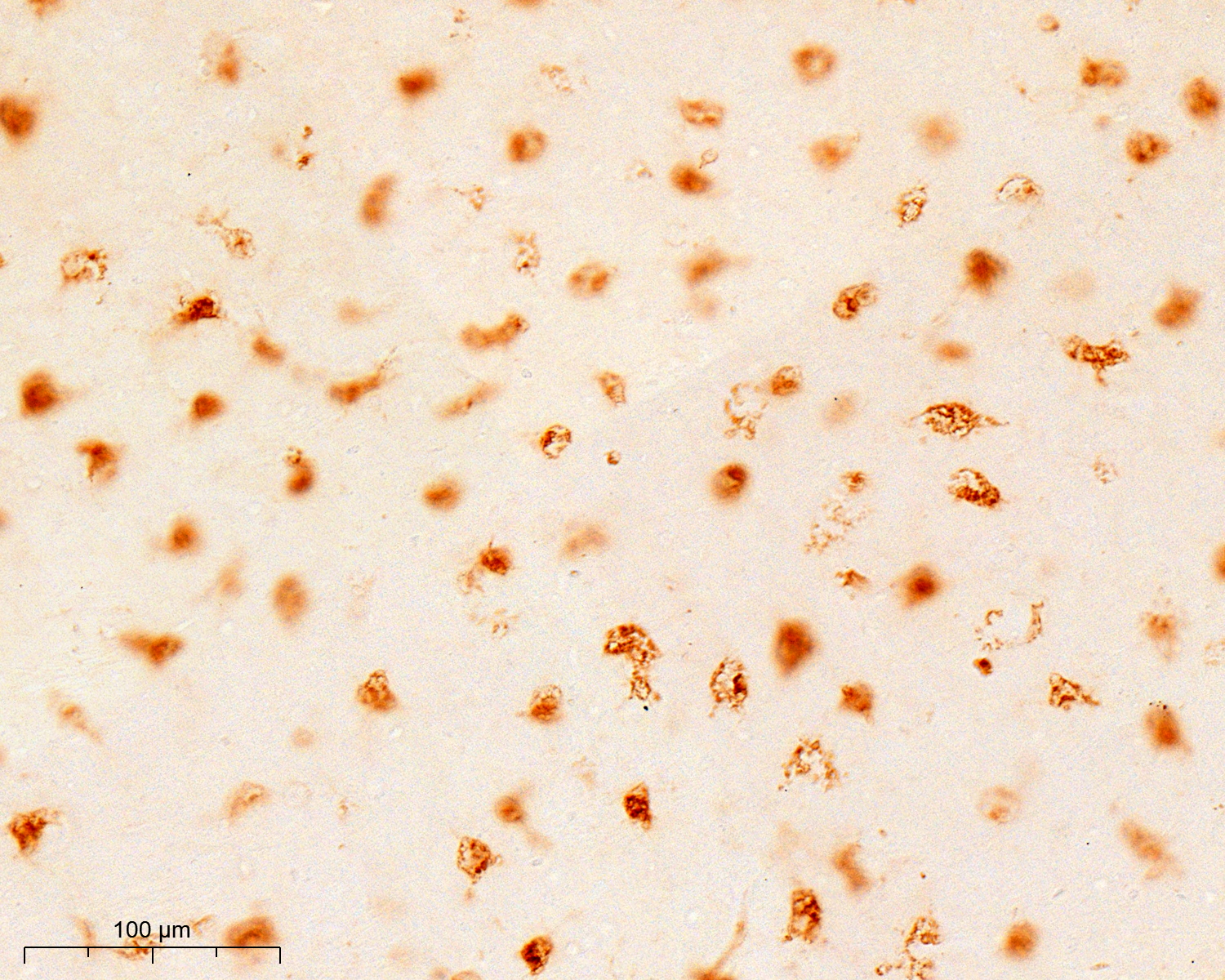

Supplement: Supplementary file 9 — Source data Fig. 6 [file 44321_2024_92_MOESM9_ESM.zip › Figure 6/6A/MPS PIC_Amygdala_ILB4_40.0x.jpg]

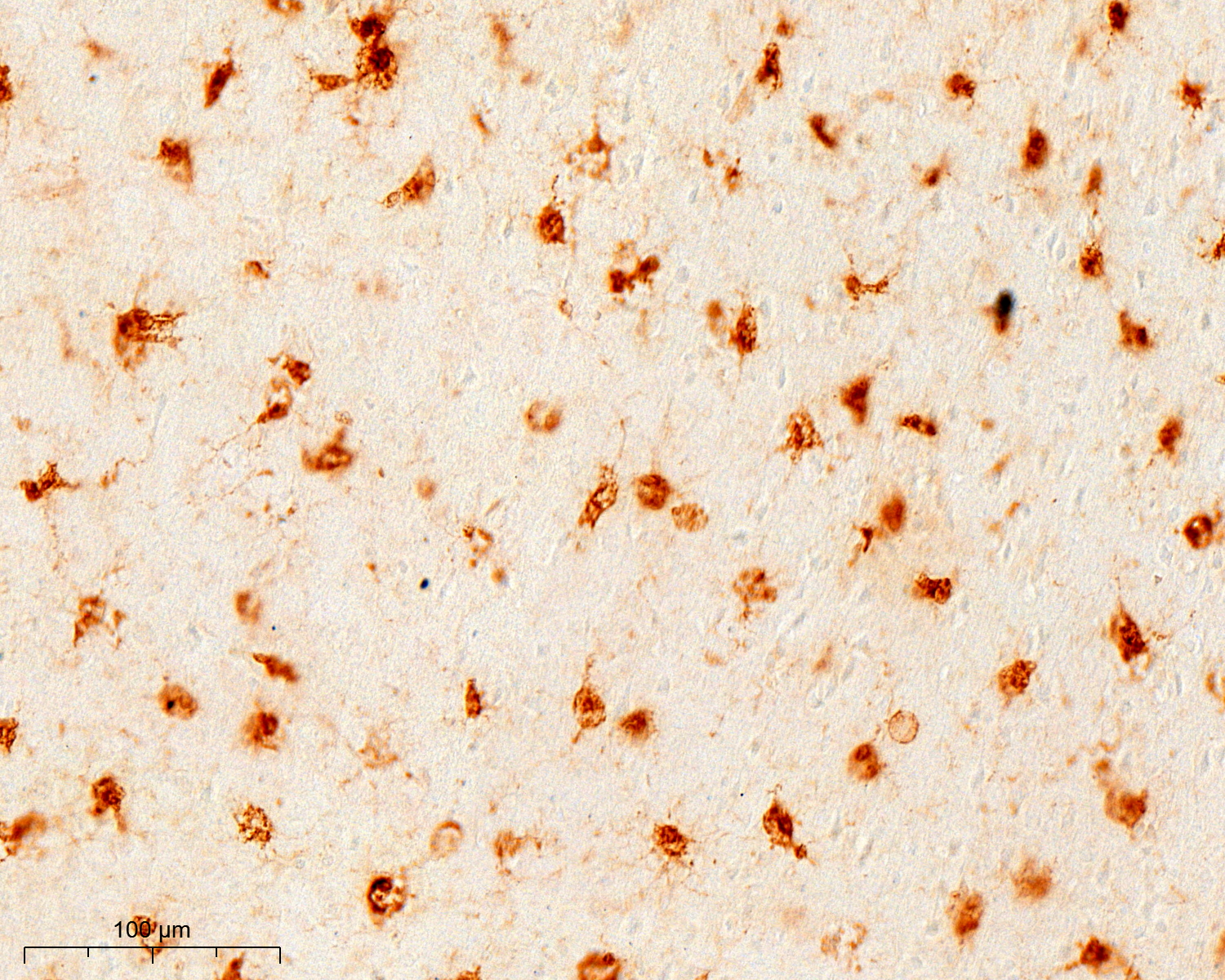

Supplement: Supplementary file 9 — Source data Fig. 6 [file 44321_2024_92_MOESM9_ESM.zip › Figure 6/6A/MPS PIC_Cortex_ILB4_40.0x.jpg]

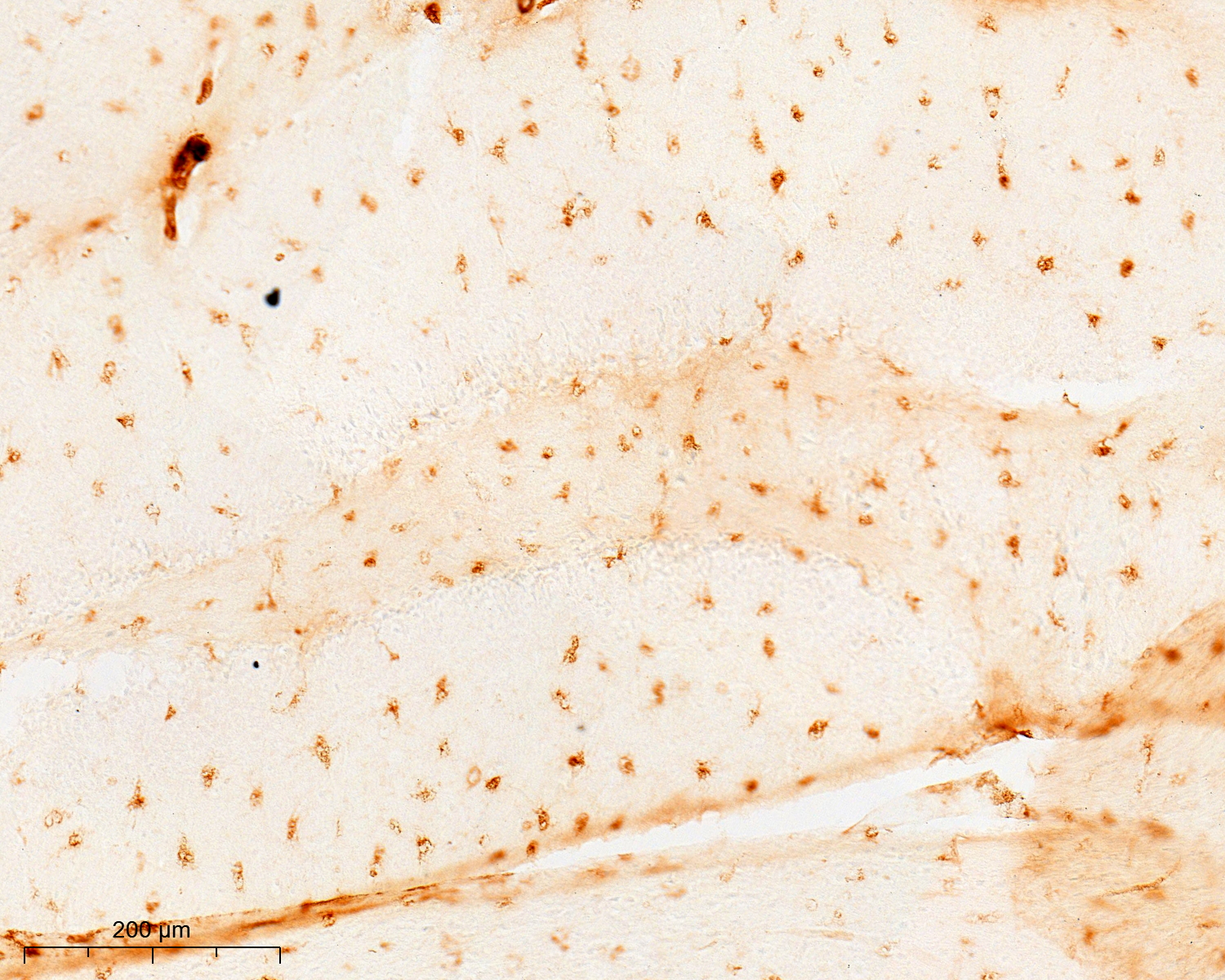

Supplement: Supplementary file 9 — Source data Fig. 6 [file 44321_2024_92_MOESM9_ESM.zip › Figure 6/6A/MPS PIC_Hippo_ILB4_20.0x.jpg]

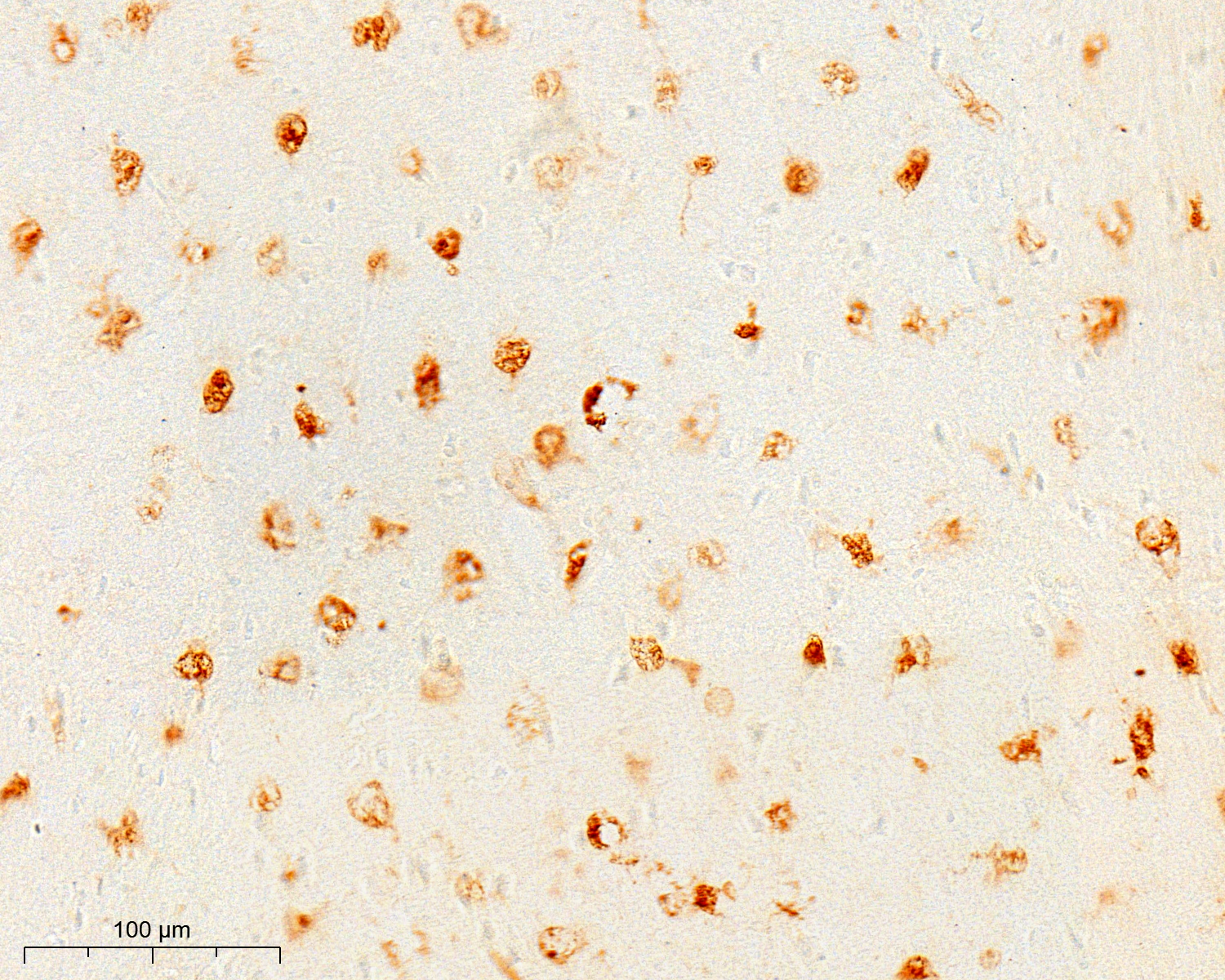

Supplement: Supplementary file 9 — Source data Fig. 6 [file 44321_2024_92_MOESM9_ESM.zip › Figure 6/6A/MPS_Amygdala_ILB4_40.0x.jpg]

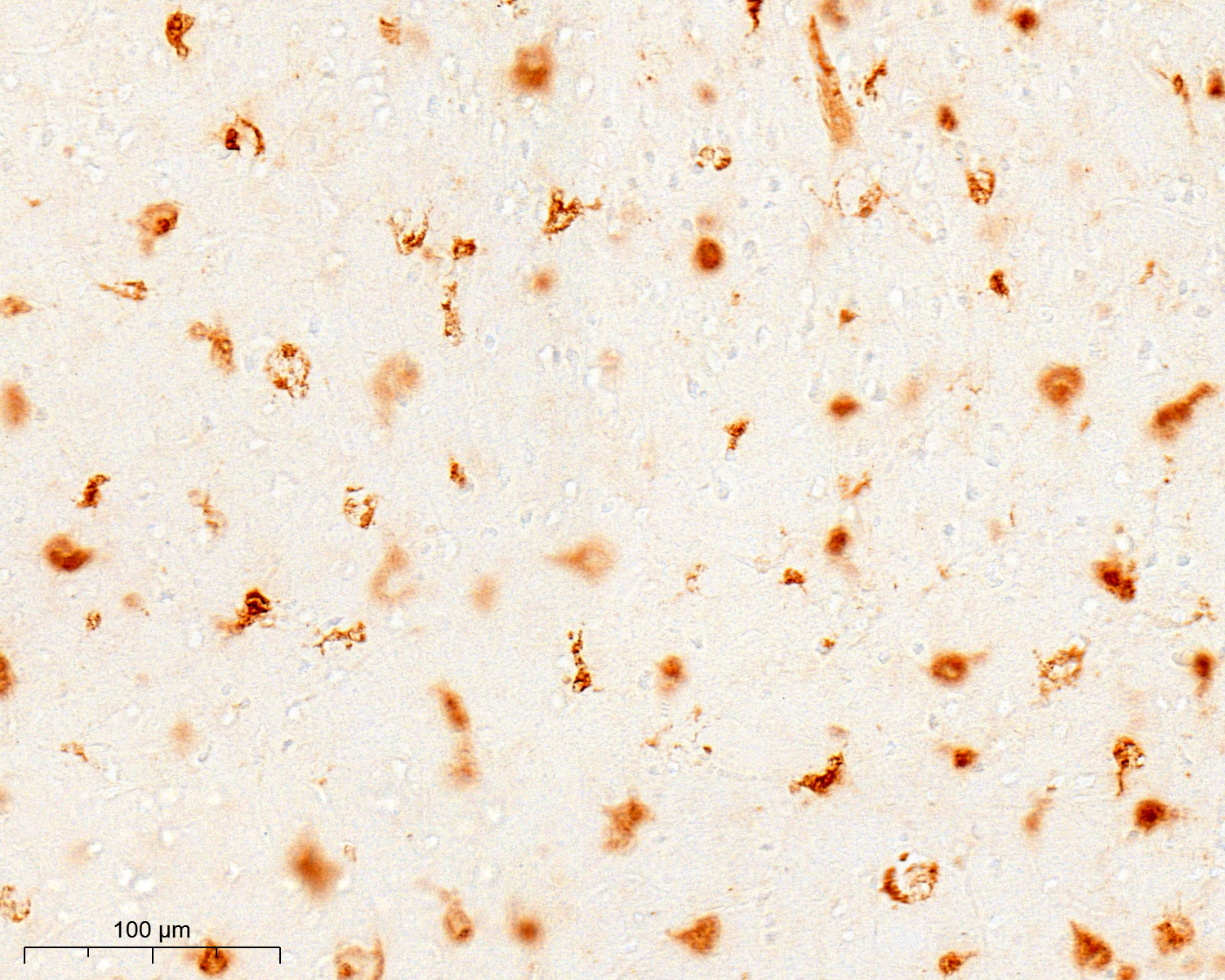

Supplement: Supplementary file 9 — Source data Fig. 6 [file 44321_2024_92_MOESM9_ESM.zip › Figure 6/6A/MPS_Cortex_ILB4_40.0x.jpg]

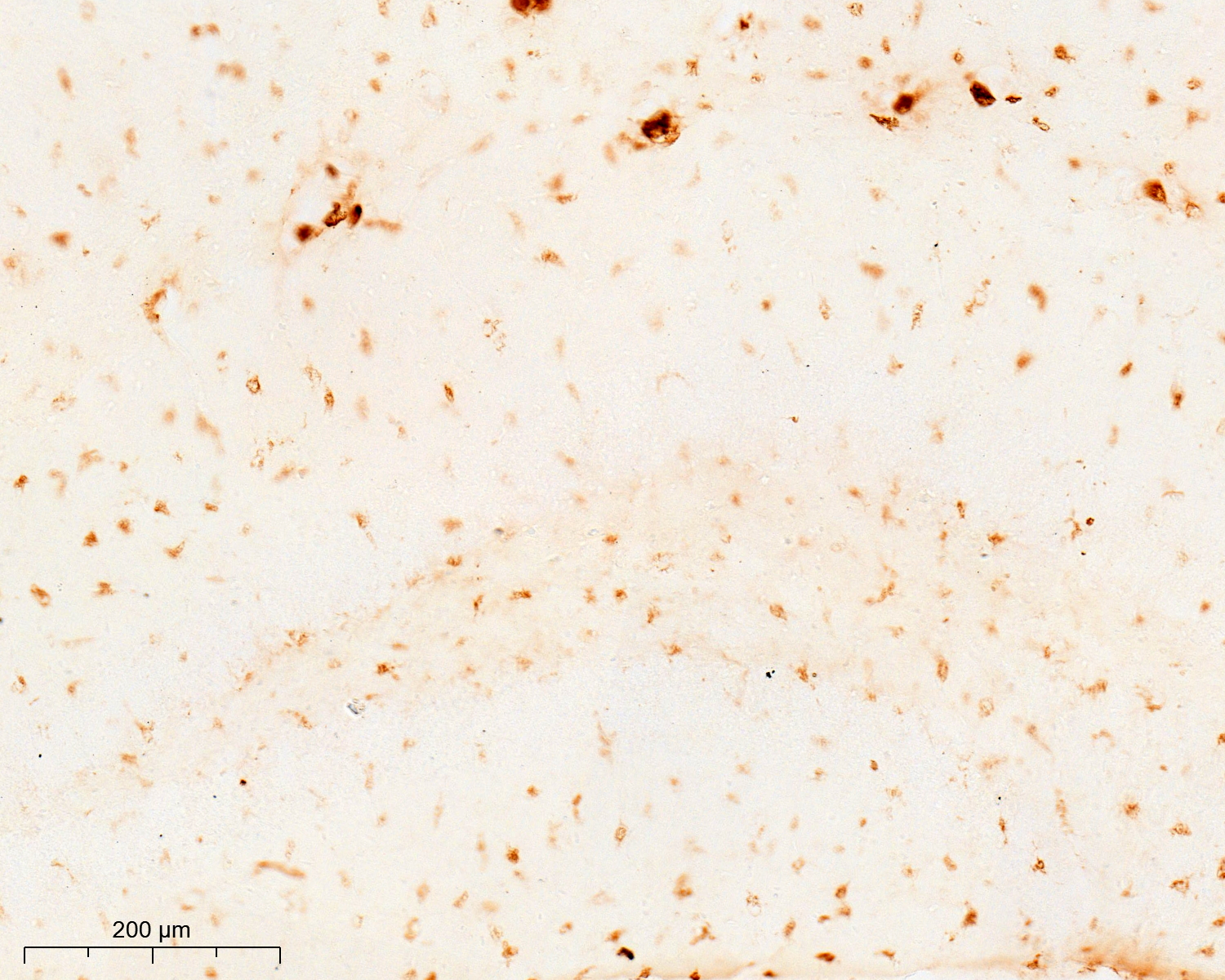

Supplement: Supplementary file 9 — Source data Fig. 6 [file 44321_2024_92_MOESM9_ESM.zip › Figure 6/6A/MPS_Hippo_ILB4_20.0x.jpg]

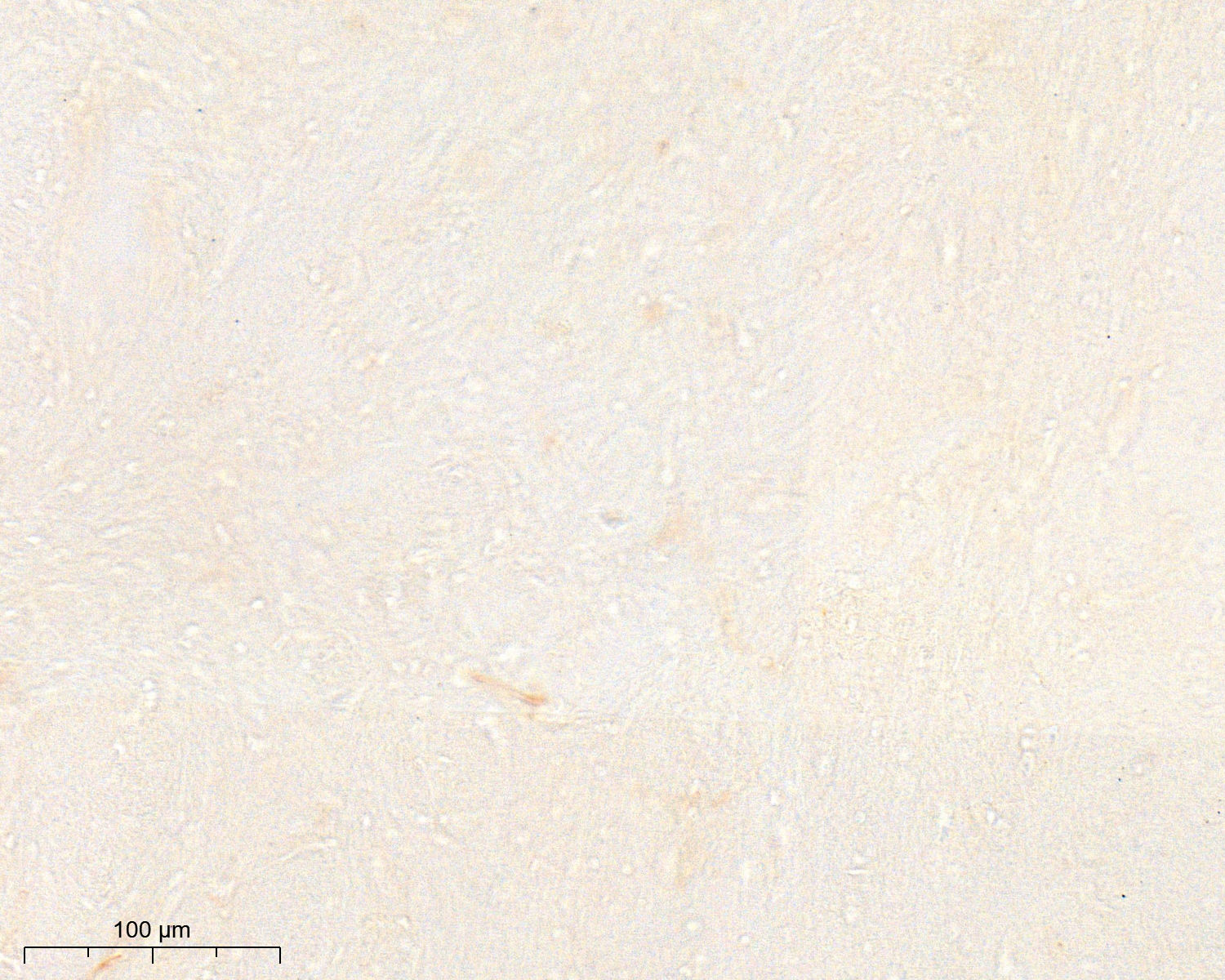

Supplement: Supplementary file 9 — Source data Fig. 6 [file 44321_2024_92_MOESM9_ESM.zip › Figure 6/6A/WT PIC_Amygdala_ILB4_40.0x.jpg]

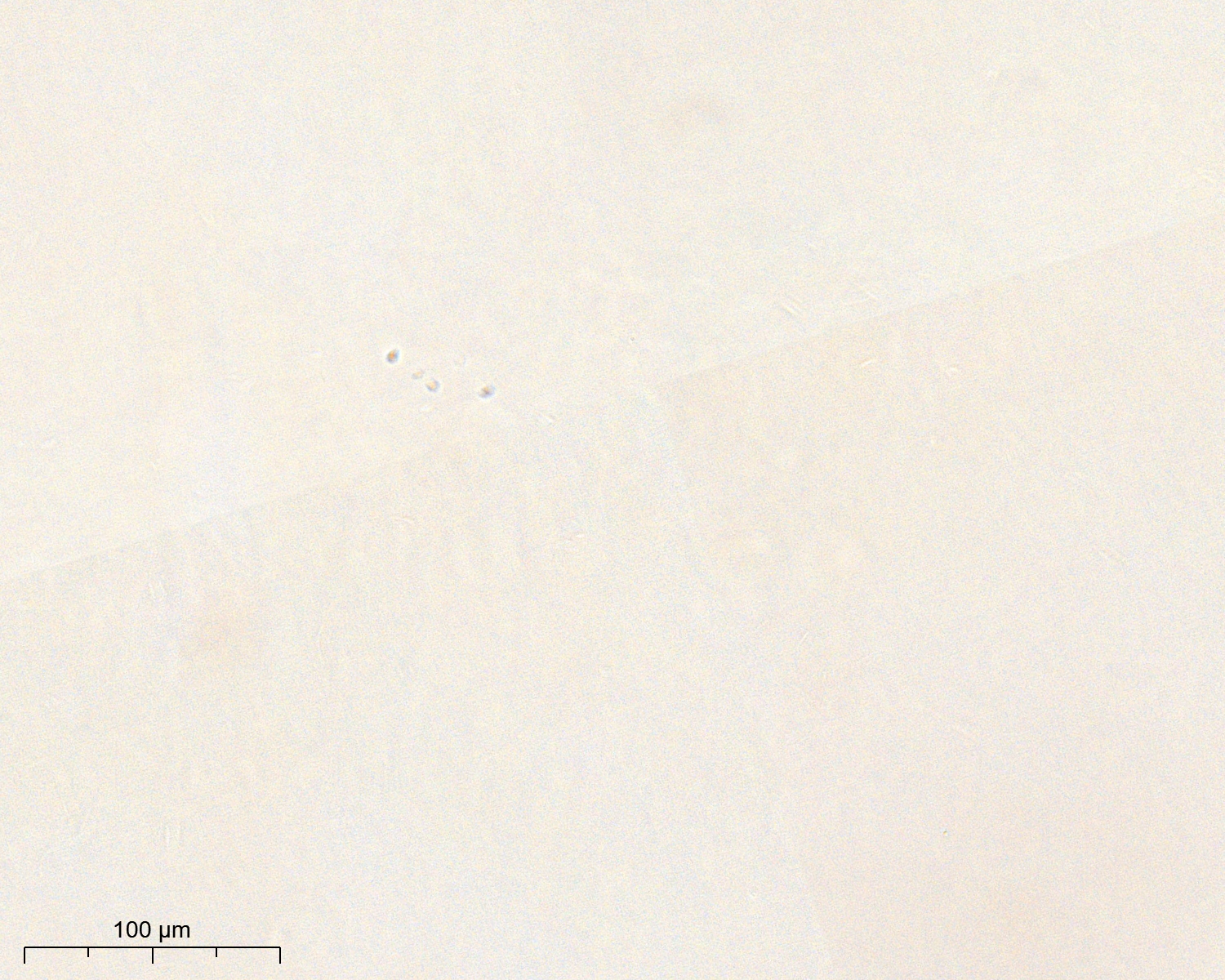

Supplement: Supplementary file 9 — Source data Fig. 6 [file 44321_2024_92_MOESM9_ESM.zip › Figure 6/6A/WT PIC_Cortex_ILB4_40.0x.jpg]

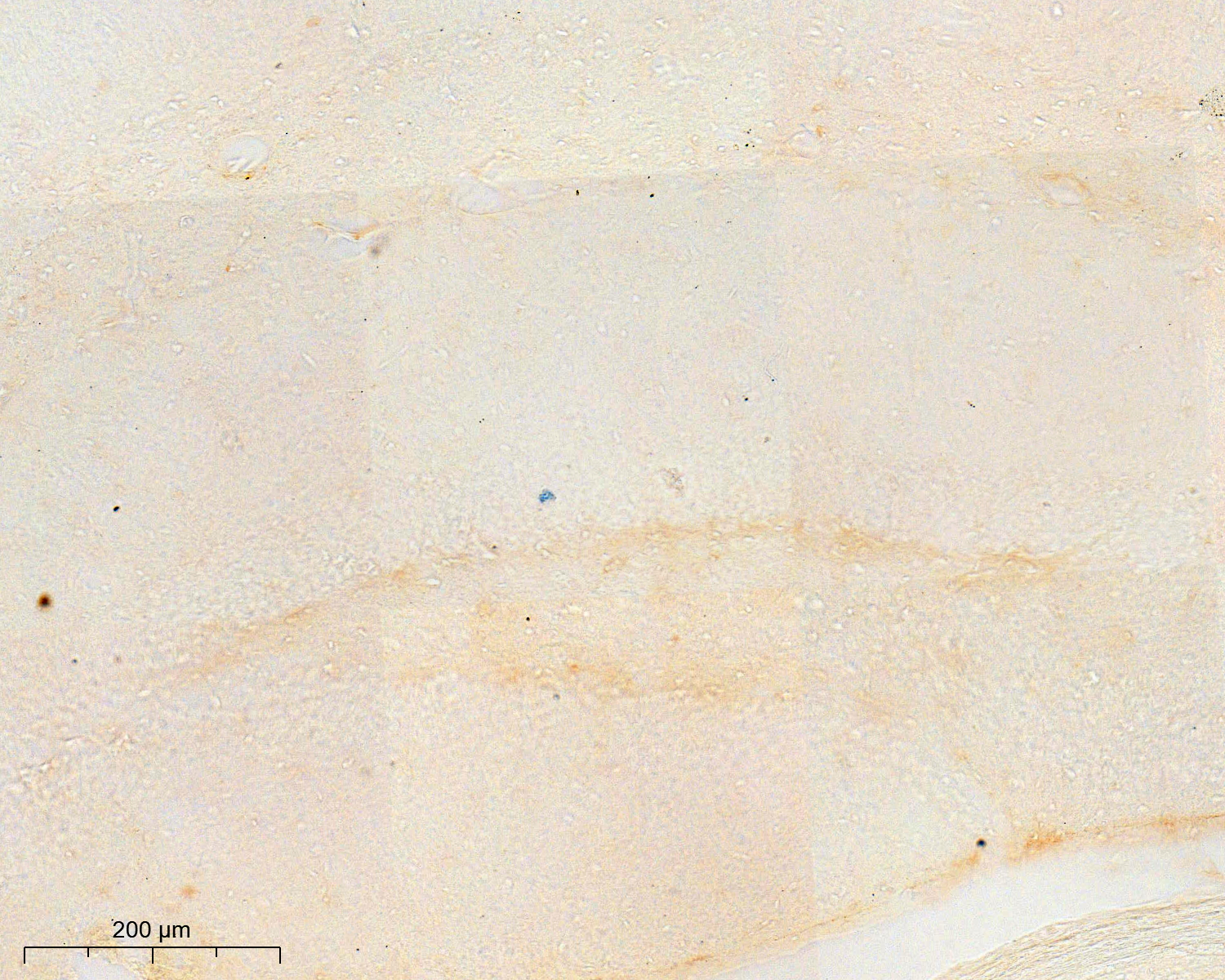

Supplement: Supplementary file 9 — Source data Fig. 6 [file 44321_2024_92_MOESM9_ESM.zip › Figure 6/6A/WT PIC_Hippo_ILB4_20.0x.jpg]

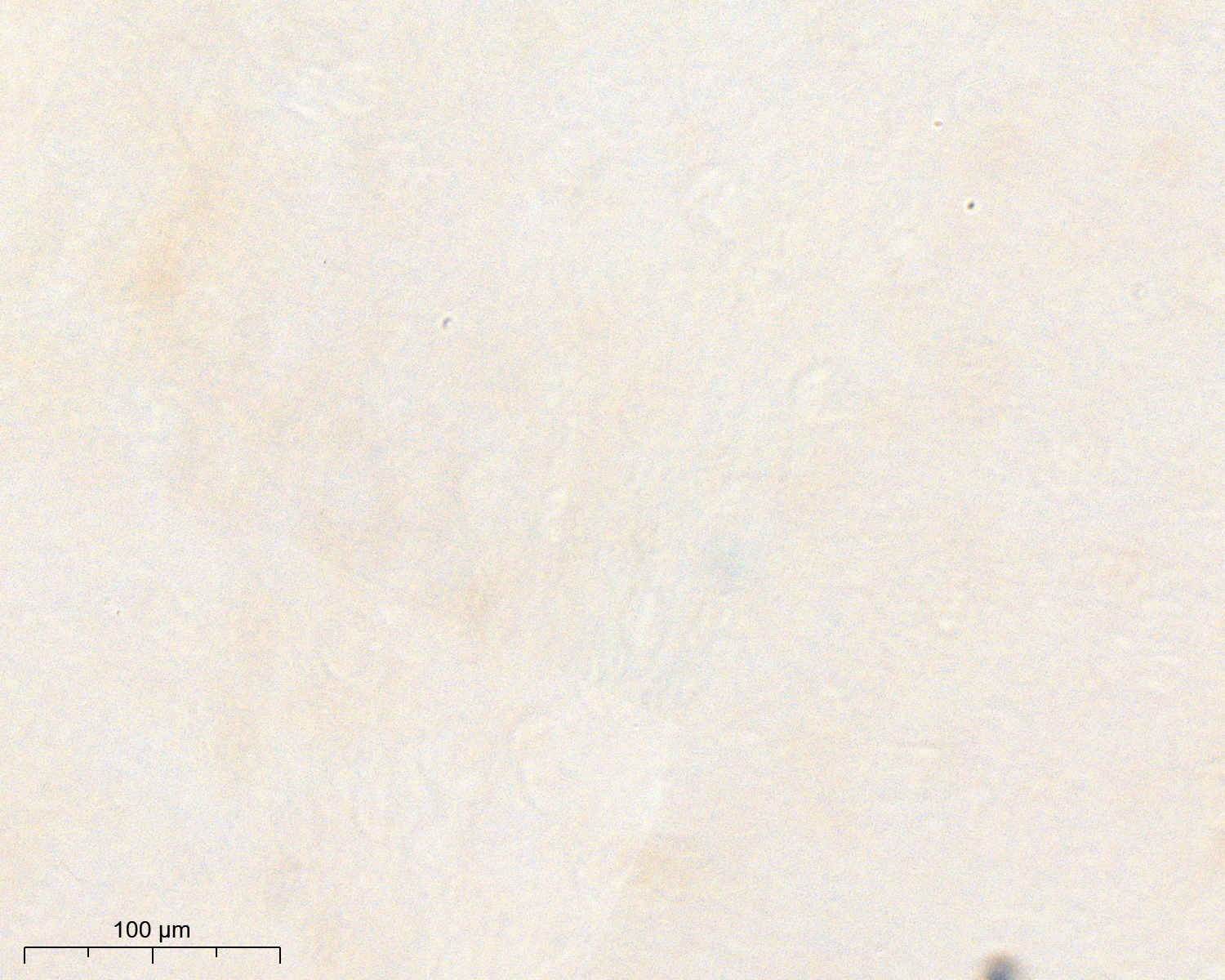

Supplement: Supplementary file 9 — Source data Fig. 6 [file 44321_2024_92_MOESM9_ESM.zip › Figure 6/6A/WT_Amygdala_ILB4_40.0x.jpg]

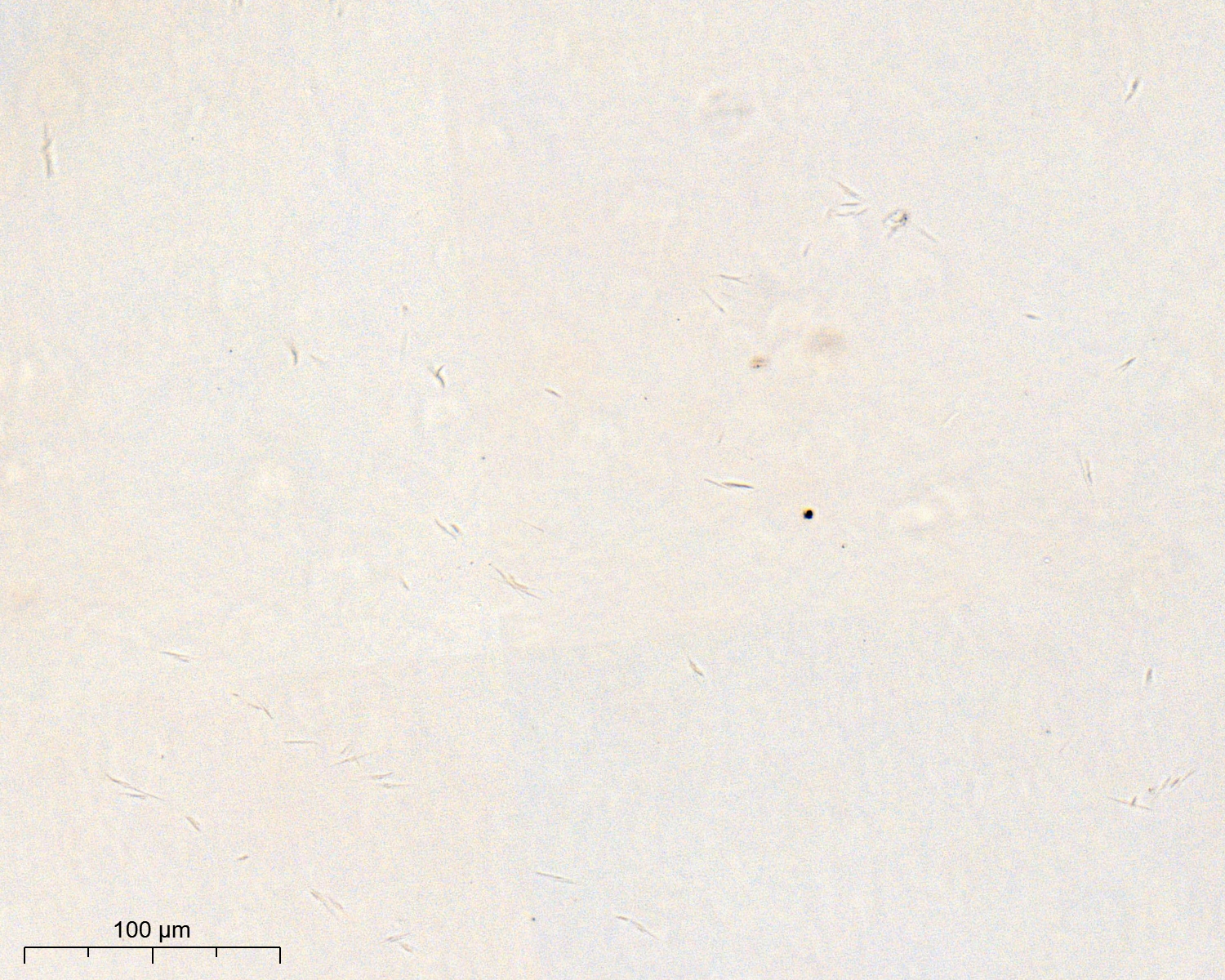

Supplement: Supplementary file 9 — Source data Fig. 6 [file 44321_2024_92_MOESM9_ESM.zip › Figure 6/6A/WT_Cortex_ILB4_40.0x.jpg]

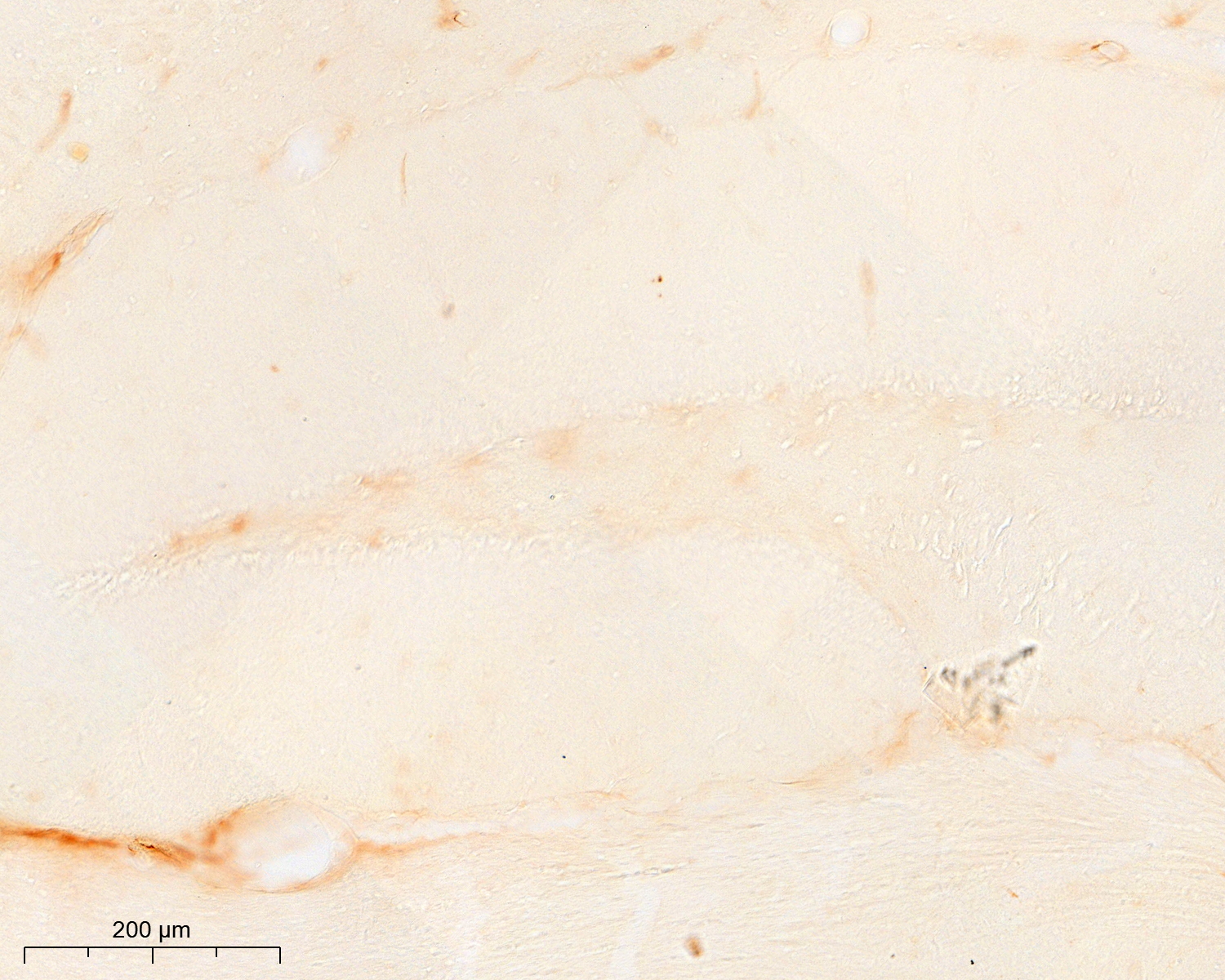

Supplement: Supplementary file 9 — Source data Fig. 6 [file 44321_2024_92_MOESM9_ESM.zip › Figure 6/6A/WT_Hippo_ILB4_20.0x.jpg]

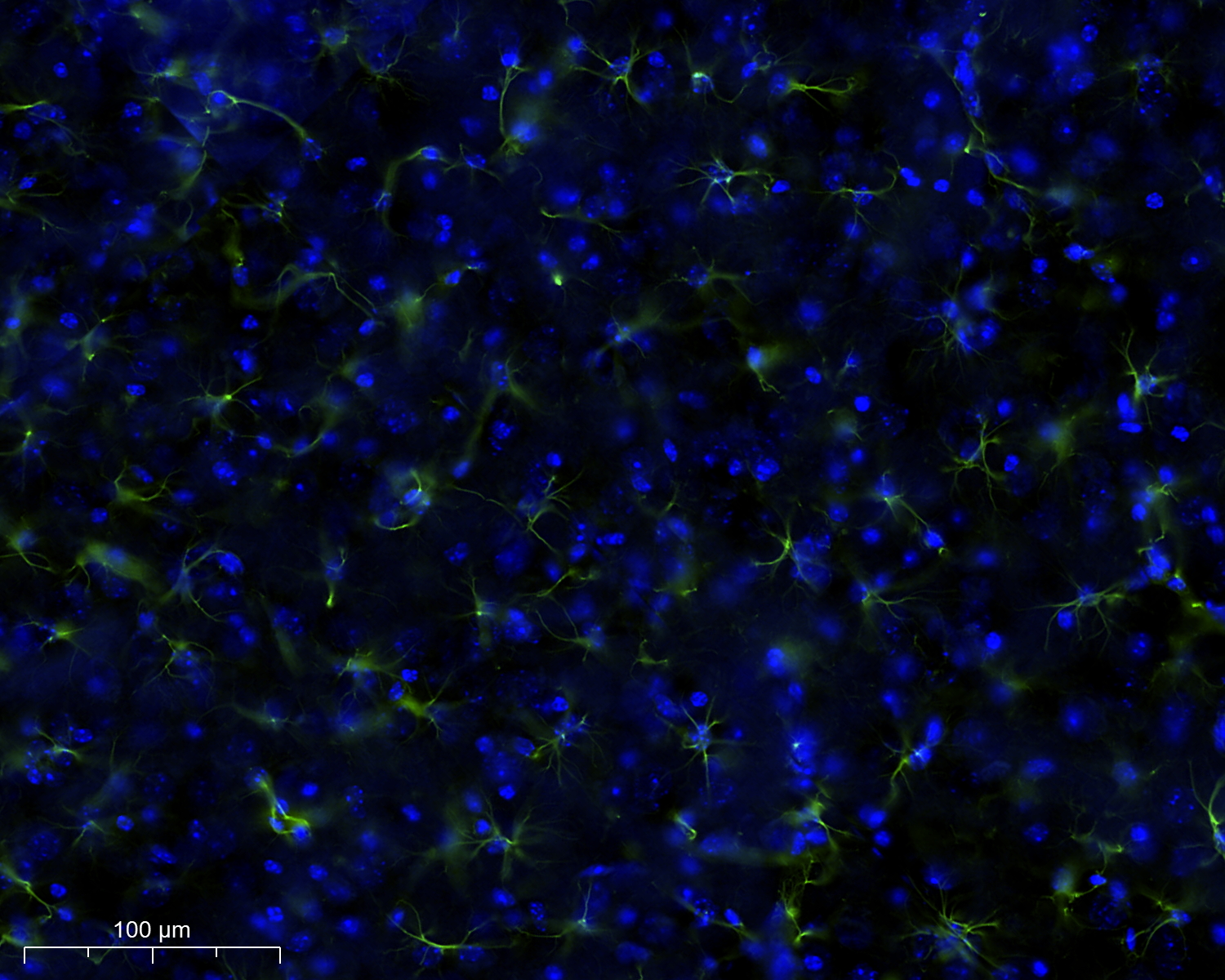

Supplement: Supplementary file 10 — Source data Fig. 7 [file 44321_2024_92_MOESM10_ESM.zip › Figure 7/7A/MPS PIC_Amy_GFAP_40.0x.jpg]

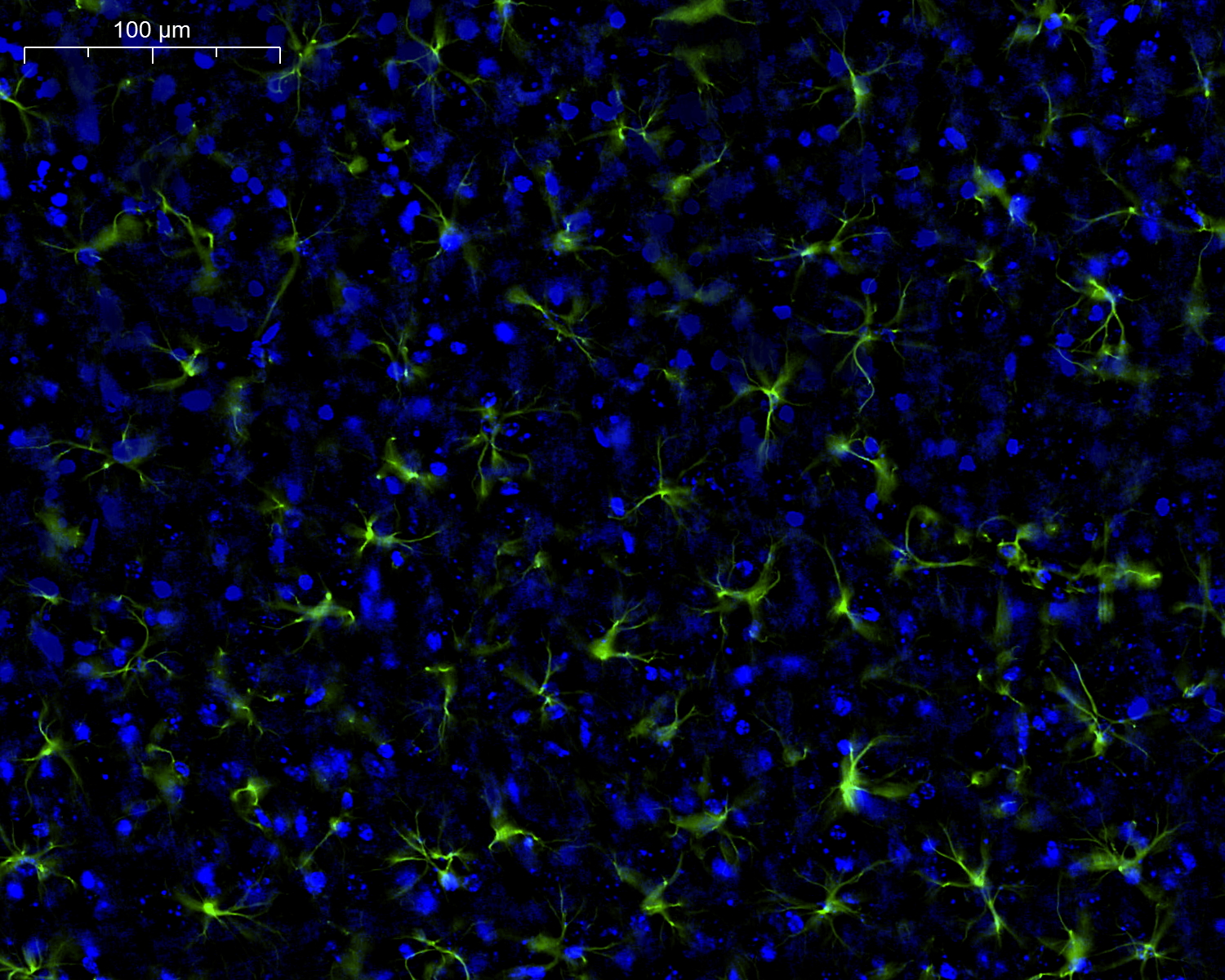

Supplement: Supplementary file 10 — Source data Fig. 7 [file 44321_2024_92_MOESM10_ESM.zip › Figure 7/7A/MPS PIC_Cortex_ GFAP_40.0x.jpg]

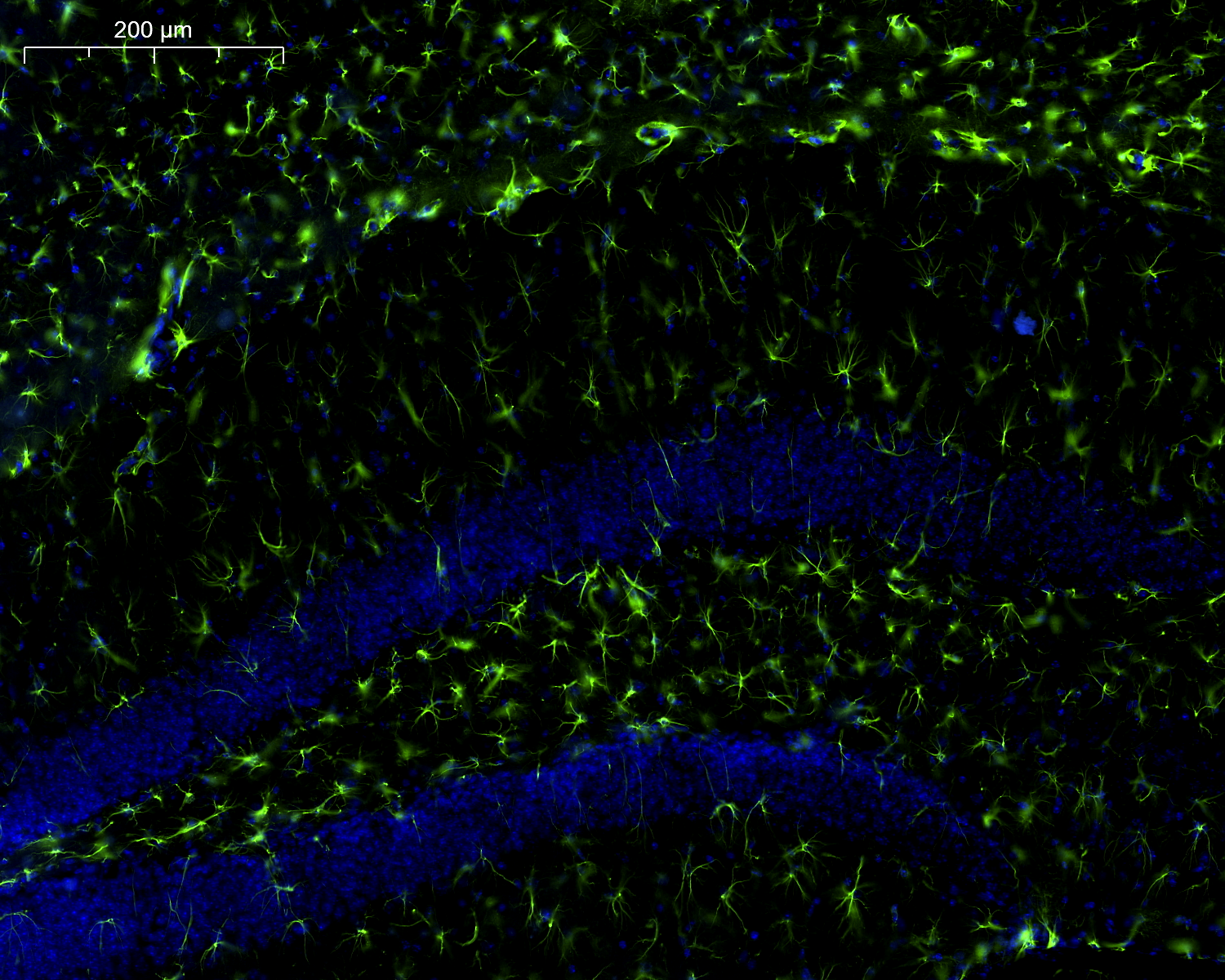

Supplement: Supplementary file 10 — Source data Fig. 7 [file 44321_2024_92_MOESM10_ESM.zip › Figure 7/7A/MPS PIC_Hippo_GFAP_20.0x.tif]

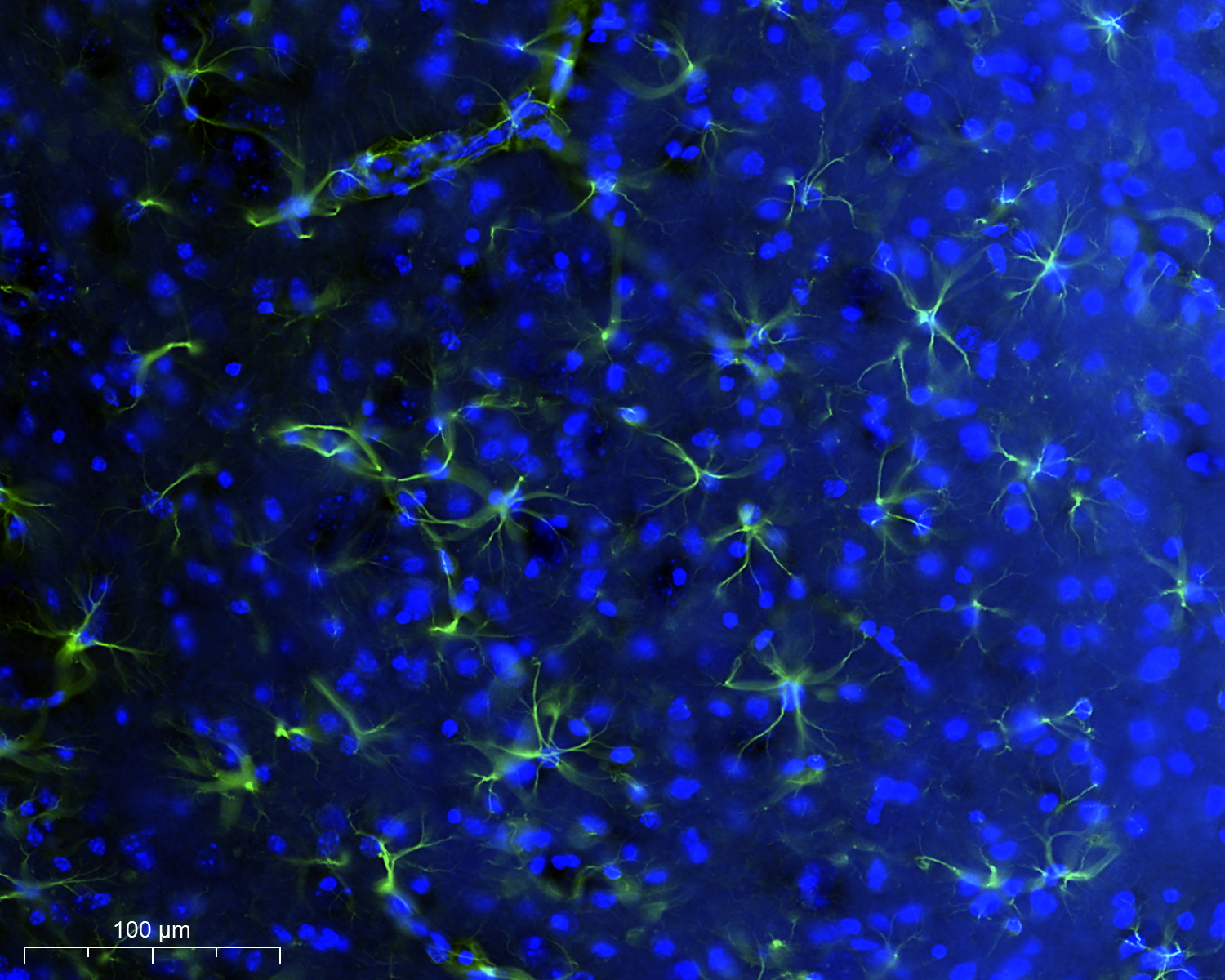

Supplement: Supplementary file 10 — Source data Fig. 7 [file 44321_2024_92_MOESM10_ESM.zip › Figure 7/7A/MPS_Amy_GFAP_40.0x.jpg]

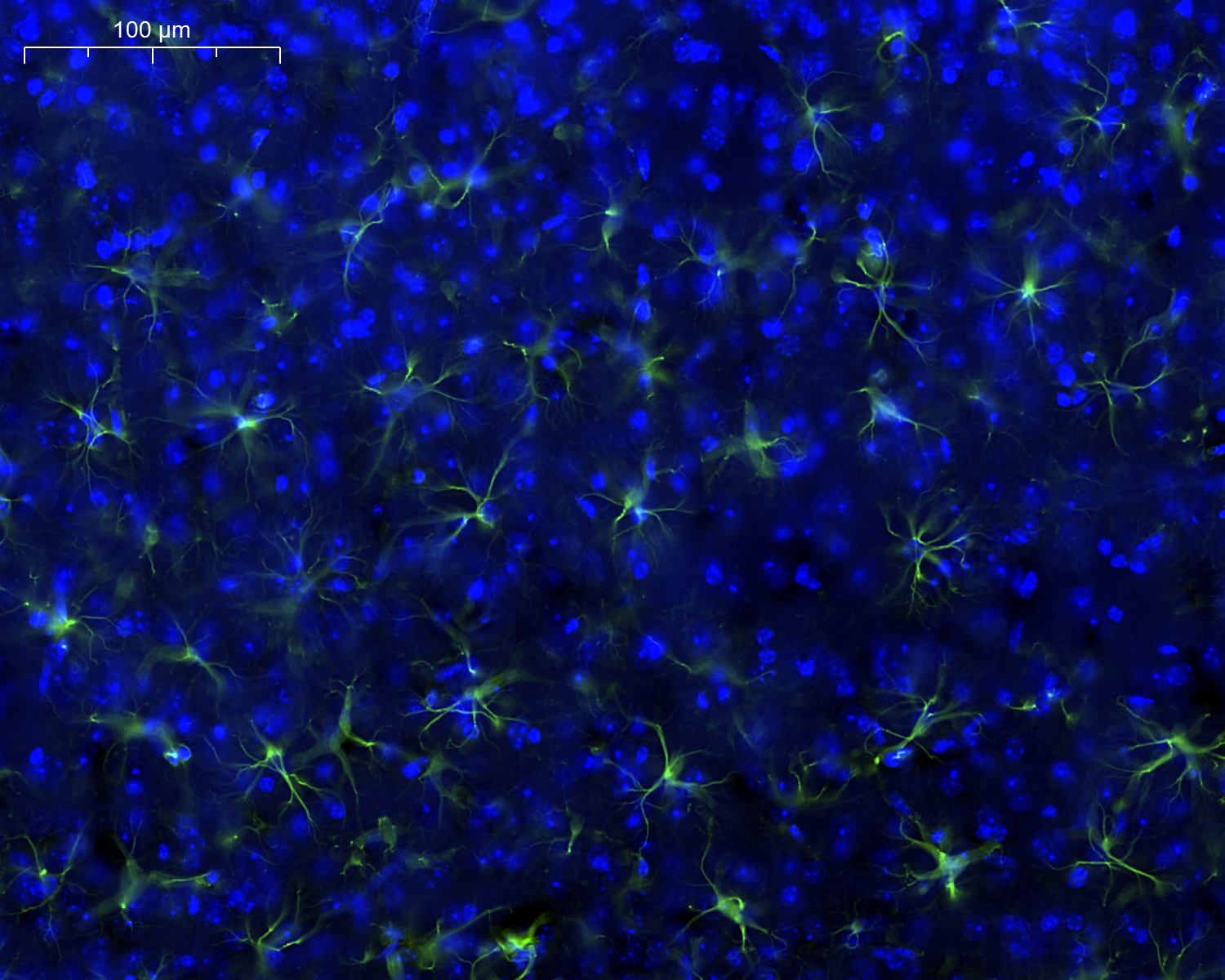

Supplement: Supplementary file 10 — Source data Fig. 7 [file 44321_2024_92_MOESM10_ESM.zip › Figure 7/7A/MPS_Cortex_GFAP_40x.jpg]

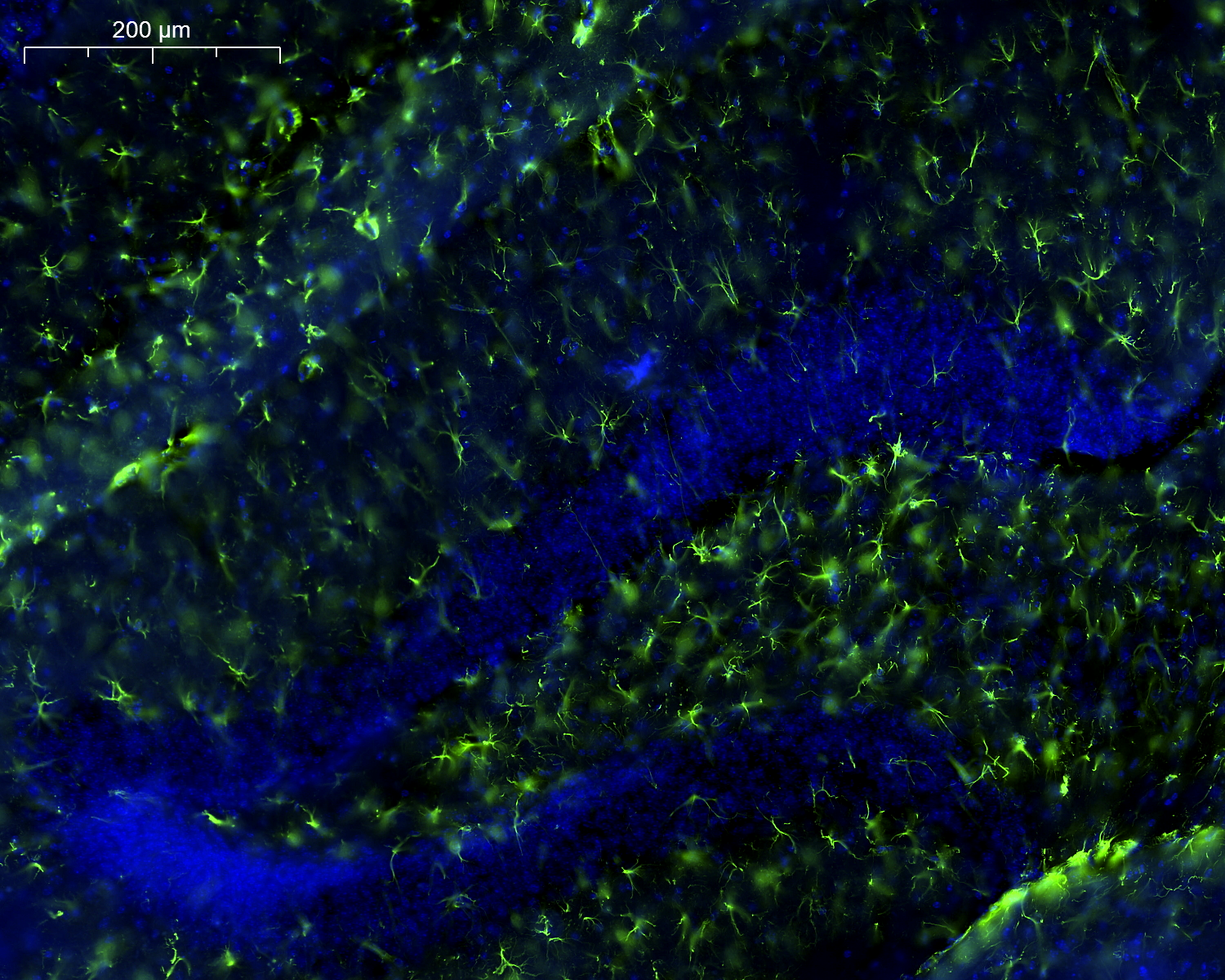

Supplement: Supplementary file 10 — Source data Fig. 7 [file 44321_2024_92_MOESM10_ESM.zip › Figure 7/7A/MPS_Hippo_GFAP_20.0x.tif]

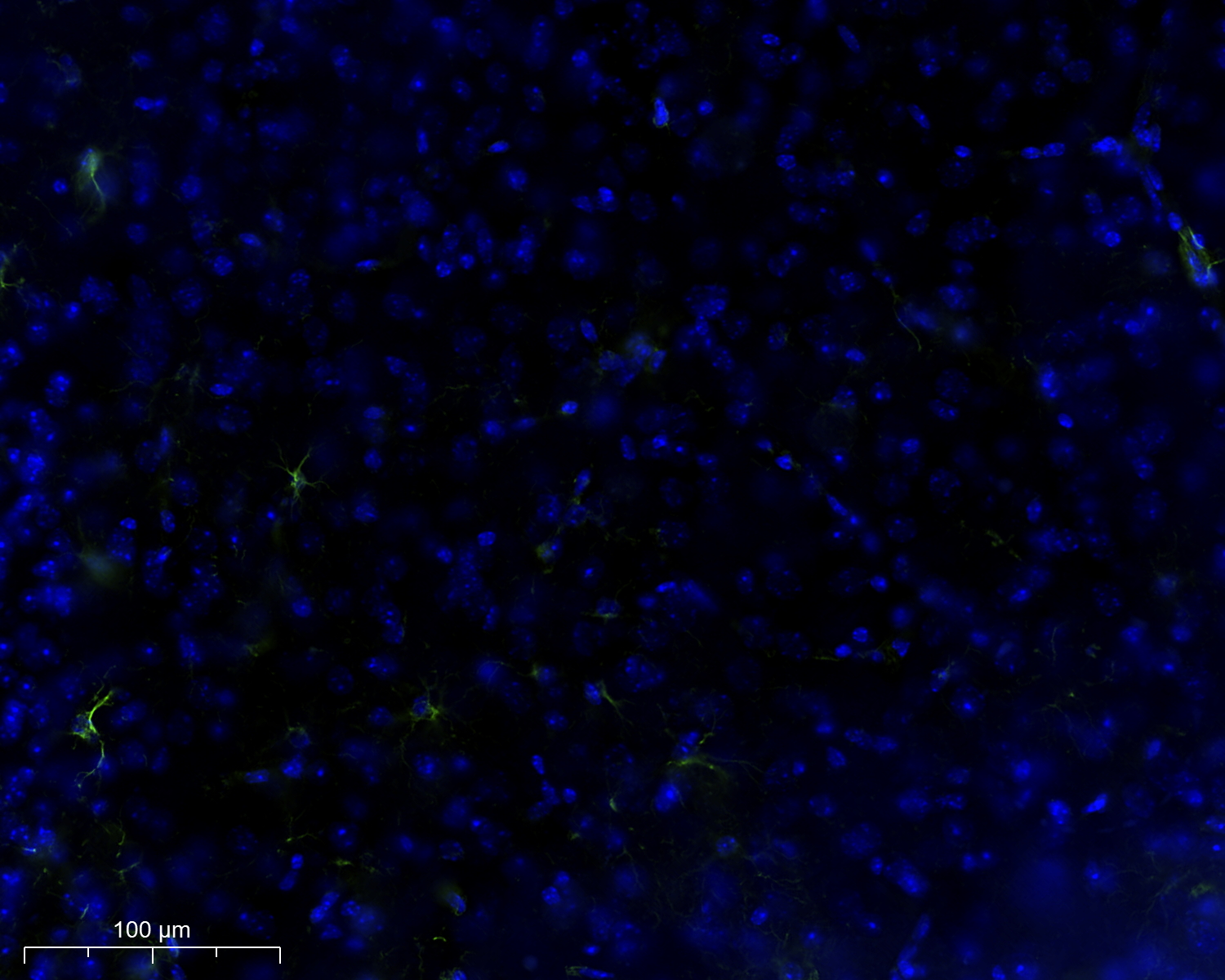

Supplement: Supplementary file 10 — Source data Fig. 7 [file 44321_2024_92_MOESM10_ESM.zip › Figure 7/7A/WT PIC_Amy_GFAP_40.0x.jpg]

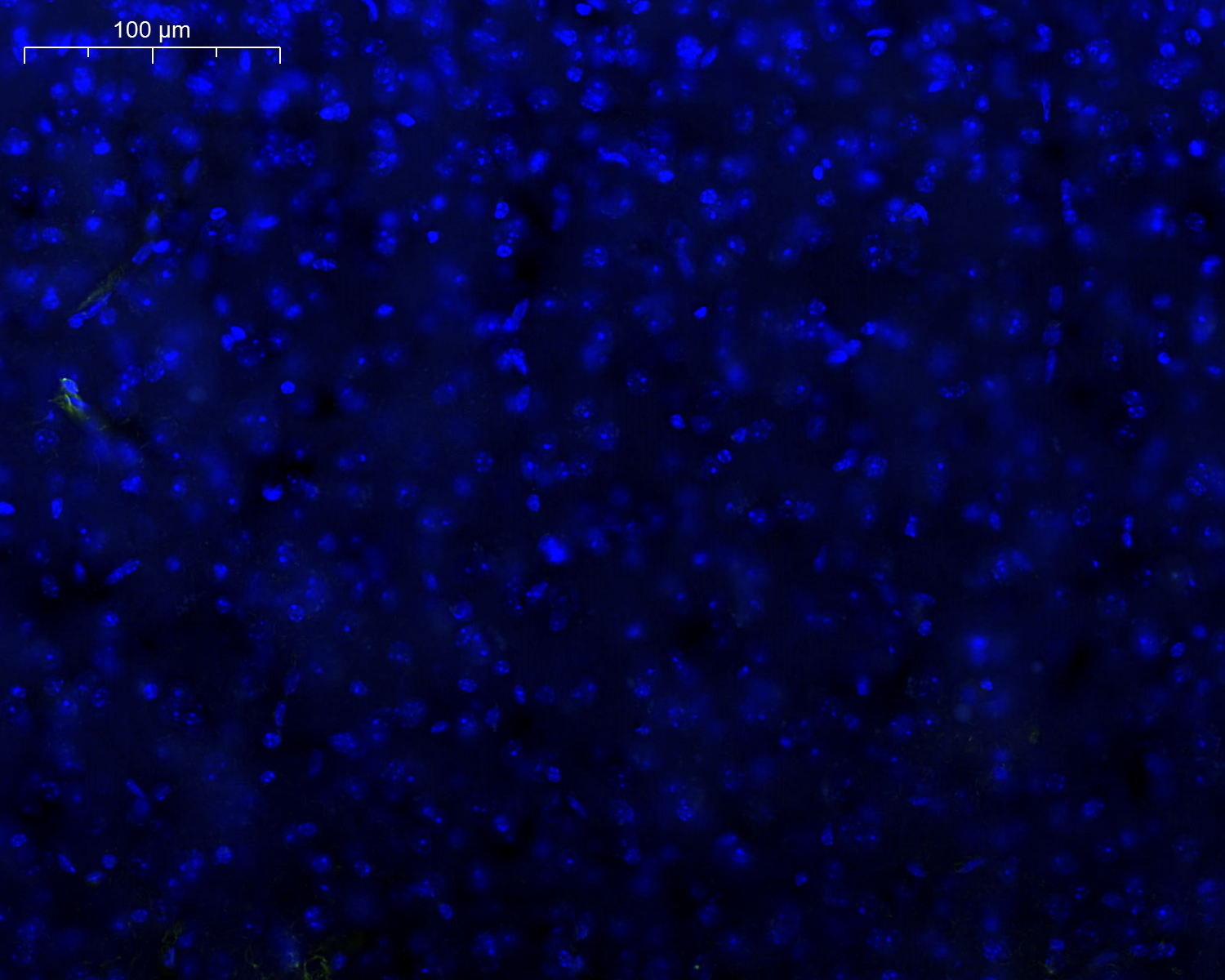

Supplement: Supplementary file 10 — Source data Fig. 7 [file 44321_2024_92_MOESM10_ESM.zip › Figure 7/7A/WT PIC_Cortex_GFAP_40x.jpg]

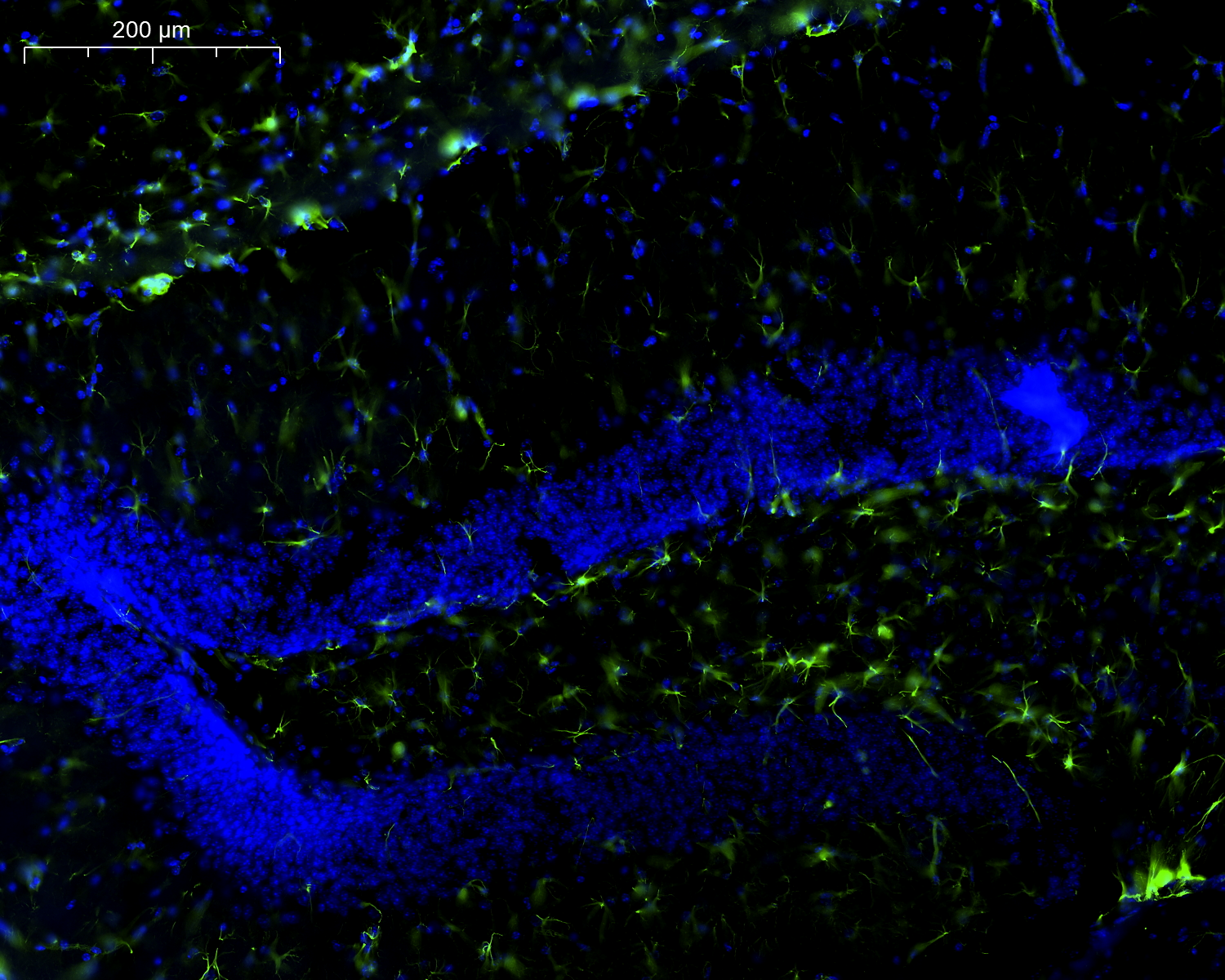

Supplement: Supplementary file 10 — Source data Fig. 7 [file 44321_2024_92_MOESM10_ESM.zip › Figure 7/7A/WT PIC_Hippo_GFAP_20.0x.tif]

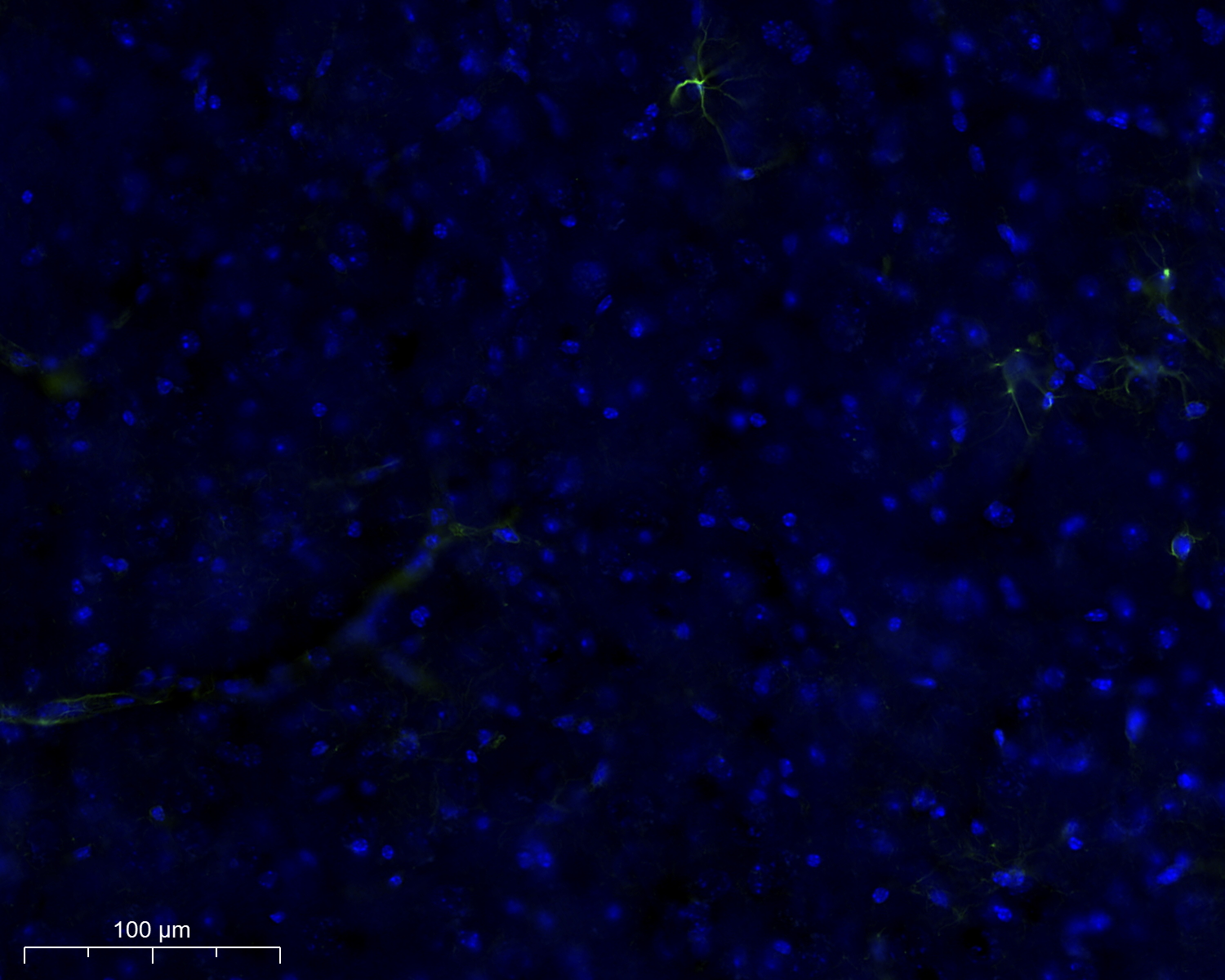

Supplement: Supplementary file 10 — Source data Fig. 7 [file 44321_2024_92_MOESM10_ESM.zip › Figure 7/7A/WT_Amy_GFAP_40.0x.jpg]

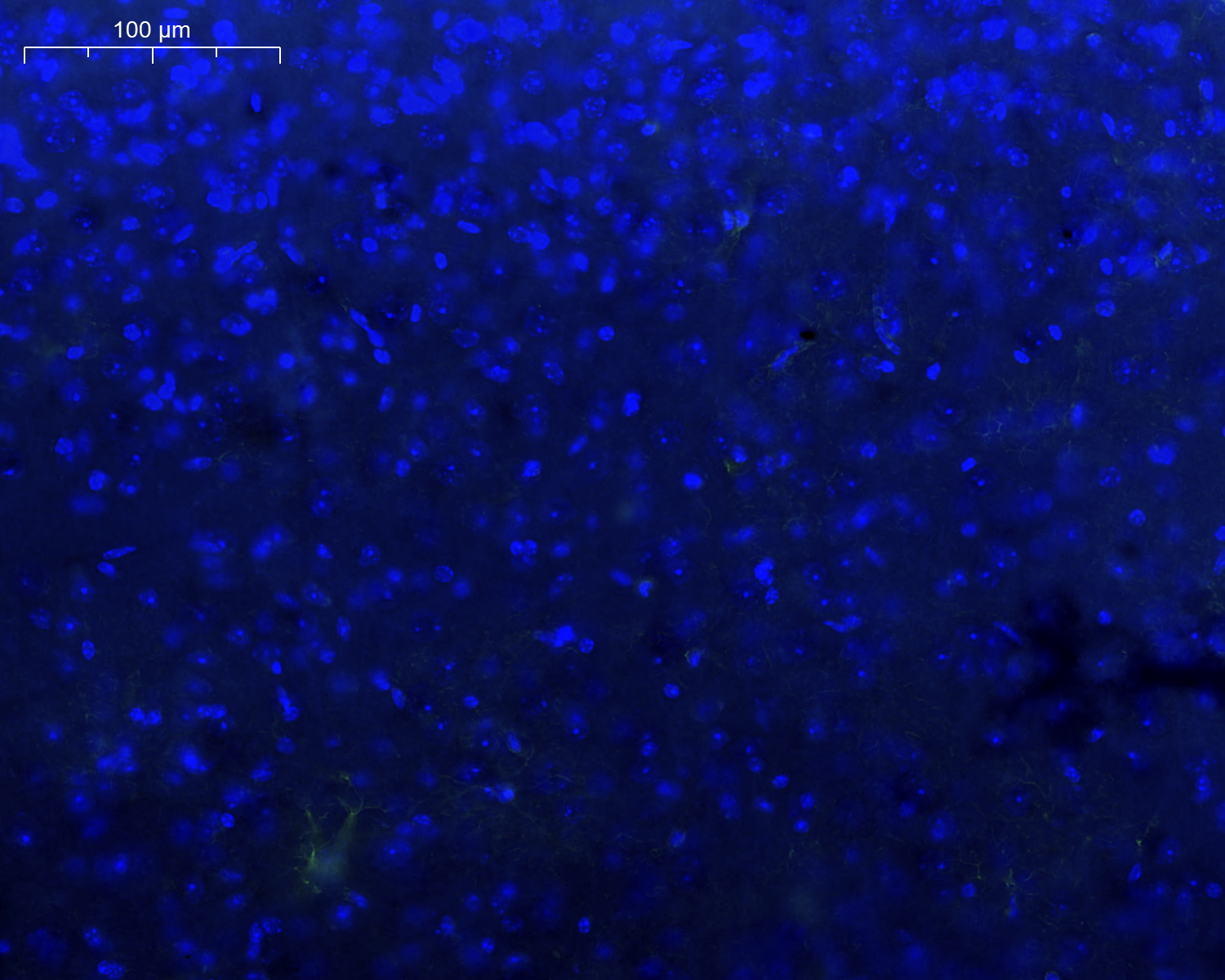

Supplement: Supplementary file 10 — Source data Fig. 7 [file 44321_2024_92_MOESM10_ESM.zip › Figure 7/7A/WT_Cortex_GFAP_40x.jpg]

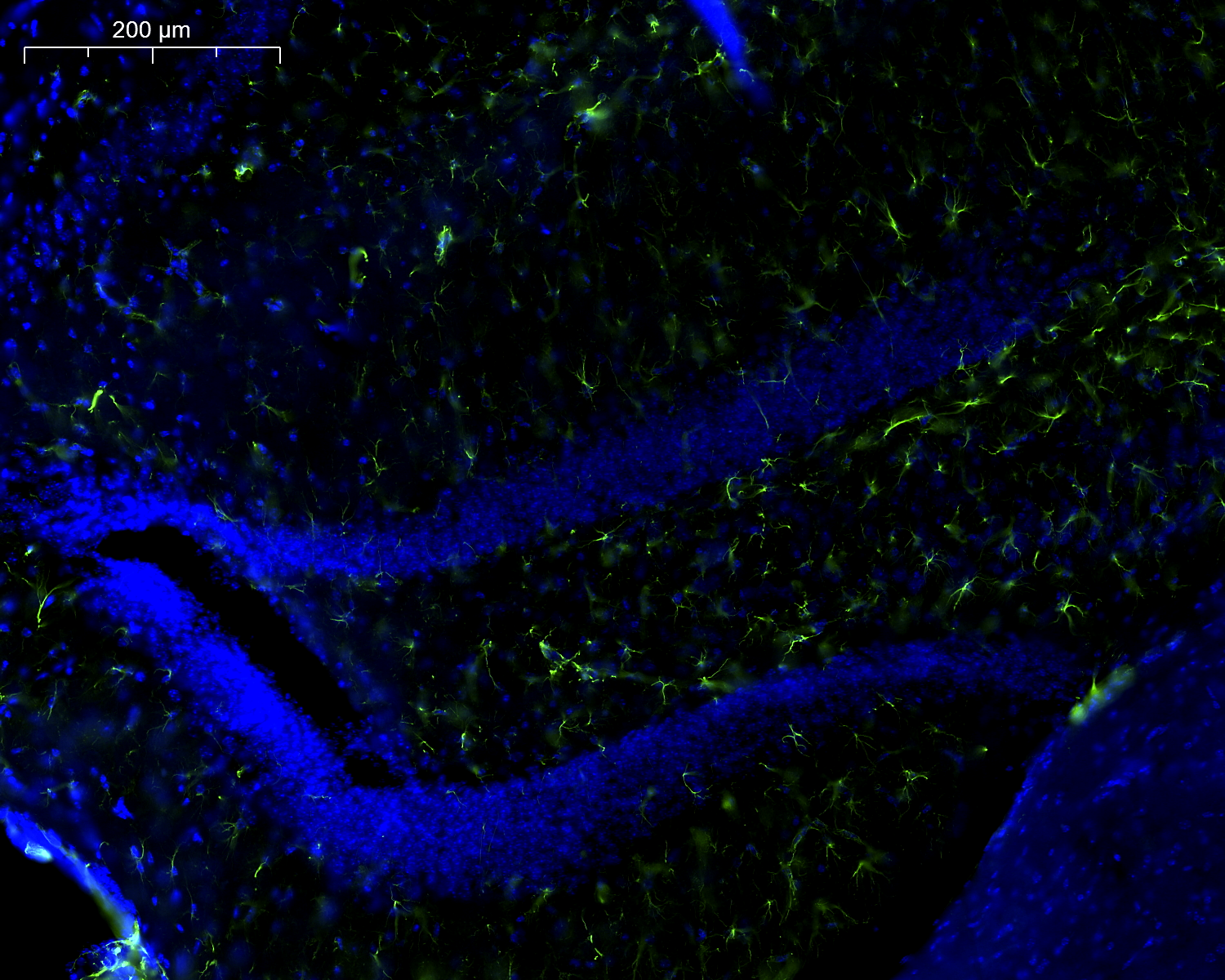

Supplement: Supplementary file 10 — Source data Fig. 7 [file 44321_2024_92_MOESM10_ESM.zip › Figure 7/7A/WT_Hippo_GFAP_20.0x.tif]

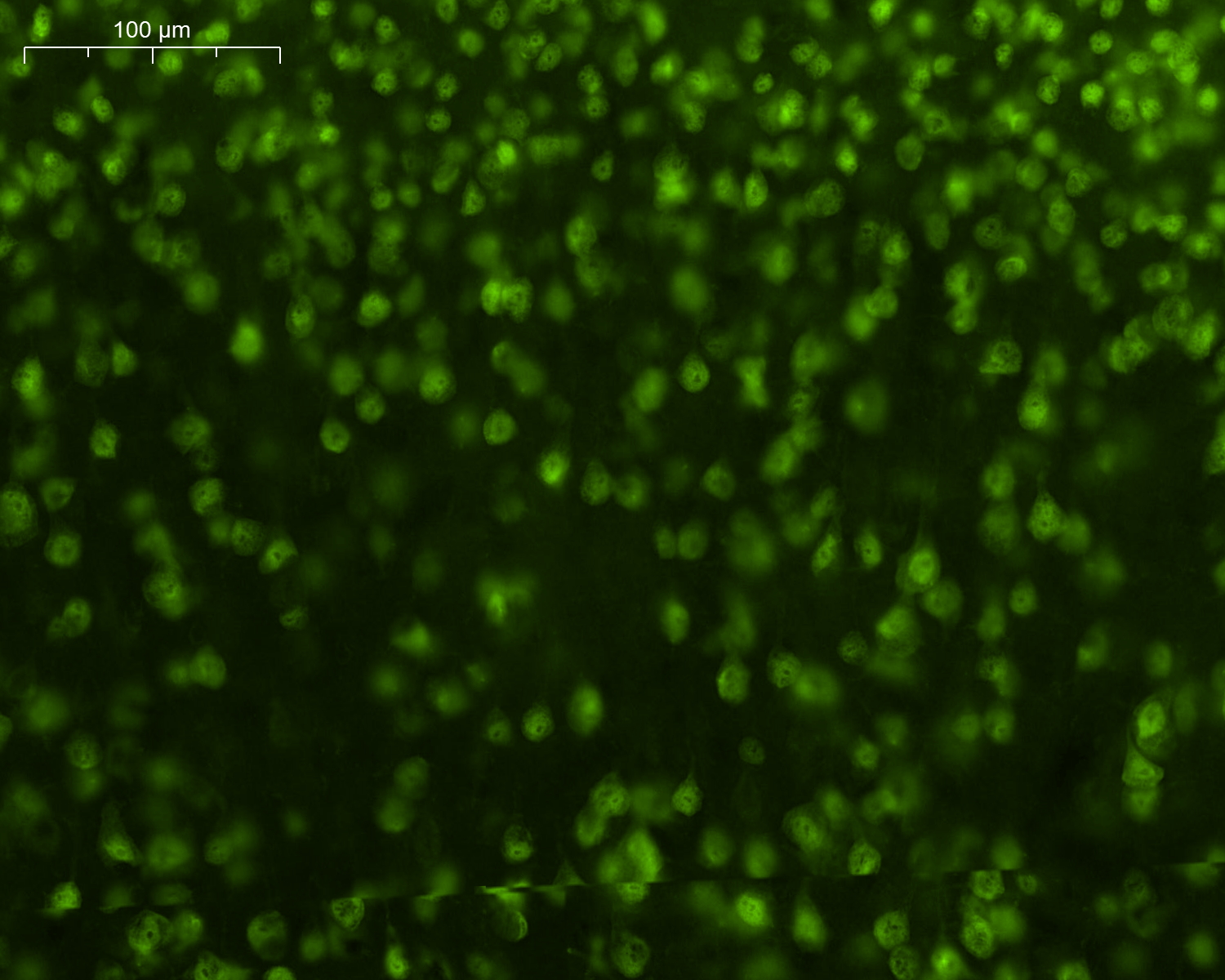

Supplement: Supplementary file 11 — Source data Fig. 8 [file 44321_2024_92_MOESM11_ESM.zip › Figure 8/8A/MPS PIC_Cortex_NeuN_40x.jpg]

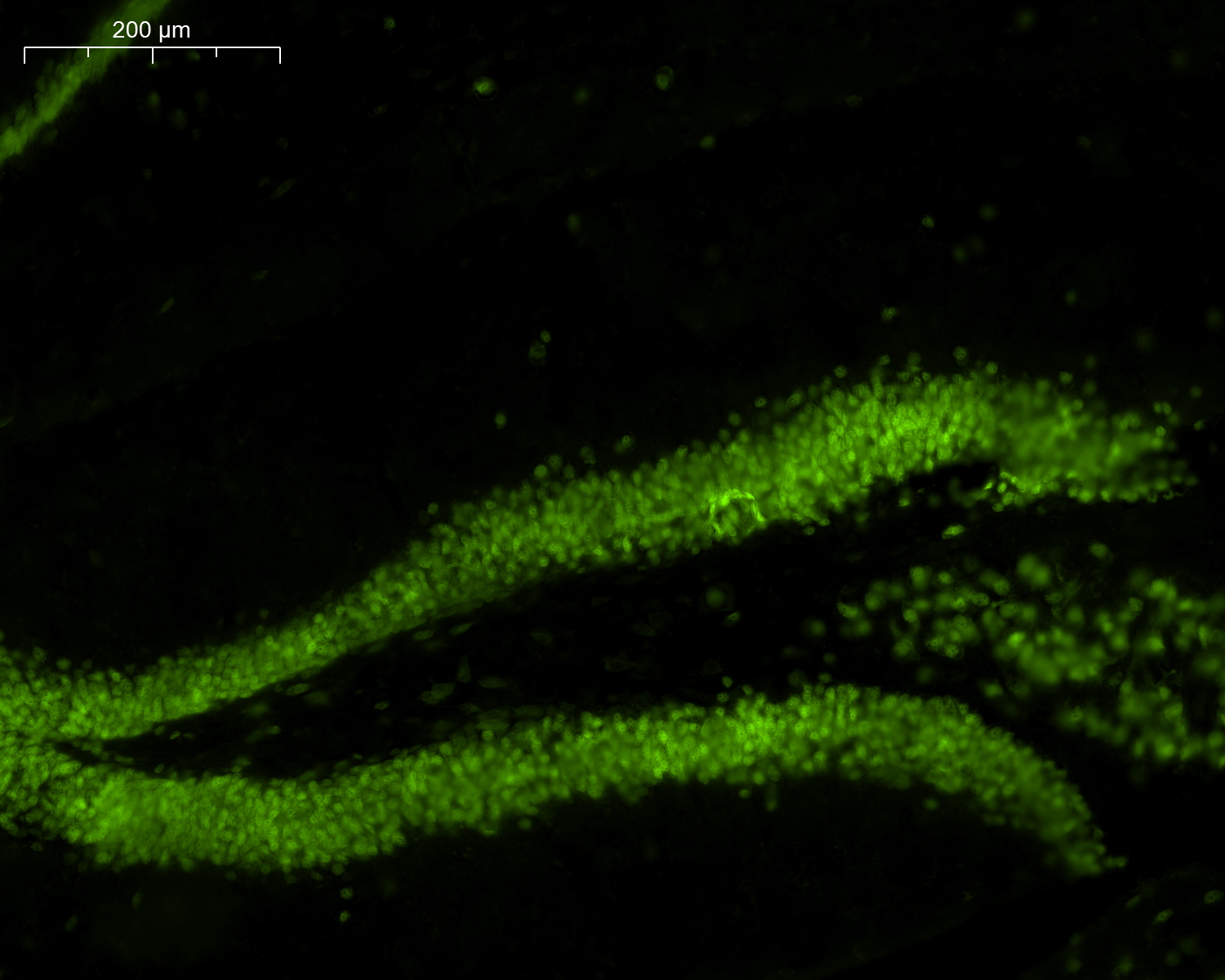

Supplement: Supplementary file 11 — Source data Fig. 8 [file 44321_2024_92_MOESM11_ESM.zip › Figure 8/8A/MPS PIC_Hippo_NeuN_20x.jpg]

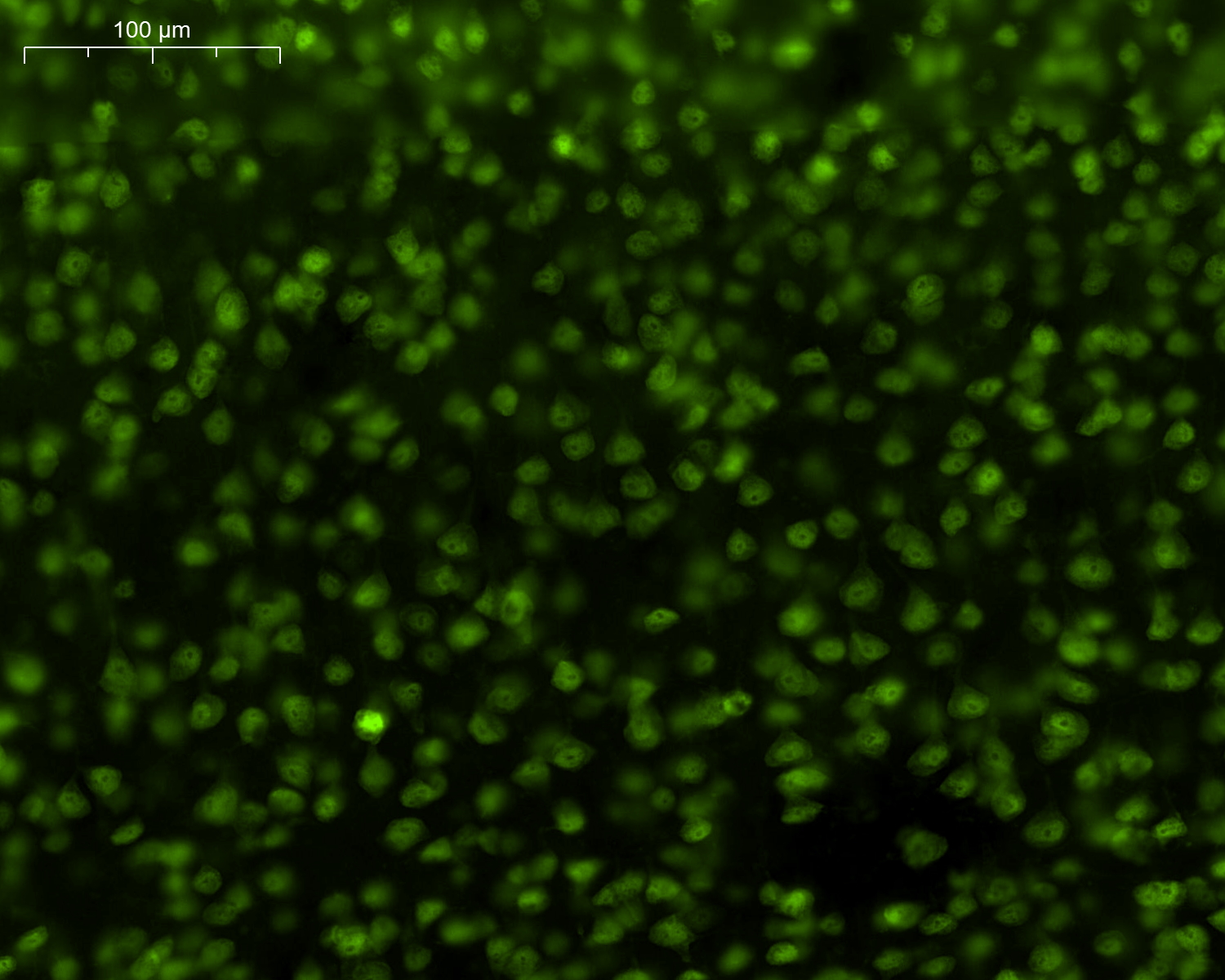

Supplement: Supplementary file 11 — Source data Fig. 8 [file 44321_2024_92_MOESM11_ESM.zip › Figure 8/8A/MPS_Cortex_NeuN_40x.jpg]

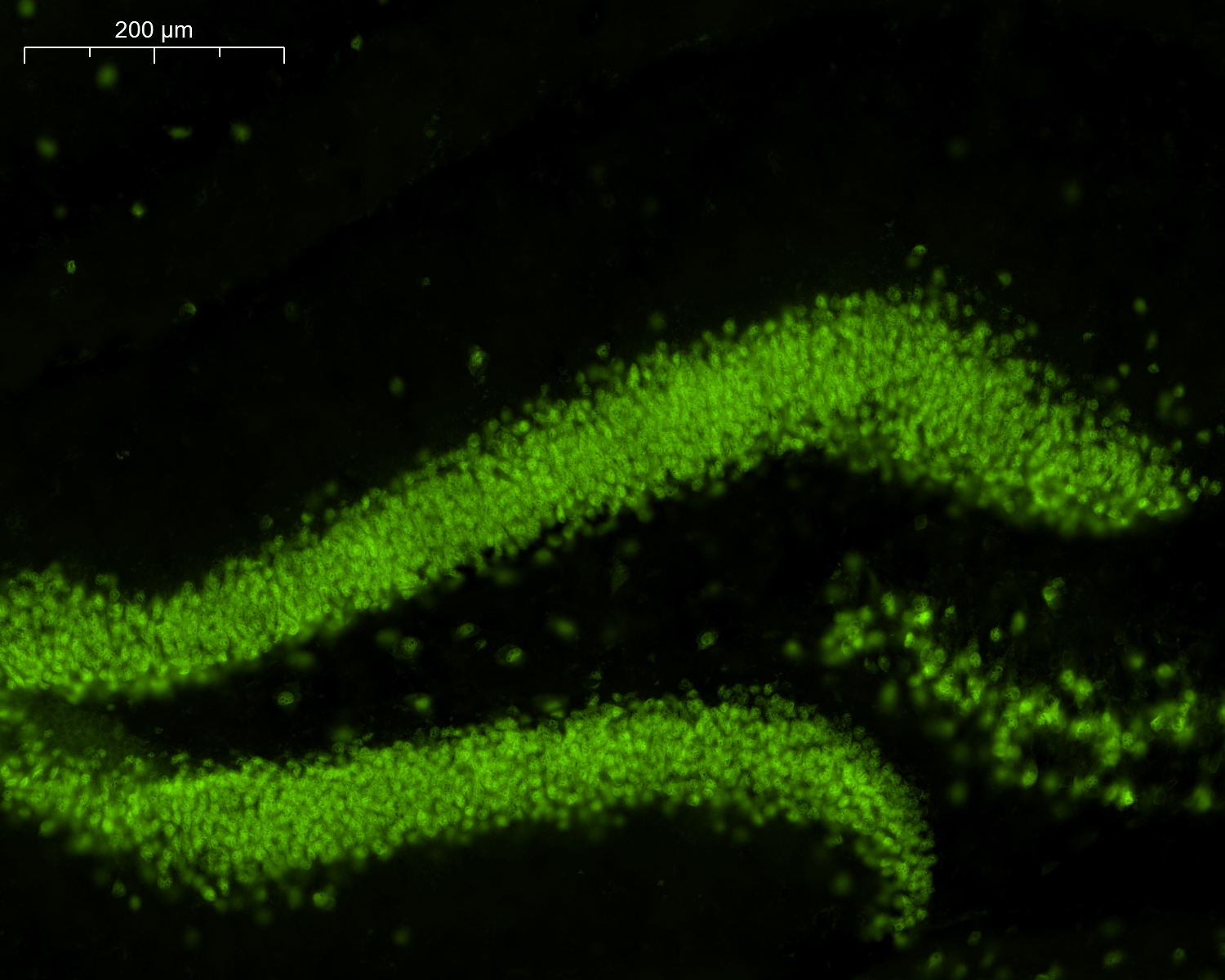

Supplement: Supplementary file 11 — Source data Fig. 8 [file 44321_2024_92_MOESM11_ESM.zip › Figure 8/8A/MPS_Hippo_NeuN_20x.jpg]

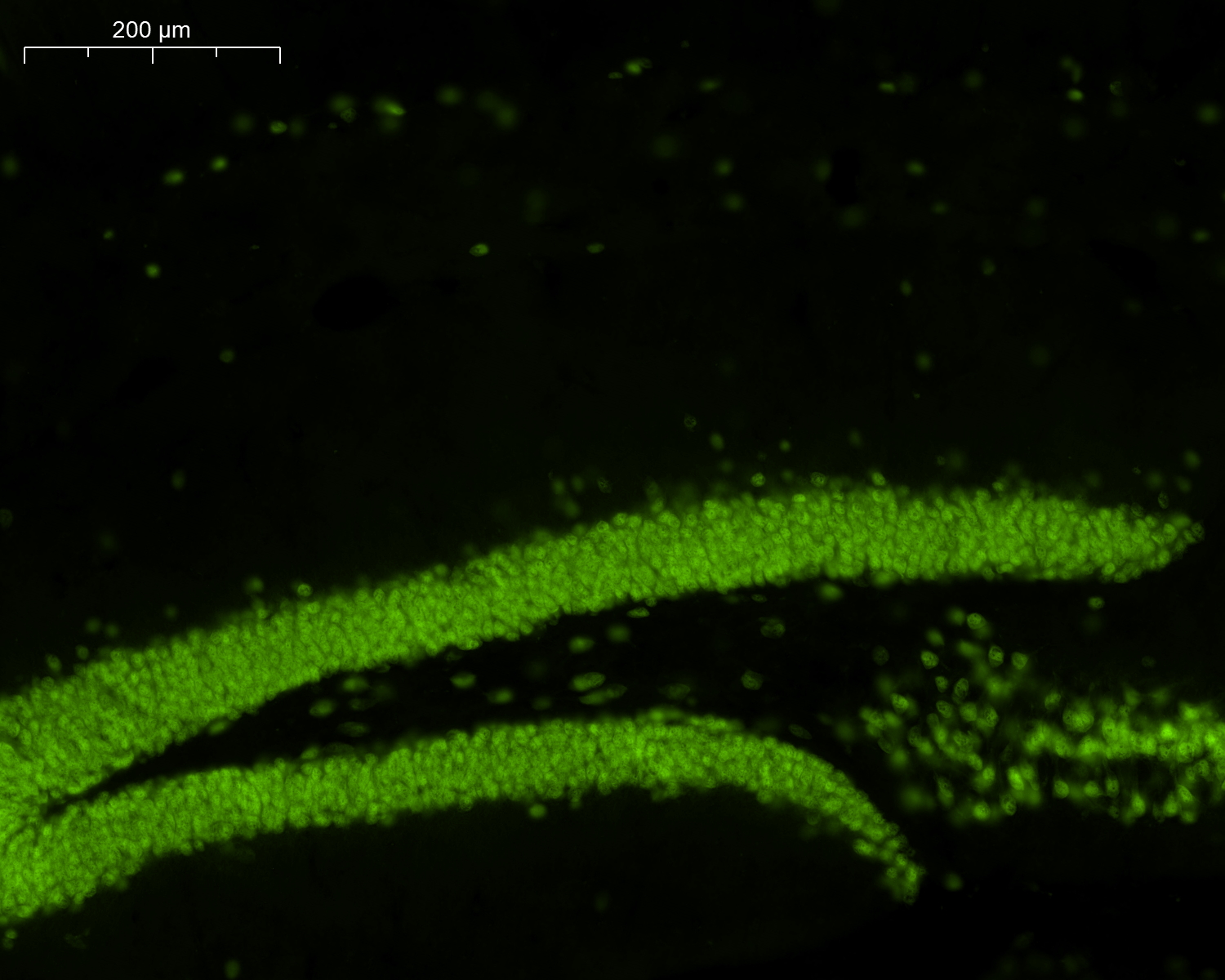

Supplement: Supplementary file 11 — Source data Fig. 8 [file 44321_2024_92_MOESM11_ESM.zip › Figure 8/8A/WT Hippo_NeuN_20x.jpg]

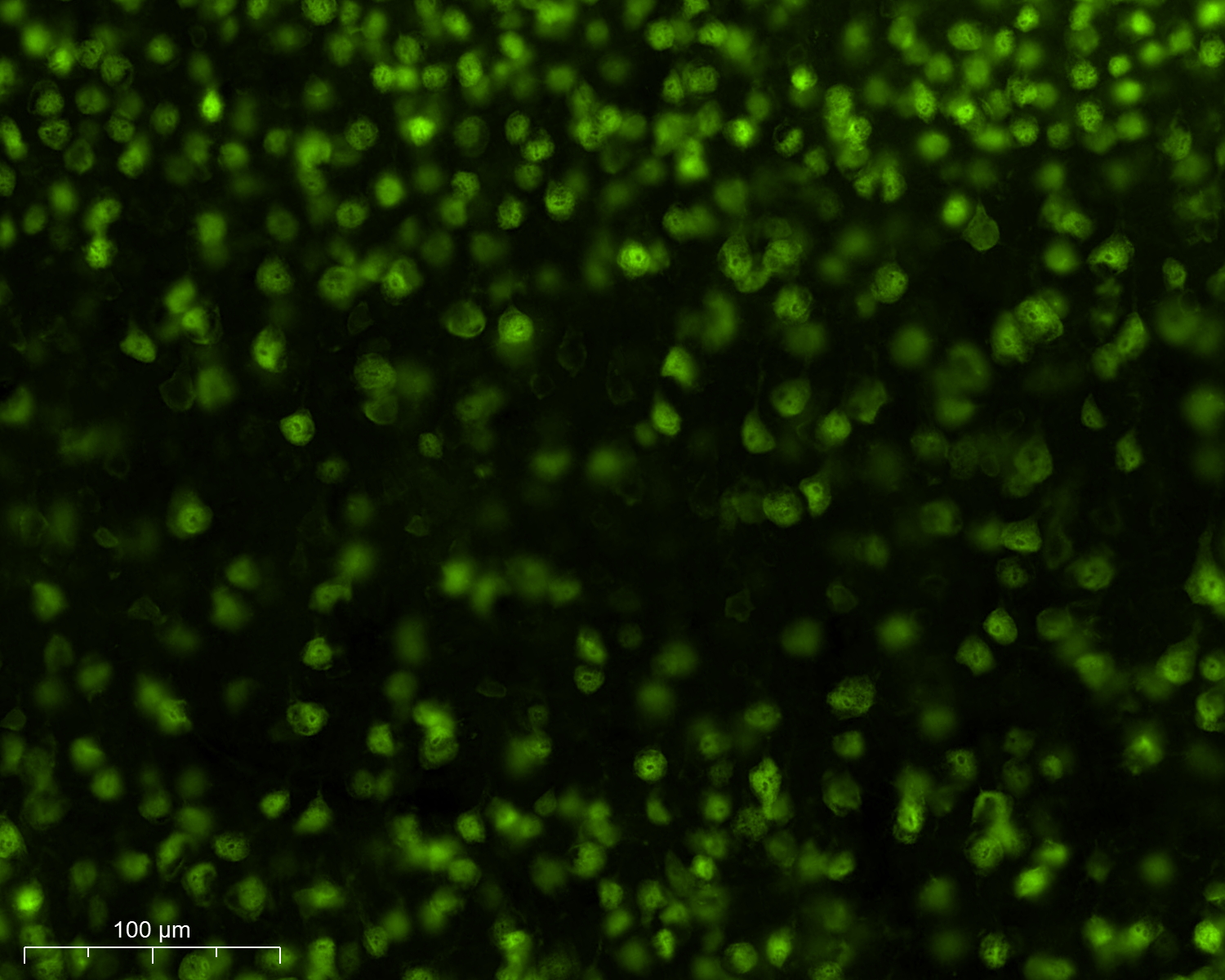

Supplement: Supplementary file 11 — Source data Fig. 8 [file 44321_2024_92_MOESM11_ESM.zip › Figure 8/8A/WT PIC_Cortex_NeuN_40.0x.jpg]

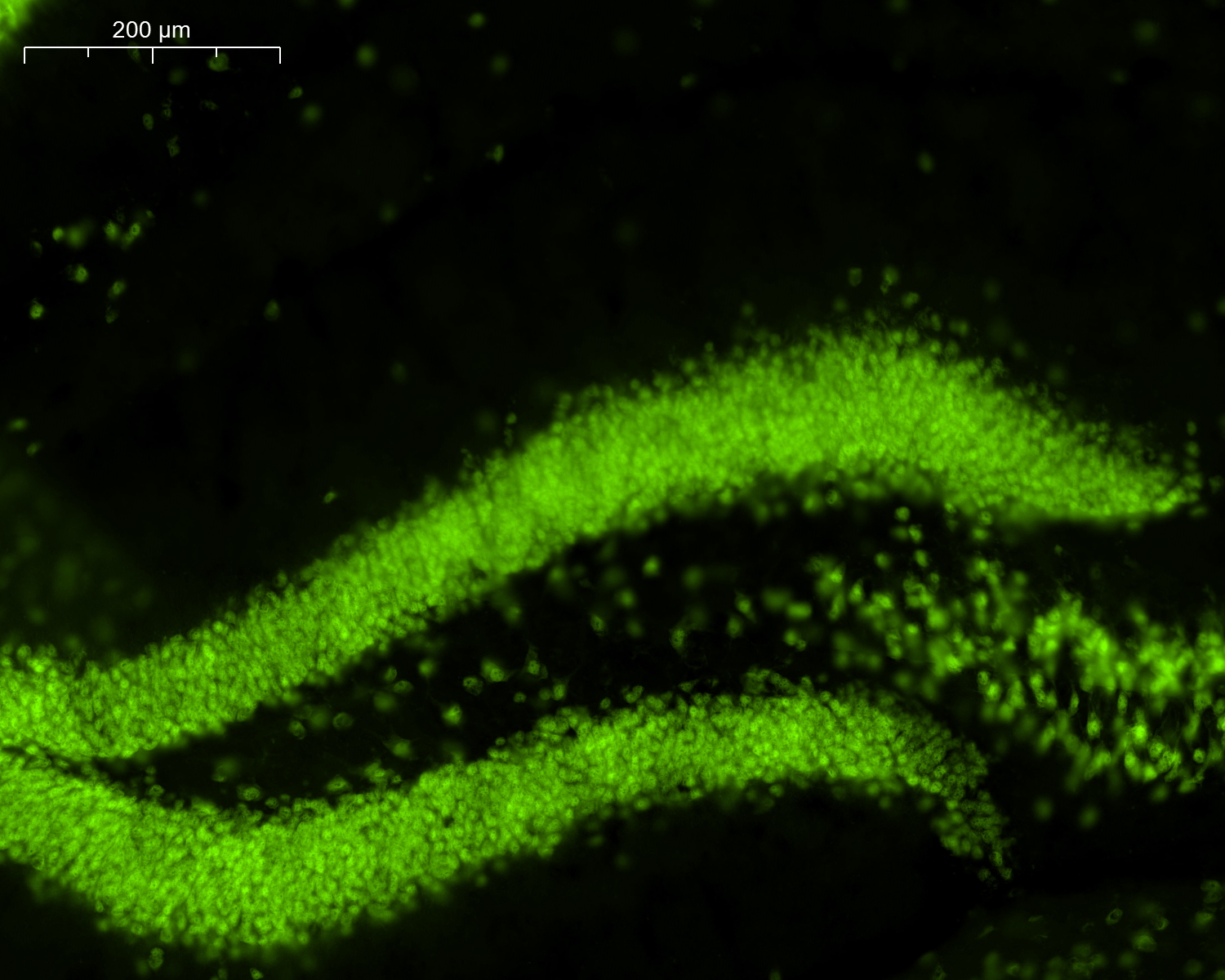

Supplement: Supplementary file 11 — Source data Fig. 8 [file 44321_2024_92_MOESM11_ESM.zip › Figure 8/8A/WT PIC_Hippo_NeuN_20x.jpg]

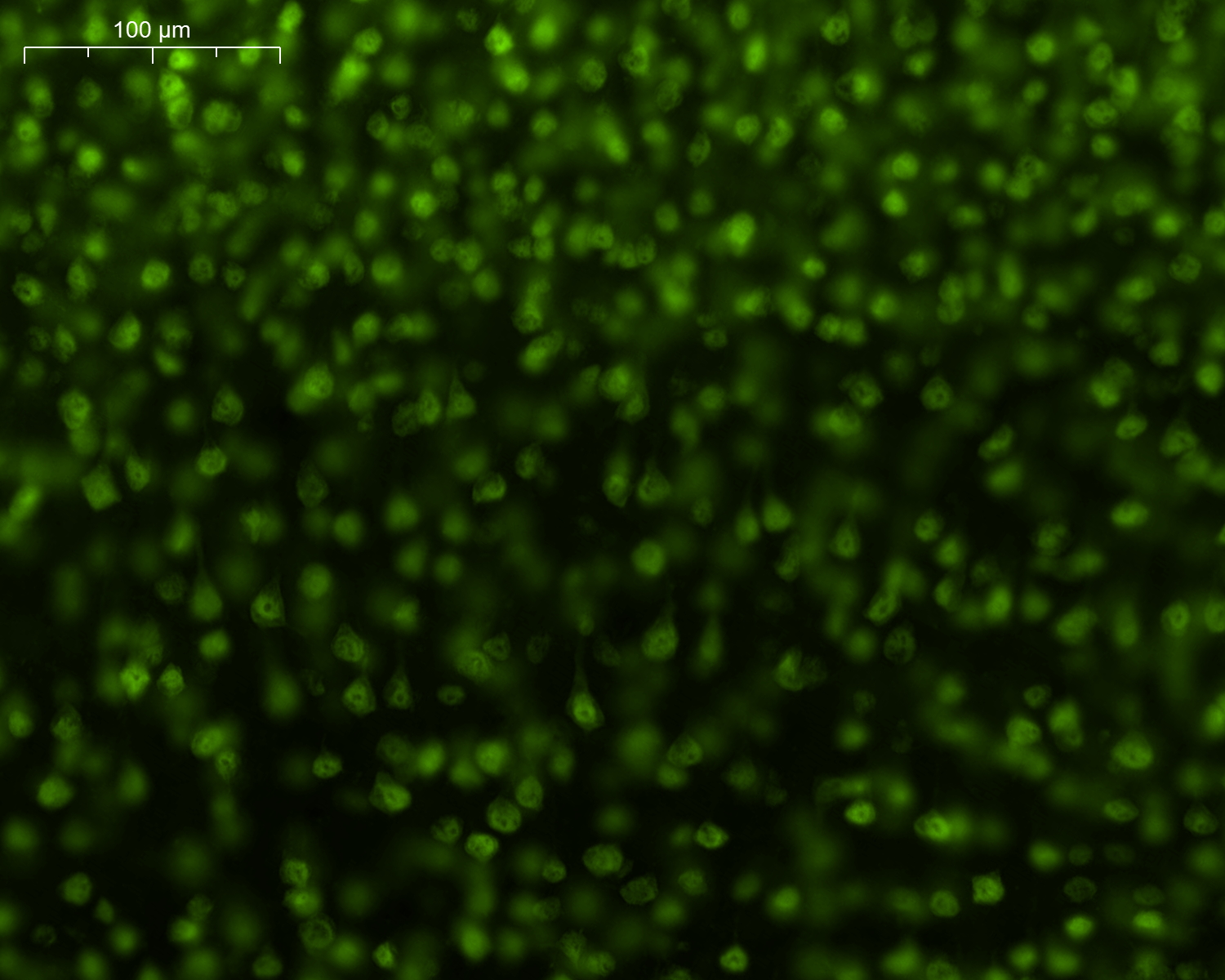

Supplement: Supplementary file 11 — Source data Fig. 8 [file 44321_2024_92_MOESM11_ESM.zip › Figure 8/8A/WT_Cortex_NeuN_40x.jpg]
