# Supplementary material for: Systemic immune challenge exacerbates neurodegeneration in a model of neurological lysosomal disease
Source: EMBO Mol Med. 2024 Jun 18;16(7):7. doi: 10.1038/s44321-024-00092-4 (PMC11251277; doi:10.1038/s44321-024-00092-4)
Supplement: Supplementary file 12 — Expanded View Figures [file 44321_2024_92_MOESM12_ESM.pdf]

Expanded View Figures

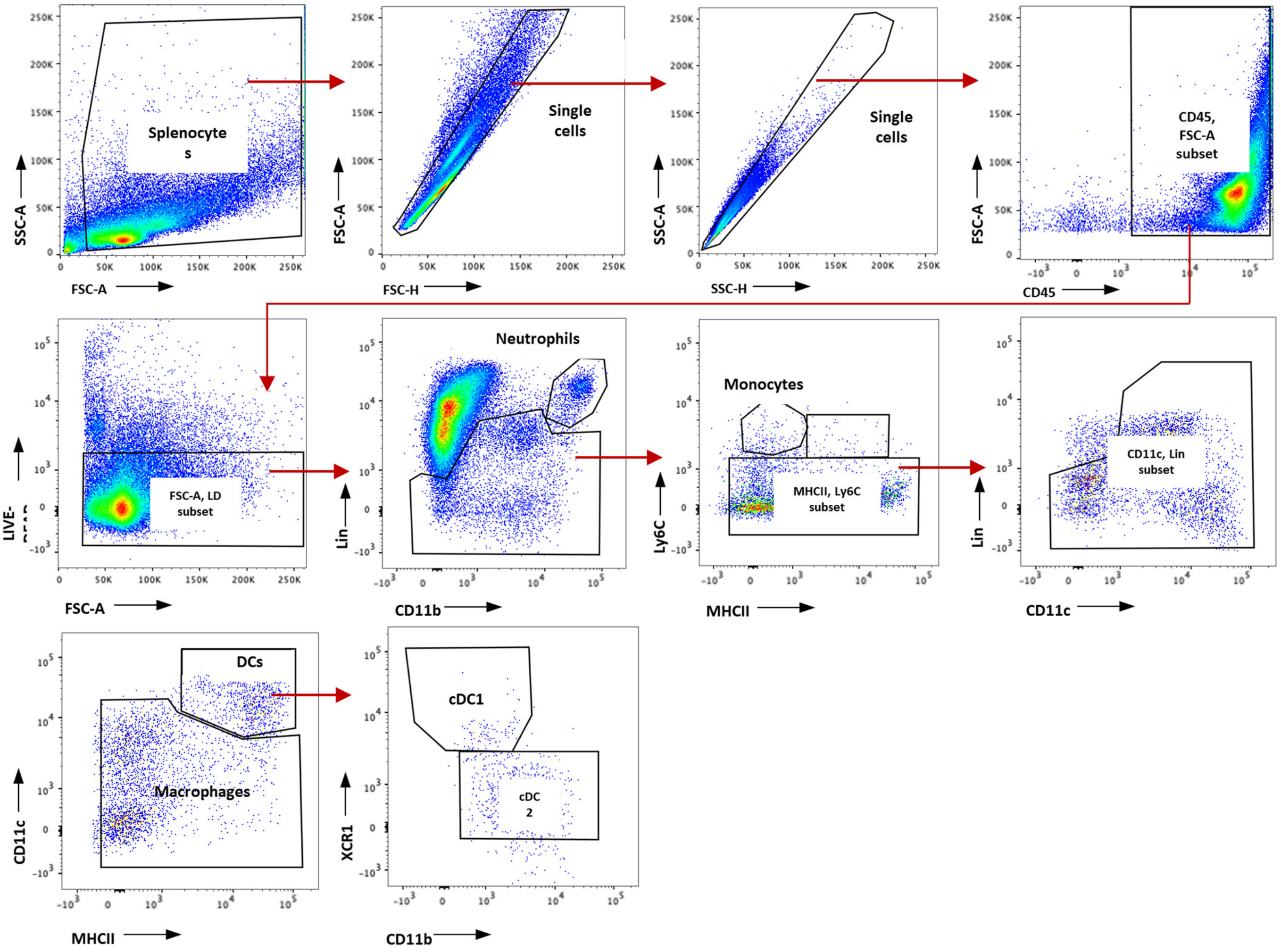

Figure EV1. Gating strategy for innate immune cells.

Whole tissue digests were prepared following tissue harvest. Cells were stained with CD45, Live/Dead, Lineage, CD11b, MHCII, Ly6C, CD11c and XRC1. Lymphocytes were initially gated on a forward scatter/side scatter by excluding the cells which displayed a size lower than 30k and were identified based on a linear relationship between FSC-H and FSC-A, followed by SSC-H and SSC-A. Leukocytes were defined as CD45 + , and live leukocytes were selected based on low staining for Live/Dead Zombie UV. From this innate immune cells were selected as follows: neutrophils CD45 + /CD11b + /Lin + , monocytes CD45 + /CD11b + /Lin + /Ly6C + /MHCII + , macrophages and DCs CD45 + /CD11b + /Lin + /Ly6C + /MHCII + /CD11c + .

© The Author(s)

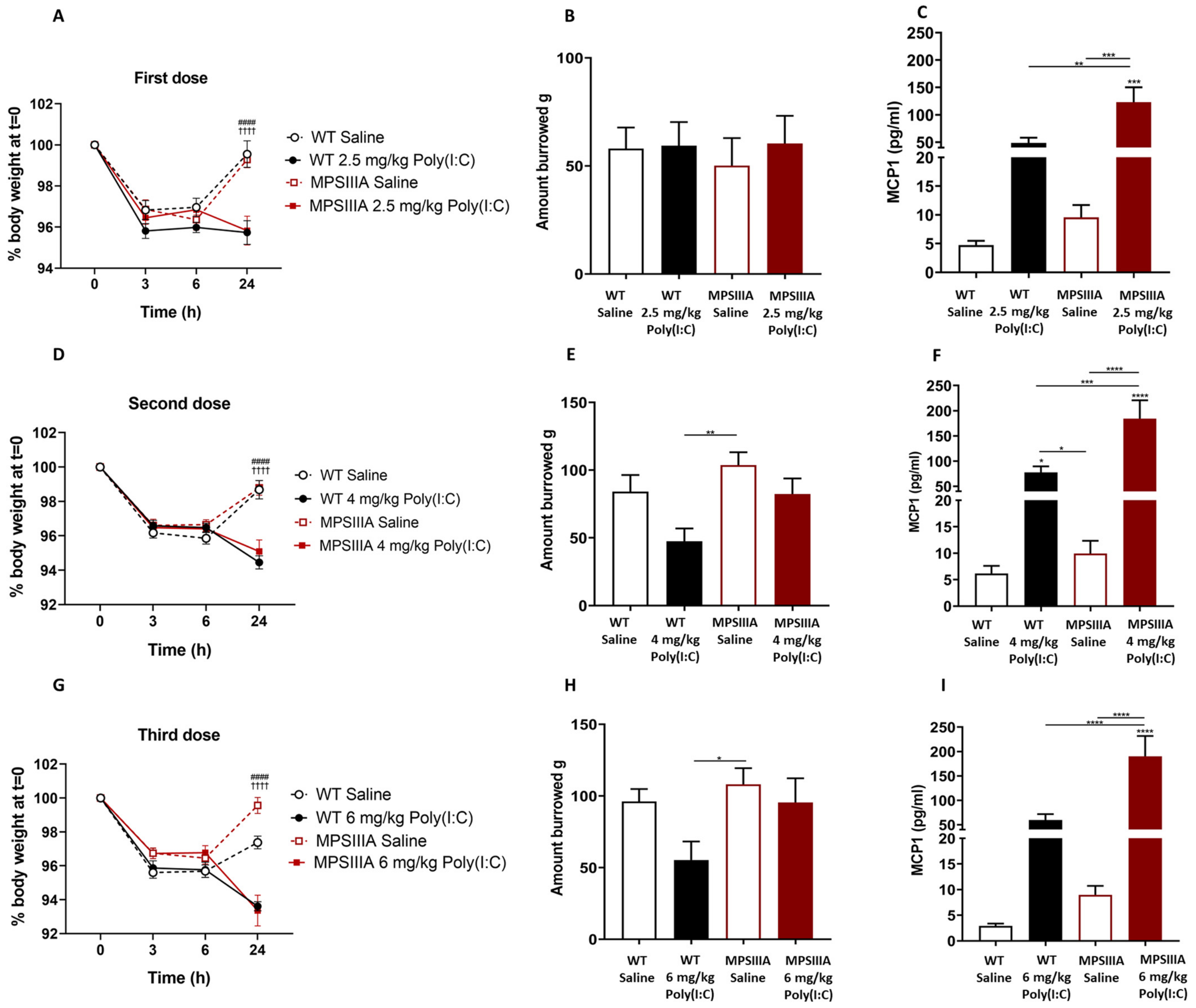

Figure EV2. Chronic administration of low poly(I:C) doses induces a sickness behaviour response.

(**A**) Body weight was measured at the time of poly(I:C) or saline administration ($t = 0$) and again at 3, 6 or 24 h post 2.5 mg/kg poly(I:C) or saline. Body weight is presented as a % of body weight at $t = 0$. (**B**) Burrowing was assessed between 1–3 h, post 2.5 mg/kg poly(I:C) or saline challenge in WT and MPS IIIA animals. (**C**) Levels of MPC1 chemokine were analysed in plasma collected at 24 h after challenge with saline or 2.5 mg/kg, poly(I:C).Levels of MCP-1 were evaluated via ELISA ($n = 10$–11). (**D**) Body weight was measured at the time of poly(I:C) or saline administration ($t = 0$) and again at 3, 6 or 24 h post 4 mg/kg poly(I:C) or saline. Body weight is presented as a % of body weight at $t = 0$. (**E**) Burrowing was assessed between 1 and 3 h, post 4 mg/kg poly(I:C) or saline challenge in WT and MPS IIIA animals. (**F**) Levels of MPC1 chemokine were analysed in plasma collected at 24 h after challenge with saline or 4 mg/kg, poly(I:C). Levels of MCP-1 were evaluated via ELISA ($n = 10$–11). (**G**) Body weight was measured at the time of poly(I:C) or saline administration ($t = 0$) and again at 3, 6 or 24 h 6 mg/kg poly(I:C) or saline. Body weight is presented as a % of body weight at $t = 0$. (**H**) Burrowing was assessed between 1–3 h, post 6 mg/kg poly(I:C) or saline challenge in WT and MPS IIIA animals. (**I**) Levels of MPC1 chemokine were analysed in plasma collected at 24 h after challenge with saline or 2.5 mg/kg, poly(I:C).Levels of MCP-1 were evaluated via ELISA ($n = 10$–11). Error bars represent standard error of the mean (SEM). Significant differences are determined by two-way ANOVA with Tukey's post hoc analysis. Comparisons showing significant differences are denoted by $^*P < 0.05$, $^{**}P < 0.01$, $^{***}P < 0.001$ and $^{****}P < 0.0001$ above comparison lines; significance against WT saline is shown above recorded data (**B**, **C**, **E**, **F**, **H**, **I**). # indicates poly(I:C) being significantly different from saline for WT animals; † indicates poly(I:C) being significantly different from saline for MPS IIIA animals, whereby $^{####}$ or $^{††††}P < 0.0001$ above comparison lines (**A**, **D**, **G**).

 © The Author(s)

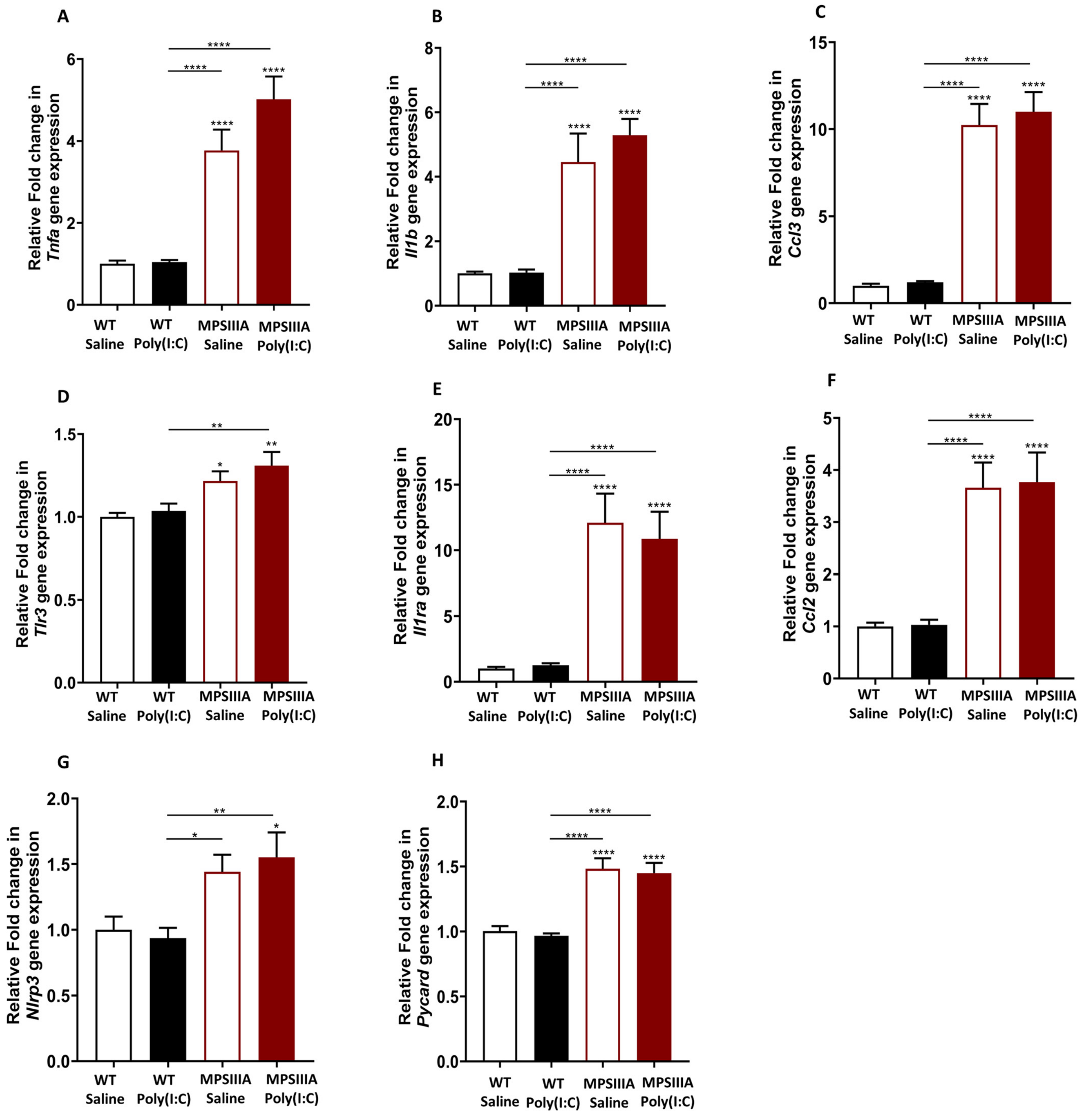

Figure EV3. Chronic poly(I:C) leads to increased significance of expression of several key pro-inflammatory genes in the CNS of MPS IIIA mice.

Expression of brain mRNA for *Tnfa* (**A**), *Il1b* (**B**), *Ccl3* (**C**), *Tlr3* (**D**), *Il1ra* (**E**), *Ccl2* (**F**), *Nlrp3* (**G**), and *Pycard* (**H**) in WT and MPS IIIA mice either treated with saline or with increasing doses of poly(I:C) ($n = 10$–11 per group). mRNA levels were measured by quantitative PCR and normalised to GAPDH. Error bars represent standard error of the mean (SEM). Significant differences are determined by two-way ANOVA with Tukey's post hoc analysis. Comparisons showing significant differences are denoted by *$P < 0.05$, **$P < 0.01$ and ****$P < 0.0001$ above comparison lines. Significance against WT saline is shown above recorded data.

© The Author(s)

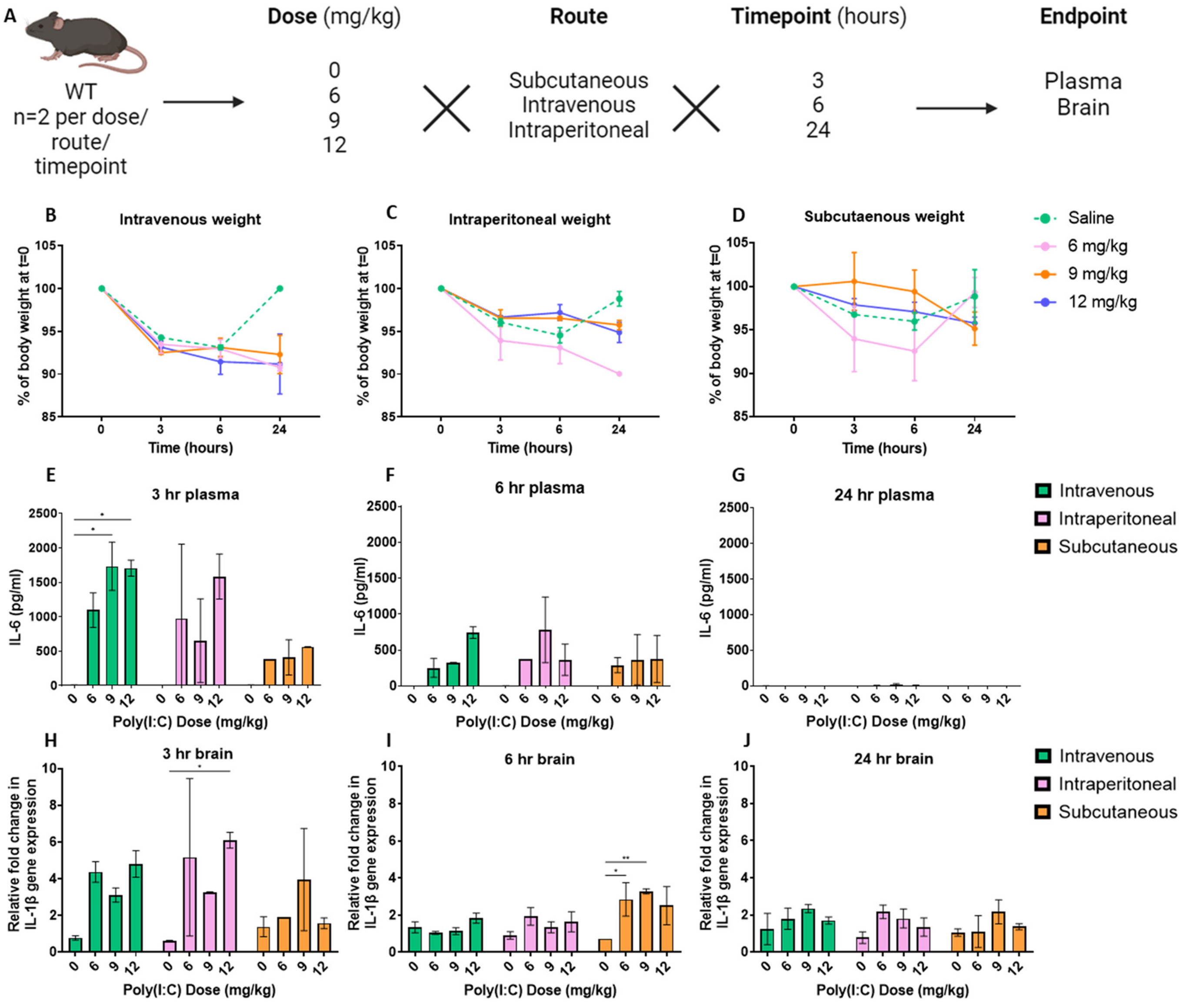

Figure EV4. Poly(I:C) induces a transient immune response via different delivery routes.

(**A**) 8-week-old WT mice were injected with poly(I:C) or saline via subcutaneous, intravenous or intraperitoneal injection. Doses were 6, 9 or 12 mg/kg poly(I:C) or an equivalent volume of saline ($n = 2$). (**B–D**) Body weight was measured prior to injection ($t = 0$), and at 3, 6 and 24 h post injection ($n = 2$). Data are plotted as mean % body weight at $t = 0$ and error bars indicate standard deviation (SD). (**E–G**) Blood was drawn by cardiac puncture at 3, 6 and 24 h post injection. Plasma was separated and tested for IL-6 with ELISA ($n = 2$). (**H–J**) Brain tissue was harvested at 3, 6 and 24 h post injection. Protein was isolated by sonication and tested for IL-1β with ELISA. Data are presented as mean and error bars indicate SD. ($n = 2$). Significant differences were determined by two-way ANOVA with Tukey's post hoc analysis. $^{*}P < 0.05$ and $^{**}P < 0.01$.

 © The Author(s)
